# Biogeographic history of a large clade of ectomycorrhizal fungi, the Russulaceae, in the Neotropics and adjacent regions

**DOI:** 10.1111/nph.18365

**Published:** 2022-07-30

**Authors:** Jan Hackel, Terry W. Henkel, Pierre‐Arthur Moreau, Eske De Crop, Annemieke Verbeken, Mariana Sà, Bart Buyck, Maria‐Alice Neves, Aída Vasco‐Palacios, Felipe Wartchow, Heidy Schimann, Fabian Carriconde, Sigisfredo Garnica, Régis Courtecuisse, Monique Gardes, Sophie Manzi, Eliane Louisanna, Mélanie Roy

**Affiliations:** ^1^ Royal Botanic Gardens, Kew Richmond‐upon‐Thames TW9 3AE UK; ^2^ Laboratoire Evolution et Diversité Biologique (UMR 5174) Université Toulouse III – Paul Sabatier/CNRS/IRD 31062 Toulouse cedex 9 France; ^3^ Department of Biological Sciences California State Polytechnic University, Humboldt Arcata CA 95521 USA; ^4^ Faculté de Pharmacie, Laboratoire des Sciences Végétales et Fongiques (LGCgE, ER4) Université de Lille 59006 Lille France; ^5^ Department of Biology Ghent University 9000 Gent Belgium; ^6^ Centro Universitário de João Pessoa PB 58053‐000 João Pessoa Brazil; ^7^ Institut de Systématique, Évolution, Biodiversité (ISYEB), Muséum National d'Histoire Naturelle, CNRS Sorbonne Université, EPHE, Université des Antilles 75231 Paris cedex 05 France; ^8^ Departamento de Botânica Universidade Federal de Santa Catarina SC 88040‐900 Florianópolis Brazil; ^9^ Microbiología Ambiental–School of Microbiology, Laboratory of Taxonomy and Ecology of Fungi–Institute of Biology University of Antioquia 050010 Medellín Colombia; ^10^ Departamento de Sistemática e Ecologia Universidade Federal da Paraíba PB 58051‐970 João Pessoa Brazil; ^11^ UMR Ecologie des Forêts de Guyane AgroParisTech/CIRAD/CNRS/Université des Antilles/Université de la Guyane/INRA 97379 Kourou cedex French Guiana; ^12^ Institut Agronomique néo‐Calédonien (IAC), Equipe Sol & Végétations (SolVeg) BP18239 98848 Nouméa New Caledonia; ^13^ Instituto de Bioquímica y Microbiología Universidad Austral de Chile 5049000 Valdivia Chile; ^14^ Instituto Franco‐Argentino para el Estudio del Clima y sus Impactos (UMI IFAECI/CNRS‐CONICET‐UBA‐IRD) Universidad de Buenos Aires C1428EGA Ciudad Autonoma de Buenos Aires Argentina

**Keywords:** boreotropical migration, dispersal, diversification, ectomycorrhizal fungi, Neotropics, Patagonia, Russulaceae, vicariance

## Abstract

- The biogeography of neotropical fungi remains poorly understood. Here, we reconstruct the origins and diversification of neotropical lineages in one of the largest clades of ectomycorrhizal fungi in the globally widespread family Russulaceae.
- We inferred a supertree of 3285 operational taxonomic units, representing worldwide internal transcribed spacer sequences. We reconstructed biogeographic history and diversification and identified lineages in the Neotropics and adjacent Patagonia.
- The ectomycorrhizal Russulaceae have a tropical African origin. The oldest lineages in tropical South America, most with African sister groups, date to the mid‐Eocene, possibly coinciding with a boreotropical migration corridor. There were several transatlantic dispersal events from Africa more recently. Andean and Central American lineages mostly have north‐temperate origins and are associated with North Andean uplift and the general north–south biotic interchange across the Panama isthmus, respectively. Patagonian lineages have Australasian affinities. Diversification rates in tropical South America and other tropical areas are lower than in temperate areas.
- Neotropical Russulaceae have multiple biogeographic origins since the mid‐Eocene involving dispersal and co‐migration. Discontinuous distributions of host plants may explain low diversification rates of tropical lowland ectomycorrhizal fungi. Deeply diverging neotropical fungal lineages need to be better documented.

## Introduction

A fundamental challenge of evolutionary biology is to determine the drivers of the exceptional neotropical biodiversity (Antonelli & Sanmartín, 2011; Hughes *et al*., 2013; Antonelli *et al*., 2018; Palma‐Silva *et al*., 2022). Phylogenetic biogeography has shown how continental‐scale events such as the rise of the Andes (Antonelli *et al*., 2009; Hoorn *et al*., 2010; Pérez‐Escobar *et al*., 2022), biotic interchange between North and South America (Cody *et al*., 2010; Bacon *et al*., 2015), and transoceanic dispersal, including across the Atlantic from Africa (Pennington & Dick, 2004; Renner, 2004), have shaped extant diversity of neotropical plants and animals. Though some historical biogeographic studies have included neotropical fungi (Matheny *et al*., 2009; Leavitt *et al*., 2012; Wilson *et al*., 2012; Tedersoo *et al*., 2014b; Harrower *et al*., 2015; Amalfi, 2016), more are needed given the ecological importance of fungi as saprotrophs, pathogens, or mutualistic symbionts, which can influence the distribution of associated plants and vice versa (Peay *et al*., 2016).

Ectomycorrhizal (ECM) fungi are obligate mutualistic root symbionts, mainly of trees (Smith & Read, 2010; Tedersoo *et al*., 2020). They have global ecosystem importance by sustaining the biomass of the majority of trees worldwide (Soudzilovskaia *et al*., 2019; Steidinger *et al*., 2019). Unlike the majority of organisms, most ECM fungal groups peak in diversity and abundance in temperate latitudinal zones (Tedersoo & Nara, 2010; Tedersoo *et al*., 2014a; Steidinger *et al*., 2019). This suggests that they may have originated and have a longer history and/or have diversified faster in the temperate zone. Nonetheless, ECM trees are dominant in some tropical forests, where the symbiosis plays a key role in nutrient cycling and competitive abilities (Henkel, 2003; McGuire, 2007; Corrales *et al*., 2016, 2018; Carriconde *et al*., 2019; Henkel & Mayor, 2019; Hall *et al*., 2020). The importance of ECM fungi in the Neotropics is exemplified by the recent discoveries of high‐diversity sites with many undescribed species (e.g. Henkel *et al*., 2012; Roy *et al*., 2016; Vasco‐Palacios *et al*., 2018; Delgat *et al*., 2020; Corrales & Ovrebo, 2021). ECM fungi thus form an important component of many neotropical ecosystems.

The current geographic distribution of ECM fungi is the product of environment and host plant presence (van der Linde *et al*., 2018), as well as historical contingencies such as area of origin and dispersal limitation (Peay *et al*., 2016). The Neotropics contain phylogenetically diverse ECM host plants distributed across a variety of habitats and elevation zones, suggesting distinct biogeographic ECM domains. For example, ECM host plants in the neotropical lowlands are from predominantly tropical lineages in the Fabaceae, Cistaceae, Dipterocarpaceae, Polygonaceae, and Nyctaginaceae, whereas mainly north‐temperate Betulaceae, Fagaceae, and Juglandaceae occur at higher elevations in the Andes or Central America (Tedersoo, 2017; Tedersoo & Brundrett, 2017; Corrales *et al*., 2018). Patagonia, in southern South America, has yet another dominant host lineage, the Nothofagaceae, whose associated macromycota is quite removed from that of northern South America (Singer, 1953; Trierveiler‐Pereira *et al*., 2014), although Nothofagaceae were probably present at tropical latitudes in South America in the Eocene (Jaramillo *et al*., 2006).

Though many ECM fungal lineages have broad distributions (Tedersoo *et al*., 2014a), the drivers of their biogeographic spread have been difficult to identify. A major problem is distinguishing between continental vicariance, overland migration, or overseas dispersal. Moyersoen (2006) hypothesized that Africa–South American (i.e. Gondwanan) vicariance, between 120 and 90 million years ago (Ma) (Müller *et al*., 2016), explained the presence of *Pakaraimaea*, a phylogenetically distinct genus of ECM host plants, in South America. Africa–South America vicariance has, however, been refuted for most other groups of plants (Pennington & Dick, 2004), including the pantropical ECM gymnosperm *Gnetum* (Won & Renner, 2006). It was also rejected for the ECM fungal genus *Inocybe* (Matheny *et al*., 2009). In turn, vicariance is likely responsible for the Australian–Patagonian (i.e. southern Gondwanan) disjunction of Nothofagaceae (Cook & Crisp, 2005). An alternative explanation, boreotropical migration across land routes when Palaeocene/Eocene tropical climates extended into high latitudes (Wolfe, 1975), was suggested for disjunctions in the north‐temperate host lineage Juglandaceae (Zhang *et al*., 2022) and the ECM fungal groups *Amanita* sect. *Caesarea* (Sánchez‐Ramírez *et al*., 2014) and Sclerodermatinae (Wilson *et al*., 2012). Over the past 20 Myr, narrowing and closure of the Panama isthmus facilitated north–south dispersal (Bacon *et al*., 2015; O'Dea *et al*., 2016), and the rapid rise of the northern Andes 5–8 Ma created high‐altitude environments for north‐temperate arrivals (Pérez‐Escobar *et al*., 2022). The southward extensions of the north‐temperate ECM host genera *Alnus* and *Quercus* into the Andes are examples of this (Tedersoo, 2017).

An important group of mushroom‐forming ECM fungi is found in the Russulaceae (Russulales, Agaricomycetes, Basidiomycota), a speciose family that is globally distributed over low and high latitudes wherever ECM vegetation is found. Recent systematic work has clarified Russulaceae relationships (Buyck *et al*., 2008, 2018; Verbeken *et al*., 2014; Wisitrassameewong *et al*., 2016; De Crop *et al*., 2017; Wang *et al*., 2018): the four mushroom‐forming genera – *Lactarius*, *Lactifluus*, *Multifurca*, and *Russula*, along with several nested sequestrate genera – have > 4500 species (He *et al*., 2019) and form a monophyletic ECM lineage emerging from a small grade of saprotrophic genera (Looney *et al*., 2018, 2022). For simplicity, we refer to this ECM lineage (equivalent to ‘/russula‐lactarius’ *sensu* Tedersoo *et al*., 2010a) as ‘Russulaceae’ in the following. Russulaceae are routinely recovered in sporocarp and DNA‐based surveys, including in the Neotropics (Tedersoo *et al*., 2010b; Tedersoo & Nara, 2010; Smith *et al*., 2011; Henkel *et al*., 2012; Roy *et al*., 2016; Vasco‐Palacios *et al*., 2018; Corrales & Ovrebo, 2021). They associate with most known ECM plant lineages (Tedersoo & Brundrett, 2017), as well as orchids (Dearnaley, 2007).

**Fig. 1:**
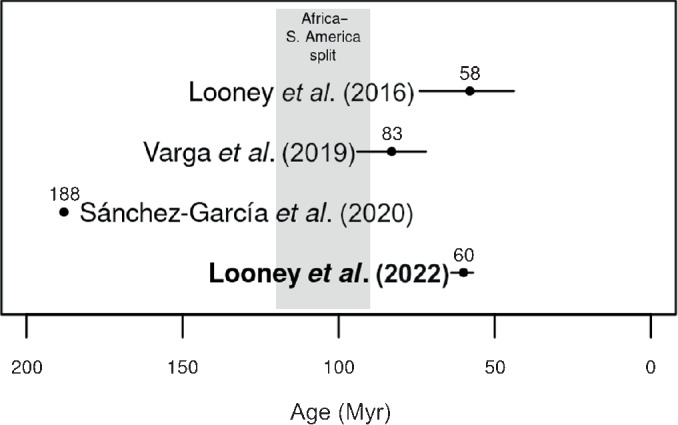
Overview of published crown age estimates for the ectomycorrhizal Russulaceae (*Lactarius*, *Lactifluus*, *Multifurca*, and *Russula*). The Looney *et al*. (2022) estimate (in bold) was used for calibration in this study. Dots are median values/point estimates, error bars represent 95% posterior density intervals (Looney *et al*., 2016, 2022) and the range in 10 calibrated trees (Varga *et al*., 2019).

Most species of Russulaceae are temperate, including boreal, in distribution. This includes the majority of the largest genus *Russula*, for which a temperate origin and a higher temperate diversification rate relative to the Tropics has been inferred (Looney *et al*., 2016). However, a tropical African origin has long been posited for Russulaceae based on endemism and morphological distinctiveness (Pirozynski, 1983; Buyck *et al*., 1996). In the wider Neotropics, Russulaceae occur in all highland and lowland regions where ECM vegetation is found, and modern work has revealed a plethora of new species (Buyck & Ovrebo, 2002; Miller *et al*., 2002, 2012; Wartchow & Cavalcanti, 2010; Cheype & Campo, 2012; Sà *et al*., 2013, 2019; Sà & Wartchow, 2013, 2016; Wartchow *et al*., 2013; Montoya *et al*., 2014; Trierveiler‐Pereira *et al*., 2014; Crous *et al*., 2017; Delgat *et al*., 2020; Duque Barbosa *et al*., 2020; Silva‐Filho *et al*., 2020; Manz *et al*., 2021; Vera *et al*., 2021). Several lowland neotropical Russulaceae have affinities with tropical African species (Buyck, 1990; Buyck & Ovrebo, 2002; De Crop *et al*., 2017). Molecular divergence time estimates vary, however. A recent phylogenomic study (Looney *et al*., 2022) estimated a crown age of *c*. 60 Myr (uncertainty range 57–64 Myr) for the ECM Russulaceae, which places its diversification firmly after Gondwanan breakup. Prior estimates of Looney *et al*.(2016) and Varga *et al*.(2019) (58 and 83 Myr, respectively) also supported a post‐Gondwanan scenario. A much older estimate of 188 Myr (Sánchez‐García *et al*., 2020) appears to be an outlier (Fig. 1), pre‐dating even the radiation of the oldest ECM host plant lineage, Pinaceae, in the Late Jurassic (Tedersoo, 2017). Here, we assume that the ECM Russulaceae diversified when South America was already separated from Africa, and we reconstruct their biogeographic history in the Neotropics under this scenario.

To accomplish this, we generated a time‐calibrated phylogeny by supplementing a backbone tree of globally representative sequences with new data from a large, collaborative sampling effort focusing on the Neotropics. The resulting supertree was used to estimate range evolution and diversification. We tested the following hypotheses regarding neotropical Russulaceae:

1 Tropical South American lowlands host old lineages that are related to tropical African taxa, whereas the Andes and the adjacent Patagonia have younger lineages unrelated to tropical lowland taxa.
2 Dispersal into the Neotropics coincided with Palaeocene and Eocene boreotropical conditions and the closure of the Panama isthmus.
3 Tropical South America and with other tropical areas have a lower diversification rate than temperate areas.

## Materials and Methods

### Sample collection and sequencing

We aimed to generate a phylogenetic tree of Russulaceae that is globally representative and well sampled for the Neotropics. We collected sporocarps and ECM root tips, and obtained samples from dried fungarium specimens, from several neotropical and neighbouring countries: Belize, Brazil, British Virgin Islands, Chile, Colombia, Costa Rica, Ecuador, French Guiana, Guadeloupe, Guyana, Martinique, Panama, and Puerto Rico. Tissue samples were preserved in 2× cetyltrimethyl ammonium bromide solution. DNA was extracted using a Wizard Genomic DNA Purification kit (Promega). We were able to amplify the internal transcribed spacer (ITS) region from 241 specimens, using standard primers and protocols (Gardes & Bruns, 1993). In addition, partial large ribosomal subunit (LSU; primers CTB6 + TW14 (White *et al*., 1990), or LR0R + LR7 (Vilgalys & Hester, 1990)) and partial RNA polymerase II gene, second largest subunit (*rpb2*; primers fRPB2‐5F + bRPB2‐7cR (Liu *et al*., 1999)), were amplified for 222 and 43 samples, respectively. Amplicons were then Sanger‐sequenced (GATC Biotech, Konstanz, Germany, or Macrogen, Lille, France) and chromatograms edited in Geneious v.6 (Biomatters, Auckland, New Zealand).

### Internal transcribed spacer dataset assembly

To produce a tree that would include as much diversity of Russulaceae as possible, including undescribed taxa and environmental samples, we used an approach based on operational taxonomic units (OTUs) similar to that of Looney *et al*.(2016).

We downloaded all 35 944 ITS sequence records annotated as Russulaceae (as of August 2021) on the International Nucleotide Sequence Database Collaboration or the fungal sequence database UNITE (Kõljalg *et al*., 2013) using the plutoF web platform (https://plutof.ut.ee). Sequences annotated as ‘chimeric’ were excluded, and we added our 241 new ITS sequences. The ITS1–5.8S–ITS2 region was extracted from this set using ITSx v.1.1.3 (Bengtsson‐Palme *et al*., 2013), keeping only matches at least 50 bp long. Nineteen sequences associated with backbone tips (see later) that did not pass this filtering step were added back in. The resulting set of 29 479 sequences was then clustered into OTUs using Vsearch v.2.9.1 (Rognes *et al*., 2016) with the cluster representative being the longest sequence (‑‑cluster_fast option). We used a 97% identity threshold, which is probably conservative (i.e. lumping species) but appropriate for the macroevolutionary scale of our study. Sequences clustered into 3543 OTUs, and their representative sequences were used for subsequent analyses.

### Phylogenetics

A ‘supertree’ approach was used, estimating first a backbone phylogeny using conserved markers and then inserting backbone‐constrained, genus‐level phylogenies estimated from the ITS sequences. For the backbone, we first assembled a set of nuclear LSU (nrLSU) (28S), *rpb1* (RNA polymerase II gene, largest subunit), and *rpb2* sequences from 437 accessions of Russulaceae and seven outgroup Russulales, based on previous studies (Buyck *et al*., 2008, 2018, 2020; Verbeken *et al*., 2014; Looney *et al*., 2016; De Crop *et al*., 2017; Wang *et al*., 2018). We added 28 of the newly sequenced Russulaceae for which nrLSU and/or *rbp2* were amplified successfully. In the backbone accessions, 324 matched accessions in the ITS dataset and were used to constrain the genus phylogenies (see later). Sequences for each locus were aligned with Mafft/E‐Ins‐i v.7.407 (Katoh & Standley, 2013), end columns with > 90% missing data were trimmed, and alignments then concatenated. A maximum likelihood phylogeny was estimated using RAxML v.8 (Stamatakis, 2014), a GTR+Γ substitution model for each of the LSU, *rpb1*, and *rpb2* partitions, and 1000 rapid bootstrap searches. Transfer bootstrap expectation (TBE) was calculated in addition to traditional Felsenstein bootstrap support, which is sensitive to ‘rogue taxa’ in large datasets (Lemoine *et al*., 2018).

We then estimated phylogenies from the ITS dataset of representative OTU sequences. To improve alignment and tree estimation, we divided them into three genus datasets (combining the small *Multifurca* with its sister *Lactarius*). The ITS sequences were searched via Blastn (Altschul *et al*., 1990) against the ITS sequences associated with backbone tips to assign them to a genus dataset. Sequences with < 70% similarity to a backbone ITS sequence and only a single OTU member were discarded as likely chimeric or mislabelled as Russulaceae. Rapid alignments and trees were made with Mafft (automatic option) and FastTree v.2.1.10 (Price *et al*., 2010) for visual inspection. We removed a further three sequences that appeared as conspicuously long branches. The final set of ITS sequences per genus was then aligned (with suitable outgroup sequences from the other genera) using Mafft/E‐Ins‐i. Alignments were trimmed and RAxML trees inferred as described for the backbone, specifying ITS1, 5.8S, and ITS2 partitions; splits that had a TBE support of 0.7 or higher in the backbone tree were constrained in the tree search.

To generate a single Russulaceae supertree, we first time‐calibrated the backbone tree, with outgroups removed, using penalized likelihood in treePL v.1.0 (Smith & O'Meara, 2012). The smoothing parameter value was selected through random‐subsample‐and‐replicate cross‐validation for orders of magnitude between 10^−1^ and 10^3^. In the absence of known Russulaceae fossils, we fixed the crown age to 1. Subclade trees were time‐calibrated in the same manner (with a further 83 long‐branch tips removed) and then inserted into the backbone tree using the ‘bind.tree’ function in R, scaling branch lengths relative to the backbone crown age of the clade replaced. To provide an absolute timescale, we report ages obtained when multiplying branch lengths with the age estimate of Looney *et al*. (2022); that is, 60 Myr with a range of 57–64 Myr (Fig. 1). The final supertree contained 3285 tips, corresponding to OTUs identified in the ITS dataset and representing 29 167 ITS sequences.

For phylogenetic tree manipulation and plotting, we used the packages ape v.5.3 (Paradis & Schliep, 2019), geiger v.2.0.6.1 (Harmon *et al*., 2008), phytools v.0.6.60 (Revell, 2011), and plotrix v.3.7.5 (Lemon, 2006) in R v.3.6.1.

### Biogeographic areas

We compared the biogeographic histories of Russulaceae among parts of the Neotropics and adjacent regions that feature different ECM host plant assemblages (Tedersoo, 2017; Nouhra *et al*., 2019; Delgat *et al*., 2020) (Fig. 1a): (1) Central America/Caribbean, with both tropical‐origin ECM host plant lineages such as *Coccoloba* or Nyctaginaceae tribe Pisonieae and temperate‐origin Fagaceae, Juglandaceae, and Pinaceae. (2) The Andes, with the temperate‐derived *Alnus acuminata*, widespread in the central part of the range, and *Quercus humboldtii*, restricted to northern Colombia, and some lowland Nyctaginaceae that reach the montane Yungas (Geml *et al*., 2014). (3) Lowland tropical South America, with several distinct, tropical host plant groups in the Cistaceae, Fabaceae, Polygonaceae, Nyctaginaceae, Dipterocarpaceae (e.g. *Dicymbe*, *Aldina*, *Coccoloba*, *Guapira*, *Neea*, *Pakaraimaea*, *Pseudomonotes*). (4) Patagonia, with Nothofagaceae (Tedersoo, 2017). We defined these areas by merging the corresponding ecoregions of Morrone (2014, 2015), using the shapefiles of Löwenberg‐Neto (2014, 2015). The rest of the global range of Russulaceae was divided into the five broad regions Afrotropics, Australasia with Oceania, Indomalaya, Nearctic, and Palaearctic (Fig. 1a; Dinerstein *et al*., 2017).

Operational taxonomic units were assigned to one or more of these areas first by the country recorded for each ITS sequence clustering with the OTU, if this was unambiguous. We then used, in the following order, sampling coordinates, area descriptions geocoded with the ‘geocode_OSM’ R function, and information from the original literature associated to the record (for some of the remaining unassigned tips) to assign remaining sequences, using the R packages sf (Pebesma, 2018) and tmaptools (Tennekes, 2020). A total of 25 937 sequences (88.9% of those represented in the supertree) and 3153 OTUs (96%) could be assigned in this way (Table 1). We plotted the tip areas against the tree for visual inspection. In five instances, the placement (isolated tropical South American or Patagonian tip inside a north‐temperate clade) and the host and sampling metadata suggested introduced occurrences; these areas were ignored.

**Table 1:** Overview of internal transcribed spacer (ITS) sequences and operational taxonomic units (OTUs) by geographic area.

Area	ITS sequences		OTUs	
Focal				
Andes	46	0.2%	22	0.7%
Central America/Caribbean	514	1.8%	213	6.5%
Patagonia	16	0.1%	9	0.3%
Tropical S. America	303	1%	109	3.3%
Nonfocal				
Afrotropic	1055	3.6%	417	12.7%
Australasia and Oceania	445	1.5%	226	6.9%
Indomalayan	1585	5.4%	659	20.1%
Nearctic	4550	15.6%	737	22.4%
Palearctic	17 423	59.7%	1293	39.4%
Unassigned	3230	11.1%	132	4%
Total	29 167		3285	

The numbers shown are for sequences represented in the final Russulaceae supertree, after various filtering steps. Note that OTU numbers and percentages do not sum to 100% as one OTU can occur in several areas.

We summarized the overlap in OTUs between areas to assess recent dispersal and compared it with estimates of more ancient dispersal (see later).

### Biogeographic modelling

We estimated ancestral areas using a simple Markov model of trait evolution. The frequently used dispersal–extinction–cladogenesis model (Ree *et al*., 2005) proved too computationally expensive for our phylogeny and also has various issues (Ree & Sanmartín, 2018). We fitted a one‐parameter trait evolution model to the supertree and the tip areas assigned in the corhmm R package v.2.7 (Beaulieu *et al*., 2021), which can handle polymorphic tips and missing values.

With the estimated ancestral areas, we summarized lineages that have a relative likelihood of > 0.5 to occur in one of the four focal areas. We recovered their stem ages (divergence from groups outside the focal areas) and, if applicable, crown ages (first divergence within the focal areas). We then assessed, under the two ages scenarios, whether these ages coincide with the following biogeographic events: the split between South America and Antarctica, 50 Ma (van de Lagemaat *et al*., 2021); boreotropical conditions during the Palaeocene and Eocene (Wolfe, 1975); rapid north Andean uplift, 8–5 Ma (Pérez‐Escobar *et al*., 2022); and the Panama isthmus biotic interchange, beginning 20 Ma (O'Dea *et al*., 2016).

To count the number of dispersal events (i.e. area state changes) and infer dispersal rates *post hoc*, we stochastically mapped area evolution histories on the supertree 100 times using the ‘makeSimmap’ function in corhmm. From these stochastic maps, we obtained median values and 95% quantile ranges of dispersal counts between areas. We also summarized dispersal counts to and from each area over time. For this, we used 10 equal‐sized time windows since the origin of Russulaceae (representing 6 Myr windows under our calibration), which proved a reasonable compromise between detecting patterns in time vs uncertainty (i.e. the smaller the windows, the more uncertainty there is around the event counts).

### Diversification analysis

Diversification rates were estimated for the full tree using Bamm v.2.5 (Rabosky, 2014). Critique, especially of earlier versions of Bamm (Moore *et al*., 2016), has been addressed (Rabosky *et al*., 2017); Bamm was best suited to our purpose as we needed specific per‐branch rates for comparing diversification rates per area (see later). We ran Bamm for 200 million Markov chain Monte Carlo generations and sampling every 10 000^th^ generation. Effective sample sizes were ensured to be > 200 using the R package coda (Plummer *et al*., 2006), and the first 20% of posterior samples were discarded as burn‐in. Note that the conservative ITS clustering cut‐off likely underestimated the number of species, and thus the most recent diversification rates.

In the absence of a feasible method to jointly model diversification and range evolution (Goldberg *et al*., 2011) for such a large phylogeny and multiple areas, we partitioned per‐branch diversification rates by area in an approach similar to Chazot *et al*.(2021). We randomly paired the Bamm posterior samples with the area evolution stochastic maps. For each pair, the tree branches were divided into segments with the same range and diversification regime and no longer than 2% of the root height. We then calculated the mean and 95% credible interval of diversification rate per area, both overall and for each of 20 equal‐sized time windows. The R code for this is available in the online repository.

## Results

### Internal transcribed spacer sequences and occurrence in biogeographic areas

We compiled a dataset of 29 167 global ITS sequences of the ectomycorrhizal Russulaceae clade. These were obtained through a series of filtering steps applied to 35 944 sequences retrieved from public databases, combined with 241 new sequences generated for neotropical taxa. These sequences clustered into 3285 OTUs at a 97% similarity threshold, represented as tips in the Russulaceae supertree (Table 1), with 2234 belonging to *Russula*, 565 to *Lactarius*, 472 to *Lactifluus*, and 14 to *Multifurca*. Of these, 879 sequences and 353 OTUs could be assigned to one of four focal areas of the Neotropics and adjacent regions, with Central America/Caribbean having the highest numbers. Most sequences and OTUs in the dataset were assigned to the Palaearctic and Nearctic areas, respectively. The Palaearctic was overrepresented among sequences compared with OTUs (59.7% vs 39.4%), indicating a higher sampling depth in this area than in the others.

### Russulaceae phylogeny and divergence times

**Fig. 2:**
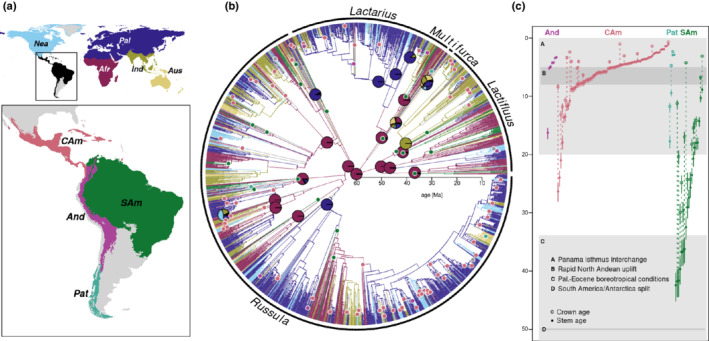
Ancestral range estimation and neotropical lineages of ectomycorrhizal Russulaceae (*Lactarius*, *Lactifluus*, *Multifurca* and *Russula*). (a) Defined areas, including the four focal areas (enlarged inset): Afr, Afrotropics; And, Andes; Aus, Australasia; CAm, Central America/Caribbean; Ind, Indomalaya; Nea, Nearctic; Pal, Palaearctic. (b) Dated supertree of the ectomycorrhizal Russulaceae with inferred ancestral ranges. The 3285 tips represent 29 167 internal transcribed spacer sequences clustered into operational taxonomic units. Ages are based on the previous estimate of 60 Myr (uncertainty range 47–64 Myr; Looney *et al*., 2022) for the crown age of the ectomycorrhizal Russulaceae. Ancestral ranges were estimated under a one‐parameter trait evolution model integrating over multiple‐state tips. Branches are coloured by the area with the highest relative likelihood as part of the inferred ancestral range at the child node. Relative likelihoods for ancestral areas are given as pie charts for genera, subgenera, and the Russulaceae crown node. Coloured dots indicate the stem branches of 110 lineages with an estimated origin in one of the focal areas (grey indicates that none of two or more focal areas has a relative likelihood of > 0.5). (c) Stem ages and crown ages (where applicable) of 110 lineages with an estimated origin in one of the focal areas. Grey polygons and horizontal lines indicate major biogeographic events in the region.

We inferred a 3285‐OTU Russulaceae supertree (Fig. 2b) based on ITS phylogenies constrained with a backbone phylogeny. The 444‐tip backbone phylogeny we inferred from LSU, *rpb1*, and *rpb2* data represents all named subgenera of the four Russulaceae genera (Supporting Information Fig. S1). Relationships among *Russula* subgenera were difficult to resolve in previous studies (Looney *et al*., 2016; Bazzicalupo *et al*., 2017; Buyck *et al*., 2018, 2020). We recovered low Felsenstein bootstrap, but high TBE, support among *Russula* subgenera and subg. *Heterophyllidia* as sister to the other subgenera. The backbone tree also suggests – based on a single LSU sequence – that the tropical South American *Russula campinensis* is sister to the rest of the genus and does not fall in any of the subgenera described. In *Lactarius*, the poorly defined subgenus *Russularia* is paraphyletic, and several unclassified lineages diverged earlier than the named subgenera, as found previously (Verbeken *et al*., 2014; Wisitrassameewong *et al*., 2016). The backbone notably supported an undescribed tropical South American species from Guyana (OTU KC155399) as sister to the remainder of *Lactarius*. Within *Lactifluus*, our backbone differed from relationships found previously (De Crop *et al*., 2017; Delgat *et al*., 2020) in finding TBE support for sister relationships between both subg. *Lactariopsis*/subg. *Pseudogymnocarpi* and subg. *Lactifluus*/subg. *Gymnocarpi*.

Under the age scenario of Looney *et al*.(2022), the estimated crown age of the largest genus *Russula* was *c*. 56 Myr (uncertainty range 53–59 Myr), followed by *Lactifluus* at 50 Myr (47–53 Myr), *Lactarius* at 42 Myr (40–45 Myr), and *Multifurca* at 34 Myr (32–36 Myr) (Fig. 2; see also the calibrated backbone in Fig. S1).

### Biogeographic origins and neotropical lineages

The Afrotropics were supported as the most likely ancestral area of Russulaceae in our biogeographic analysis (Fig. 2; see also all tips/OTUs detailed in Fig. S3). This was also the case for each of the three largest genera, *Lactarius*, *Lactifluus*, and *Russula*, with relative likelihoods of > 0.95 for the Afrotropics at these nodes. The earliest diverging clades at subgenus level in these three genera were also each estimated as Afrotropic in origin, except *Russula* subgenera *Russula* and *Crassotunicata* (Fig. S1a).

We found 110 distinct Russulaceae lineages occurring in the wider Neotropics, of which 76 are single‐OTU lineages and 34 clades with more than one OTU (Fig. 2b,c; Table 2; see also lineages numbered in Fig. S3). The oldest lineages are found in tropical South America, with the oldest stem age at *c*. 42 Myr (40–45 Myr), indicating arrival in the mid‐Eocene at the earliest. Some of these clades have spread to other areas: some Central American/Caribbean clades included tropical South America OTUs, and vice versa (e.g. nos. 53, 60, 66 and 73 in *Russula*, and nos. 106, 107 and 110 in *Lactifluus*; Fig. S3), whereas one tropical South American clade of *Lactifluus* (no. 106) included Australasian taxa. Estimated ages of these lineages allow us to assess whether they overlap with major biogeographic events that have shaped the regional biota (Fig. 2c).

**Table 2:** Overview of Russulaceae lineages in the Neotropics and adjacent regions.

Area	No. of lineages	Of which clades	Oldest crown ages (Myr)	Oldest stem age (Myr)
Andes	7	0	—	16.3 (15.4–17.3)
Central America/Caribbean	80	16	13.1 (12.5–14)	26.3 (25–28.1)
Patagonia	4	3	11.8 (11.2–12.5)	17.8 (16.9–18.9)
Tropical South America	19	15	29.9 (28.4–31.9)	42.4 (40.3–45.2)

These represent lineages that have an ancestral relative likelihood of > 0.5 to occur in the area but may also occur in other areas. Ages are based on the previous estimate of 60 Myr (uncertainty range 47–64 Myr; Looney *et al*., 2022) for the crown age of the ectomycorrhizal Russulaceae. Values in parentheses are age uncertainty ranges based on the 95% highest posterior density interval (57–64 Myr) of Looney *et al*. (2022).

**Fig. 3:**
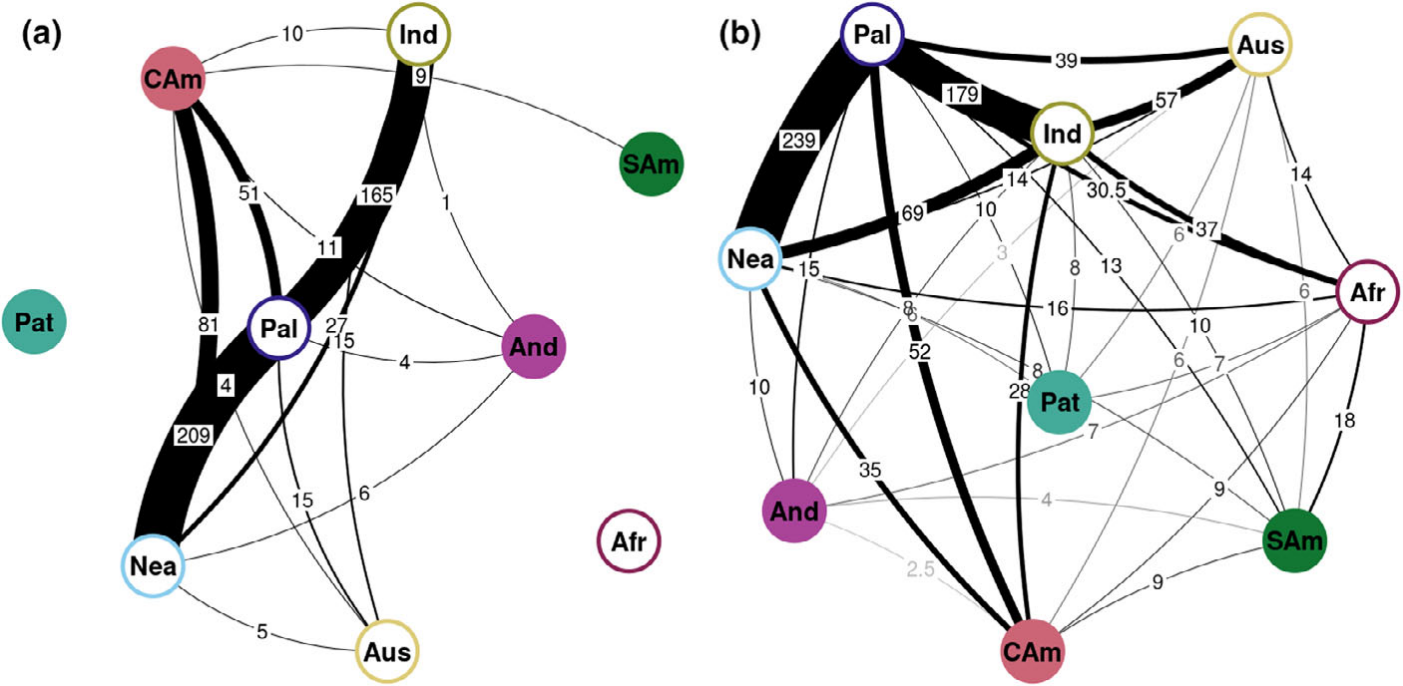
Connectivity between biogeographic areas in the ectomycorrhizal Russulaceae (*Lactarius*, *Lactifluus*, *Multifurca*, and *Russula*). (a) Overlap in operational taxonomic units (OTUs) between areas. Edge thickness is proportional to the number of shared OTUs. (b) Inferred numbers of dispersal events between areas. Edge thickness is proportional to the number of dispersals. Edges were only drawn for rates whose 95% quantile range excludes zero. The counts are mean numbers of dispersals inferred from stochastic mapping under the estimated dispersal/area transition rate.

The seven Andean lineages in *Russula*, *Lactarius*, and *Lactifluus* all appeared to have immigrated relatively recently, with no diversification *in situ*. The stem ages of all but one lineage (which appears as an old outlier) coincide with or are younger than the rapid north Andean uplift 5–8 Ma. These Andean lineages mainly emerge from Nearctic and/or Palaearctic clades, consistent with north‐to‐south migration. Only two Nyctaginaceae‐associated OTUs from the Yungas emerge from lowland tropical South American clades (*Lactifluus* UDB004277¦L6094 and *Russula* UDB004278¦L6090c; Fig. S3). There were also 12 Andean OTUs shared with other areas, mainly Central America/Caribbean and the Nearctic (Fig. 3a), suggesting very recent immigration into the Andes.

The four Patagonian lineages (*Russula*: nos. 7, 8 and 61; *Lactarius*: no. 94; Fig. S3c,r,ae) diverged at different times since *c*. 18 Ma and much more recently than the South America–Antarctica split. All four emerge from within or are sister to Australasian clades. Note that ITS sequences appear to be the only record of *Lactarius* associated with *Nothofagus* – based on sequence metadata – in the Patagonian region, as the genus was not listed in a previous overview (Barroetaveña *et al*., 2019). These and their closest relatives from Australasia represent a yet undescribed clade of *Lactarius*.

The 80 lineages from Central America/Caribbean occurred in *Russula*, *Lactarius*, and *Lactifluus*. Whereas *Multifurca* occurs in Central American oak forests (Montoya *et al*., 2003; Wang *et al*., 2018), no ITS sequence assignable to that area was available. None of the lineages is old enough to have diverged under Palaeocene boreotropical conditions. All but three ages are coincident with the increased biotic interchange across the Panama isthmus. The large majority of Central American/Caribbean lineages emerged from north‐temperate clades, whereas some occurred in clades with South American relatives.

Of the 19 lowland tropical South American lineages, several branched deeply at subgenus level. In *Russula*, a clade composed of *R. campinensis* (an unusual pleurotoid species from lowland tropical South America; Henkel *et al*., 2000) and three related OTUs branched at *c*. 33 Ma (no. 82, Fig. S3x). Together with OTUs from several other areas, this group is sister to the remainder of *Russula*. From Guyana, KC155399 with two sequences (no. 95) represents an undescribed lineage of *Lactarius* sister to the rest of the genus (Fig. S3a). Another well‐supported ancient lowland tropical South American clade of *Lactarius* resides in subg. *Plinthogalus* (no. 93, Fig. S3a,e). In *Lactifluus*, our tree places a lowland tropical South American clade with *Lactifluus ceraceus* and related OTUs (no. 105, Fig. S3a) sister to subg. *Lactifluus* and not in subg. *Pseudogymnocarpi* (Crous *et al*., 2017).

All lowland tropical South American lineages diverged well after the Africa–South America split. However, several of these old lineages were sister to clades with an estimated African origin (e.g. nos. 42, 62 and 82 in *Russula*; no. 95 in *Lactarius*; nos. 105, 106 and 101 in *Lactifluus*). Boreotropical migration can potentially account for these relationships in seven lineages that diverged in the Eocene. The other 12 lineages were too young for boreotropical migration. The youngest of these is a South American lineage clearly emerging from within an African clade (no. 63, *Russula puiggarii* and relatives) at *c*. 9 Ma, strongly suggesting direct dispersal across the Atlantic.

### Dispersal to and from the Neotropics

We summarized overlap in OTUs between areas, representing recent dispersal, and also estimated past dispersal events between them (Fig. 3). Two higher latitude areas, Nearctic and Palaearctic, have the highest OTU overlap (Fig. 3a) and the highest estimated dispersal count (Fig. 3b), followed by the Palaearctic and Indomalaya. Patagonia shared no OTU with any of the focal areas of the Neotropics. It, however, had possible ancient dispersal links with several other areas, which may reflect the uncertainty in simulating dispersal routes along branches, as plotting of ancestral areas strongly suggested the Patagonian lineages are related to Australasian lineages, as already noted herein. The Andes were most strongly linked with the Nearctic and Central America/Caribbean. Central America/Caribbean was most strongly linked with the Nearctic and Palaearctic. Lowland tropical South America only shared OTUs with Central America/Caribbean but had most dispersal links with the Afrotropics.

**Fig. 4:**
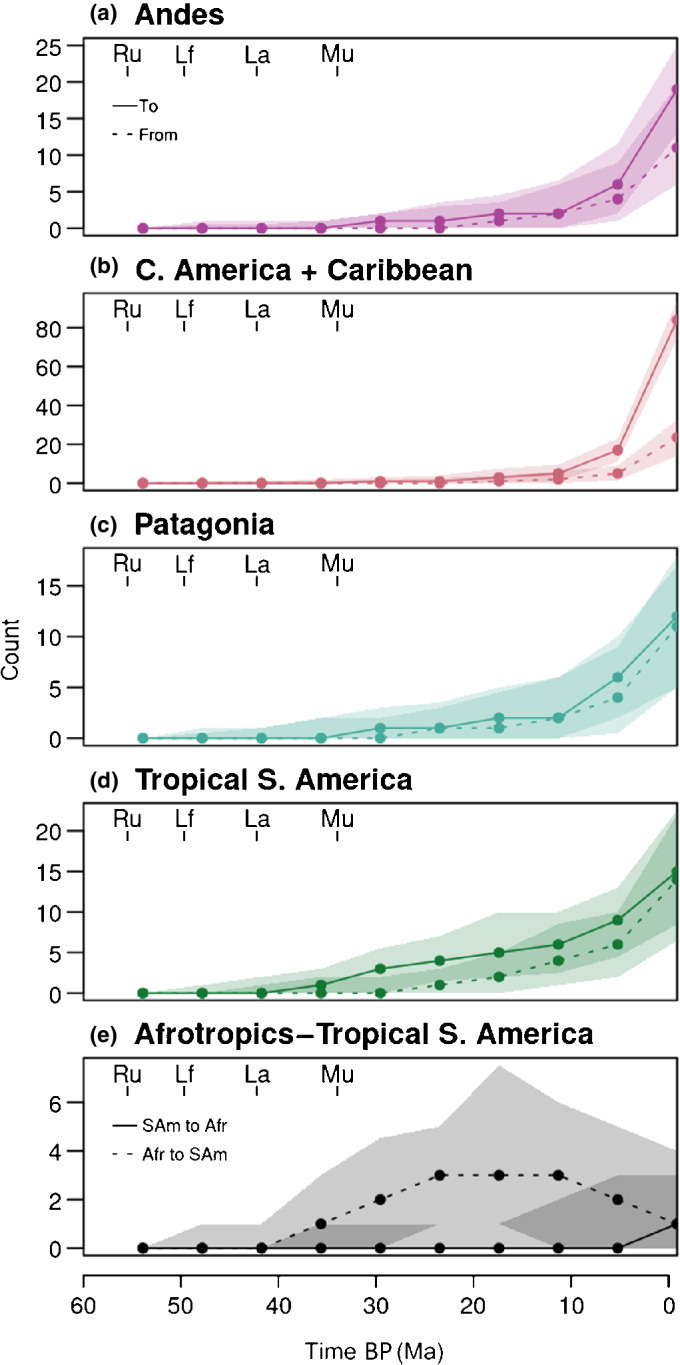
Dispersal to and from the Neotropics through time in the ectomycorrhizal Russulaceae (*Lactarius*, *Lactifluus*, *Multifurca* and *Russula*). The counts are median numbers of dispersals inferred from stochastic mapping for 10 equally spaced time periods between the root of Russulaceae and the present. Values are given for (a–d) the four focal areas and (e) specifically for dispersal between the Afrotropics and tropical South America. Shaded areas are 95% quantile ranges. Ages are based on the previous estimate of 60 Myr (uncertainty range 47–64 Myr; Looney *et al*., 2022) for the crown age of the ectomycorrhizal Russulaceae. Crown ages of the four genera are indicated for reference (Ru, *Russula*; Lf, *Lactifluus*; La, *Lactarius*; Mu, *Multifurca*). Ma, million years ago.

We also summarized Russulaceae immigration and emigration for the neotropical focal areas (Fig. 4). All areas showed an increase towards the present, reflecting their increasing number of extant lineages over time. In most areas, immigration and emigration were roughly equal across time periods, but a markedly stronger recent increase in immigration compared with emigration was seen in Central America/Caribbean. Dispersals to lowland tropical South America were mostly unidirectional from the Afrotropics, albeit with large uncertainty intervals (Fig. 4e).

### Diversification rates

**Fig. 5:**
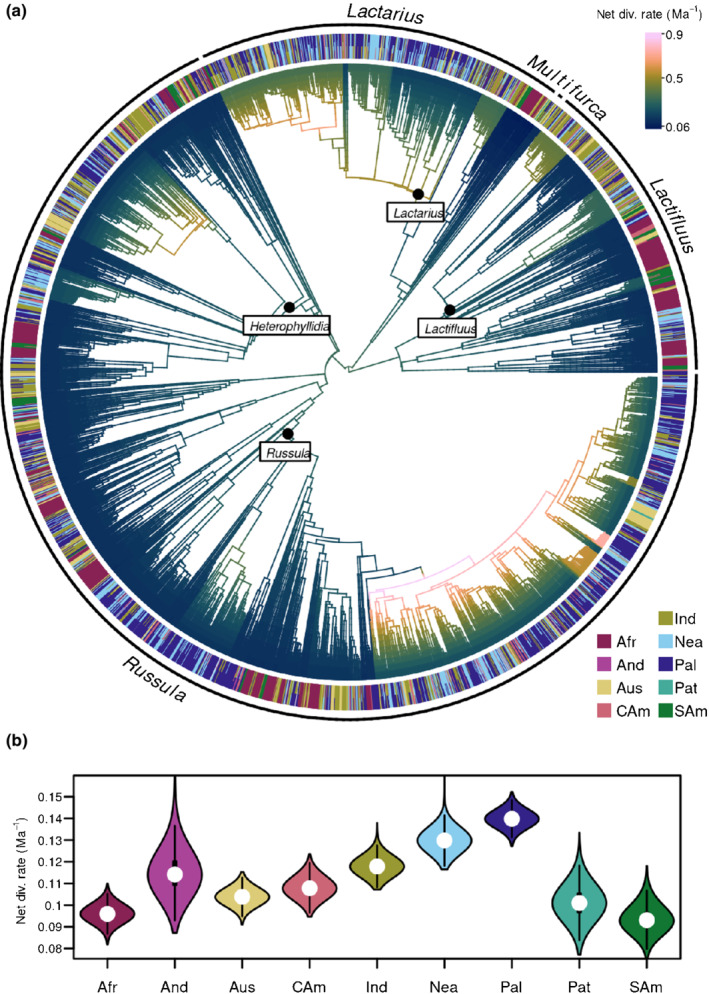
Net diversification across clades and areas in the ectomycorrhizal (ECM) Russulaceae (*Lactarius*, *Lactifluus*, *Multifurca*, and *Russula*). Diversification rates were inferred with Bamm v.2.5 (Rabosky, 2014). (a) Per‐branch net diversification rates averaged across posterior samples. The full 3385‐tip supertree is shown with the distribution of tips. Ages and rates are based on the previous estimate of 60 Myr (uncertainty range 47–64 Myr; Looney *et al*., 2022) for the crown age of the ECM Russulaceae. Subgenera containing clades with increased diversification rates are labelled. Ma, million years ago. (b) Net diversification rates per area. Violin plots are coloured by area; the white dots represent median values, and thick and thin vertical lines show 1× and 1.5× interquartile ranges, respectively. Diversification rates were averaged for branch segments in random pairs of Bamm posterior samples with stochastic ancestral area maps.

The estimated per‐branch net diversification rates ranged from *c*. 0.06 to 0.9 Myr^−1^ (Fig. 5a). There were rate increases in some clades, such as at the base and within *Lactarius* subg. *Lactarius*, in the crown clade of *Lactifluus* subg. *Lactifluus*, and within *Russula* subg. *Heterophyllidia*. *Russula* subg. *Russula* exhibited a rapid, mainly temperate crown diversification. Partitioning diversification rates by area showed that the Nearctic and Palaearctic had diversification rates on average *c*. 1.3–1.4 times higher than those of tropical South America, the Afrotropics, and Patagonia, with the Andes and Central America/Caribbean exhibiting intermediate values (Fig. 5b).

**Fig. 6:**
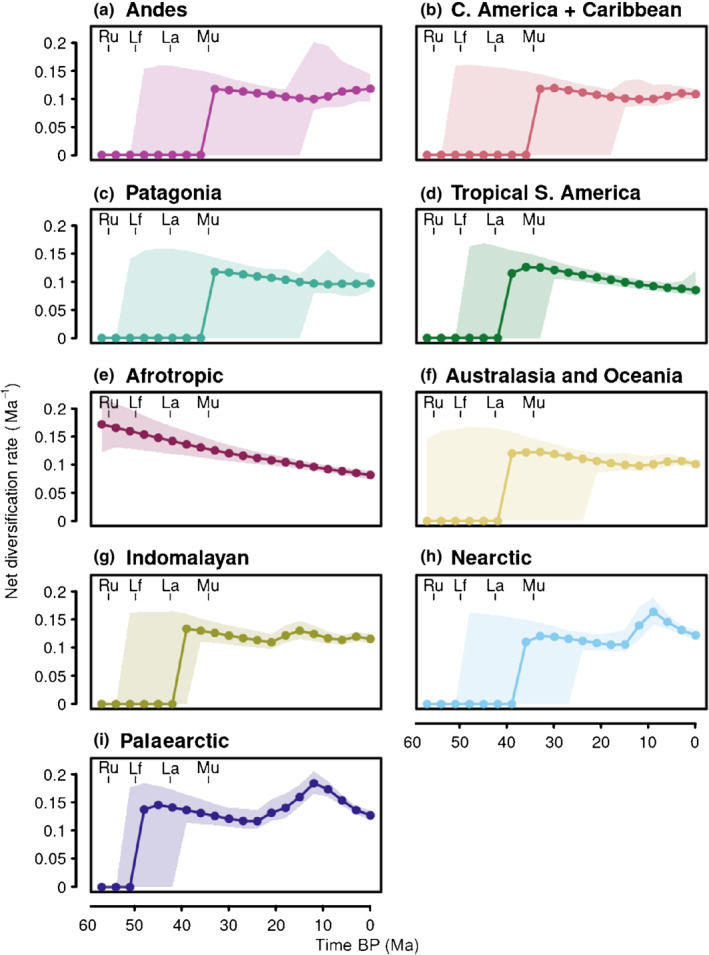
Diversification rates within areas through time in the ectomycorrhizal (ECM) Russulaceae (*Lactarius*, *Lactifluus*, *Multifurca* and *Russula*). Diversification rates were averaged for branch segments in random pairs of Bamm posterior samples with stochastic ancestral area maps, for 20 equally spaced time periods between the root of Russulaceae and the present. (a–i) Rates for the nine biogeographic areas defined, with the focal areas at the top (a–d). Shaded areas are 95% quantile ranges. Ages and rates are based on the previous estimate of 60 Myr (uncertainty range 47–64 Myr; Looney *et al*., 2022) for the crown age of the ECM Russulaceae. Crown ages of the four genera are indicated for reference (Ru, *Russula*; Lf, *Lactifluus*; La, *Lactarius*; Mu, *Multifurca*). Afr, Afrotropics; And, Andes; Aus, Australasia; CAm, Central America/Caribbean; Ind, Indomalaya; Nea, Nearctic; Pal, Palaearctic; Ma, million years ago.

Diversification rates plotted over time for the different areas are shown in Fig. 6. The putative area of origin for Russulaceae, the Afrotropics, showed a steady decline in diversification from initially high values at the crown diversification (Fig. 6e). By contrast, in the Nearctic and Palaearctic there were much more recent peaks at *c*. 9–12 Ma followed by a slowdown. Diversification also declined in tropical South America after the possible first emergence of lineages there at *c*. 35–40 Ma. In the Andes and Central America/Caribbean, there were slight recent upticks in diversification (Fig. 6a,b).

## Discussion

### A tropical African origin of ectomycorrhizal Russulaceae but fastest diversification in the temperate zone

We estimated that the speciose, globally distributed ECM clade in Russulaceae, as well its three largest genera, likely originated in tropical Africa (Fig. 1). An Afrotropical origin for Russulaceae had long been postulated (Pirozynski, 1983; Buyck *et al*., 1996) but a biogeographic analysis of the largest genus, *Russula* (Looney *et al*., 2016), suggested a temperate origin for that genus. This discrepancy for *Russula* may be explained by increased collection and sequencing having led to tropical taxa now being better represented in phylogenetic analyses (Buyck *et al*., 2018). Early diverging tropical lineages in *Russula* were revealed in our study, possibly resulting from our larger sampling of ITS sequences – 18 778 ITS sequences compared with 3348 in Looney *et al*.(2016). Unstable relationships among the major lineages of *Russula* were found in previous studies (Looney *et al*., 2016; Bazzicalupo *et al*., 2017; Buyck *et al*., 2018, 2020). The fact that we inferred African origins for most subgenera within *Russula*, however, supports an overall Africa origin of *Russula* even if the true relationships were different. Thus, results for both the largest genus, *Russula*, and the earliest diverging genus, *Lactifluus*, further support the overall African origin of Russulaceae.

Tropical origins have been inferred for several ECM fungal lineages (Matheny *et al*., 2009; Dentinger *et al*., 2010; Kennedy *et al*., 2012; Sánchez‐Ramírez *et al*., 2014; Han *et al*., 2018). In many of these groups, including Russulaceae, the greater species diversity in higher latitude regions therefore likely results from more recent, faster diversification relative to the Tropics. This is supported by our results of highest rates in the Nearctic and Palaearctic and the lowest in the Afrotropics and tropical South America (Fig. 5b), where it declined over time (Fig. 6). Most likely, the vast temperate and boreal forests dominated by ECM host lineages, such as Fagaceae and Pinaceae, offered opportunities for rapid diversification of lineages, as suggested previously (Bruns *et al*., 1998; Ryberg & Matheny, 2012; Looney *et al*., 2016). By contrast, the discontinuous, clustered host distribution in the Tropics would have offered less niche space. Our dispersal analyses corroborated this scenario, with both a high number of shared OTUs and dispersals between the north‐temperate regions (Fig. 3). The rapid increases of diversification rates we observed for Russulaceae in the Nearctic and Palaearctic indeed coincide with the spread of temperate vegetation after the Eocene thermal optimum (Zachos *et al*., 2001). Some of these north‐temperate lineages then migrated southward again into tropical latitudes, including Central America and the Andes (see later).

We suggest that ECM fungal diversity in tropical areas accumulated over a longer time period and more gradually than at higher latitudes. There are, however, also young clades of Russulaceae both in the Neotropics and Palaeotropics, and a simplistic terminology of ‘cradles’ vs ‘museums’ of biodiversity should probably be avoided (Vasconcelos *et al*., 2022). Further discoveries of fossil fungi, as well as ECM host plants, will hopefully refine the timeline of ECM fungal biogeography, especially in the Tropics.

### Lowland neotropical Russulaceae: ancient lineages, potential boreotropical migration, and evidence for transatlantic dispersal

Lowland tropical South America harbours several deep‐branching lineages within *Russula*, *Lactifluus*, and *Lactarius*, and most have affinities with tropical African lineages (Fig. 3b). Gondwanan vicariance is ruled out under the age scenario we considered. Splits between African and neotropical lineages are moreover asynchronous in time, making vicariance unlikely even if the very old age estimate of Sánchez‐García *et al*.(2020) were accepted. Migration across an Eocene boreotropical vegetation corridor, supposedly together with host plants, could explain some of the oldest Africa–South America divergences in Russulaceae. The ECM dipterocarp genus *Pseudomonotes* from northern South America diverged from its tropical African sister genus in the Eocene, so it could have co‐migrated during that time (Bansal *et al*., 2022). The monotypic *Pakaraimaea* is probably older, but its precise affinities and age are yet to be confirmed (Ashton *et al*., 2021). Both these ECM hosts are dominated by Russulaceae mycobionts in northern South America (Smith *et al*., 2013; Vasco‐Palacios *et al*., 2018).

Several of the lowland South American lineages with African ancestors must have dispersed across the Atlantic, as they diverged after boreotropical conditions. The peak of dispersal from Africa to South America occurred after *c*. 30 Ma (Fig. 4). Tropical transatlantic dispersals are well evidenced in some tropical plant lineages (Pennington & Dick, 2004; Hughes *et al*., 2013). The successful dispersal of ECM fungi across oceans seems unlikely, as spores must survive the long journey and encounter a suitable habitat with a compatible host tree (Kropp & Albee‐Scott, 2010; Horton *et al*., 2013). Various mechanisms of dispersal are debated, including by wind or birds (Caiafa *et al*., 2021), but also co‐dispersal of fungi and host plants (Golan & Pringle, 2017). Spore attachment to floating fruits has been suggested as a means of fungal co‐dispersal with propagules of ECM *Pisonia* (Kropp & Albee‐Scott, 2010) and *Coccoloba* (Séne *et al*., 2018). Rafting of entire trees with roots and soil could theoretically vector plants and their fungal symbionts (Golan & Pringle, 2017). Possible ECM host candidates for co‐dispersal from Africa to the Neotropics are *Aldina* and *Dicymbe*, which diverged from African ancestors in the Oligocene to Miocene, and thus after boreotropical conditions (Tedersoo, 2017). Extant species of these genera are major ECM host plants throughout the Guiana Shield and host a plethora of ECM fungi, including Russulaceae (Singer *et al*., 1983; Moyersoen, 1993; Smith *et al*., 2011; Vasco‐Palacios *et al*., 2018).

### North‐to‐south migration into the Neotropics

Most Russulaceae in the Andes have biogeographic affinities with north‐temperate lineages, distinct from those of lowland tropical South America (with the exception of Nyctaginaceae‐associated species in the lower Yungas). We also found more OTUs shared between the Andes and northern regions than OTUs unique to the Andes, indicating recent range expansion. This suggests co‐migration with *Alnus* and *Quercus* as they moved southward in the Pleistocene (Tedersoo, 2017). *Alnus acuminat*a is a widespread pioneer species in middle elevation zones from Mexico to the Andes (Wicaksono *et al*., 2017), and southward co‐migration of the ECM host species and its mycobionts has been suggested previously (Kennedy *et al*., 2011).

In Central America and the Caribbean, immigration from the north, at a time of generally increased biotic exchange, appears to be the dominant dispersal direction. This is coherent with the importance of north‐temperate hosts such as *Quercus* and *Pinus* in the region (Halling & Mueller, 2005; Tedersoo, 2017). Our data indeed show a strong recent increase in Russulaceae immigration vs emigration (Fig. 4b). However, South American Russulaceae lineages have also migrated northwards into the region. Delgat *et al*. (2020) previously showed that most *Lactifluus* species on Caribbean islands have South American affinities as opposed to north‐temperate‐derived Central American species (Delgat *et al*., 2020). The neotropical host lineages *Coccoloba* or Pisonieae probably moved northwards with their mycobionts before the Panama isthmus closure (Tedersoo, 2017). Clearly, the Central American/Caribbean region, which we defined very broadly here, has been a zone of exchange between north‐temperate and neotropical lineages, which must have involved dispersal pathways across the Caribbean islands.

### A distinct Patagonian ectomycorrhizal macromycota

The Patagonian macromycota has long been recognized as distinct (Singer, 1953; Trierveiler‐Pereira *et al*., 2014). Our results demonstrated that Patagonian Russulaceae have no affinity with either Andean or tropical lowland South American Russulaceae, despite the fact that the sole extant Patagonian host plant group Nothofagaceae extended considerably northward in Eocene South America (Jaramillo *et al*., 2006). Divergence times of Patagonian Russulaceae do not indicate a vicariant association with the South America–Antarctica split, unlike in the *Nothofagus*‐specific fungal parasite *Cyttaria* (Peterson *et al*., 2010). Even during the increasing glaciation of Antarctica, *Nothofagus* likely persisted in coastal areas until the Pleistocene (Poole & Cantrill, 2006) and provided geographic ‘stepping stones’ for bird‐vectored ECM fungal dispersal between South America and Australasia (Caiafa *et al*., 2021).

### Conclusions

Most biogeographic studies of the Neotropics have focused on plants or animals. Here, we explored the neotropical biogeography of one of the largest families of ECM fungi, the Russulaceae, drawing the following conclusions:

1 Tropical South American lowlands host several old lineages related to tropical African taxa, whereas the Andes and the adjacent Patagonia have younger, unrelated lineages.
2 Some lineages may have dispersed into lowland tropical South America during Palaeocene/Eocene boreotropical conditions. Several, however, immigrated more recently via transatlantic dispersal from tropical Africa. Most origins of Central American/Caribbean taxa coincide with general increased biotic interchange across the closing Panama isthmus.
3 Tropical South America and the Afrotropics have lower diversification rates than temperate areas, probably due to the discontinuous distribution of ECM hosts.

Our analysis demonstrates the need for more taxonomic and ecological work on neotropical ECM fungi. In the Russulaceae, several deep‐branching neotropical lineages remain to be described. Our coarse‐scale analysis needs to be followed by detailed regional work on host and environmental drivers of neotropical ECM fungal biogeography. Russulaceae have adapted to environments as contrasting as wet Amazonia (Singer & Araujo, 1979; Singer & Aguiar, 1986; Vasco‐Palacios *et al*., 2018) and the semi‐arid Caatinga (Sà *et al*., 2019), which we here all lumped under one geographic unit. Neotropical ECM fungi are phylogenetically diverse and may well perform unique and potentially irreplaceable ecological functions that need to be documented. This is particularly urgent in a time when neotropical forests are being destroyed at unprecedented levels (Gomes *et al*., 2019).

## Author contributions

JH and MR conceived the study. TWH, EDC, AV, P‐AM, BB, HS and MG helped refine the study design. JH, P‐AM, TWH, M‐AN, FW, RC, MS, AV‐P, SG, FC and MG collected material in the field. JH, MS, AV‐P, EL, FC and SM performed laboratory work. JH, MR and TWH wrote the manuscript. All authors commented and agreed on the manuscript.

## Supporting information


**Fig. S1** Russulaceae backbone phylogeny.

**Fig. S2** Russulaceae backbone phylogeny, time calibrated.

**Fig. S3** Detailed Russulaceae supertree.
Please note: Wiley Blackwell are not responsible for the content or functionality of any Supporting Information supplied by the authors. Any queries (other than missing material) should be directed to the *New Phytologist* Central Office.

## Data Availability

Data and intermediate results, as well as R scripts for dispersal and diversification analyses, are available in an open Zenodo repository (doi: 10.5281/zenodo.4727866).
