# Supplementary material for: Biogeographic history of a large clade of ectomycorrhizal fungi, the Russulaceae, in the Neotropics and adjacent regions
Source: New Phytol. 2022 Jul 30;236(2):698–713. doi: 10.1111/nph.18365 (PMC9795906; doi:10.1111/nph.18365)

New Phytologist Supporting Information

Article title: Biogeographic history of a large clade of ectomycorrhizal fungi, the Russulaceae, in the Neotropics and adjacent regions

Authors: Jan Hackel, Terry W. Henkel, Pierre-Arthur Moreau, Eske De Crop, Annemieke Verbeken, Mariana Sà, Bart Buyck, Maria-Alice Neves, Aída Vasco-Palacios, Felipe Wartchow, Heidy Schimann, Fabian Carriconde, Sigisfredo Garnica, Régis Courtecuisse, Monique Gardes, Sophie Manzi, Eliane Louisanna, Mélanie Roy

Article acceptance date: 23 June 2022

Figure S2. Russulaceae backbone phylogeny, time-calibrated

The maximum likelihood tree was time-calibrated using penalised likelihood. Ages are based on the previous estimate of 60 Ma (uncertainty range 47-64 Ma; Looney *et al.*, 2020) for the crown age of the ECM Russulaceae. Subgenera, according to recent taxonomic work (Verbeken *et al.*, 2014; De Crop *et al.*, 2017; Buyck *et al.*, 2018; Wang *et al.*, 2018; Buyck, 2020), are labelled.

References

Buyck B, Zoller S, Hofstetter V. 2018. Walking the thin line… ten years later: the dilemma of above- versus below-ground features to support phylogenies in the Russulaceae (Basidiomycota). *Fungal Diversity* 89: 267–292.

Buyck B, Wang X-H, Adamčíková K, Caboň M, Jančovičová S, Hofstetter V, Adamčík S. 2020. One step closer to unravelling the origin of *Russula*: subgenus *Glutinosae* subg. nov. *Mycosphere* 11: 285–304.

De Crop E, Nuytinck J, Van de Putte K, Wisitrassameewong K, Hackel J, Stubbe D, Hyde KD, Roy M, Halling RE, Moreau P-A, *et al.* 2017. A multi-gene phylogeny of *Lactifluus* (Basidiomycota, Russulales) translated into a new infrageneric classification of the genus. *Persoonia* 38: 58–80.

Looney B, Miyauchi S, Morin E, Drula E, Courty PE, Kohler A, Kuo A, LaButti K, Pangilinan J, Lipzen A, *et al.* 2022. Evolutionary transition to the ectomycorrhizal habit in the genomes of a hyper-diverse lineage of mushroom-forming fungi. *New Phytologist* 233: 2294–2309.

Verbeken A, Stubbe D, van de Putte K, Eberhardt U, Nuytinck J. 2014. Tales of the unexpected: angiocarpous representatives of the Russulaceae in tropical South East Asia. *Persoonia* 32: 13–24.

Wang X-H, Halling RE, Hofstetter V, Lebel T, Buyck B. 2018. Phylogeny, biogeography and taxonomic re-assessment of *Multifurca* (Russulaceae, Russulales) using three-locus data. *PLOS ONE* 13: e0205840.

Fig. S2: Russulaceae backbone tree, time−calibrated

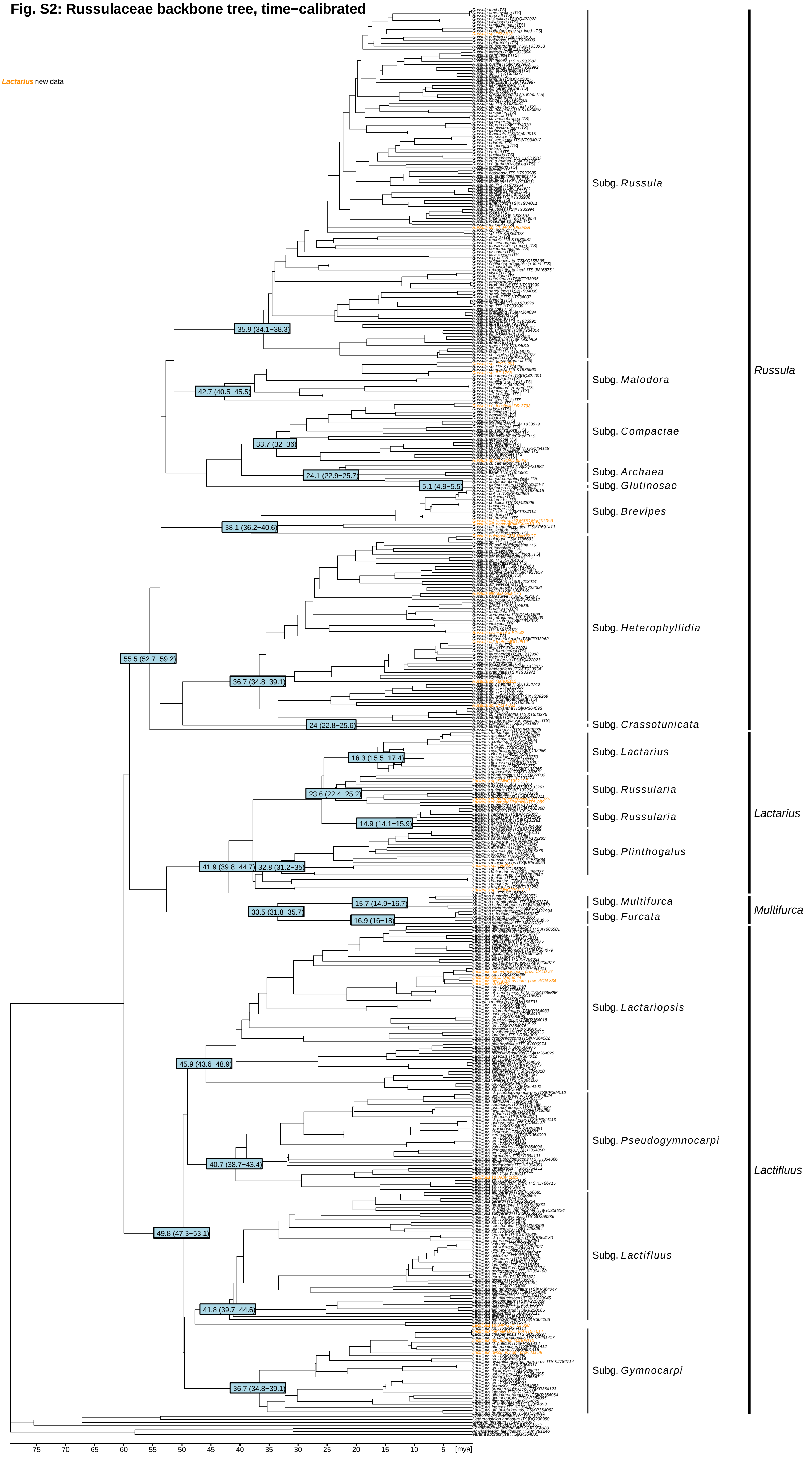

Supplement: Supplementary file 2 — Fig. S2 Russulaceae backbone phylogeny, time calibrated. [file NPH-236-698-s003.pdf]
