# Supplementary material for: Biogeographic history of a large clade of ectomycorrhizal fungi, the Russulaceae, in the Neotropics and adjacent regions
Source: New Phytol. 2022 Jul 30;236(2):698–713. doi: 10.1111/nph.18365 (PMC9795906; doi:10.1111/nph.18365)

New Phytologist Supporting Information

Article title: Biogeographic history of a large clade of ectomycorrhizal fungi, the Russulaceae, in the Neotropics and adjacent regions

Authors: Jan Hackel, Terry W. Henkel, Pierre-Arthur Moreau, Eske De Crop, Annemieke Verbeken, Mariana Sà, Bart Buyck, Maria-Alice Neves, Aída Vasco-Palacios, Felipe Wartchow, Heidy Schimann, Fabian Carriconde, Sigisfredo Garnica, Régis Courtecuisse, Monique Gardes, Sophie Manzi, Eliane Louisanna, Mélanie Roy

Article acceptance date: 23 June 2022

Figure S3. Detailed Russulaceae supertree

Complete 3,285-tip, time-calibrated supertree, based on ITS trees inferred for three subclades (*Lactifluus*, *Lactarius–Multifurca*, *Russula*) and inserted into the backbone tree. Areas assigned to tips, node support and relative ancestral range probabilities at nodes are shown. (a) Overview tree, showing subdivision into smaller plots for subclades (b-aj). Ages are based on the previous estimate of 60 Ma (uncertainty range 47-64 Ma; Looney *et al.*, 2020) for the crown age of the ECM Russulaceae. Tip labels give the accession number of the representative ITS sequence per OTU used for inferring ITS trees. Note that the taxon names are as per the original sequence metadata; they may be wrong or reflect outdated nomenclature.

References

Looney B, Miyauchi S, Morin E, Drula E, Courty PE, Kohler A, Kuo A, LaButti K, Pangilinan J, Lipzen A, *et al.* 2022. Evolutionary transition to the ectomycorrhizal habit in the genomes of a hyper-diverse lineage of mushroom-forming fungi. *New Phytologist* 233: 2294–2309.

Figure S3 a

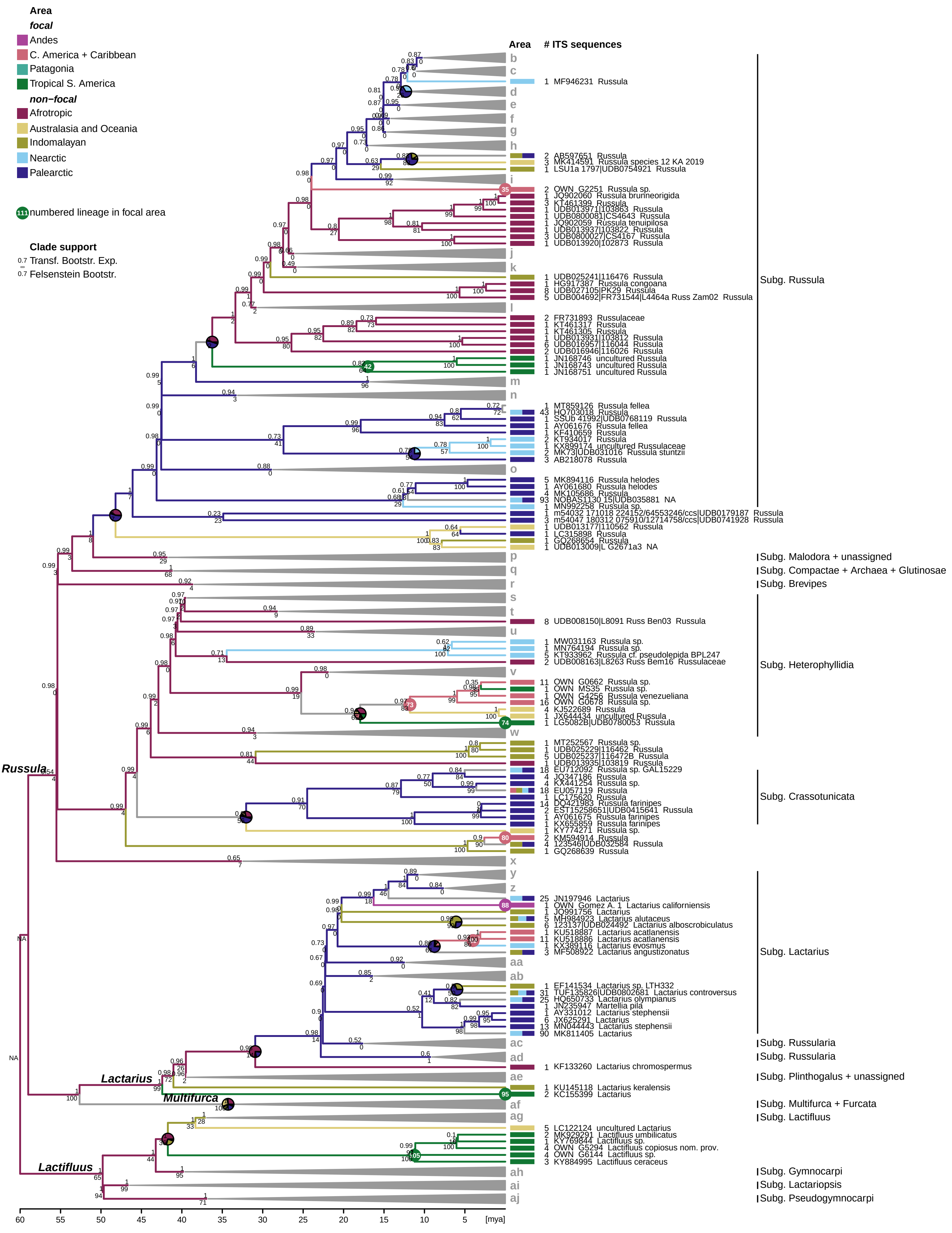

Area
focal
Andes
C. America + Caribbean
Patagonia
Tropical S. America
non−focal
Afrotropic
Australasia and Oceania
Indomalayan
Nearctic
Palearctic
numbered lineage in focal area
Clade support
Transf. Bootstr. Exp.
Felsenstein Bootstr.
Area
ITS sequences
1 MF946231 Russula
2 AB597651 Russula
3 MK414591 Russula species 12 KA 2019
1 LSU1a 1797|UDB0754921 Russula
2 OWN G2251 Russula sp.
1 JQ902060 Russula brunneorigida
3 KT461399 Russula
1 UDB013971|103863 Russula
1 UDB080008|CS4643 Russula
1 JQ902059 Russula tenuipilosa
1 UDB013937|103822 Russula
3 UDB080027|CS4167 Russula
1 UDB013920|102873 Russula
1 UDB025241|116476 Russula
1 HG917387 Russula congoana
8 UDB027105|PK29 Russula
5 UDB004692|FR731544|L4464a Russ Zam02 Russula
2 FR731893 Russulaceae
1 KT461317 Russula
1 KT461305 Russula
1 UDB013931|103812 Russula
6 UDB016957|116044 Russula
2 UDB016946|116026 Russula
1 JN168746 uncultured Russula
1 JN168743 uncultured Russula
1 JN168751 uncultured Russula
1 MT859126 Russula fellea
43 HQ703018 Russula
1 SSUb 41992|UDB0768119 Russula
1 AY061676 Russula fellea
1 KF410659 Russula
2 KT934017 Russula
1 KX899174 uncultured Russulaceae
2 MK73|UDB031016 Russula stuntzii
3 AB218078 Russula
5 MK894116 Russula helodes
1 AY061680 Russula helodes
4 MK105686 Russula
93 NOBAS1130 15|UDB035881 NA
1 MN992258 Russula sp.
1 m54032 171018 224152/64553246/ccs|UDB0179187 Russula
3 m54047 180312 075910/12714758/ccs|UDB0741928 Russula
1 UDB013177|110562 Russula
1 LC315898 Russula
1 GQ268654 Russula
1 UDB013009|L G2671a3 NA
8 UDB008150|L8091 Russ Ben03 Russula
1 MW031163 Russula sp.
1 MN764194 Russula sp.
5 KT933962 Russula cf. pseudolepida BPL247
2 UDB008163|L8263 Russ Bem16 Russulaceae
11 OWN G0662 Russula sp.
1 OWN MS35 Russula sp.
1 OWN G4256 Russula venezueliana
16 OWN G0678 Russula sp.
4 KJ522689 Russula
1 JX644434 uncultured Russula
1 LG5082B|UDB0780053 Russula
1 MT252567 Russula sp.
1 UDB025229|116462 Russula
5 UDB025237|116472B Russula
1 UDB013935|103819 Russula
18 EU712092 Russula sp. GAL15229
4 JQ347186 Russula
4 KX441254 Russula sp.
18 EU057119 Russula
1 LC175620 Russula
14 DQ421983 Russula farinipes
2 EST15258651|UDB0415641 Russula
1 AY061675 Russula farinipes
1 KX655859 Russula farinipes
1 KY774271 Russula sp.
2 KM594914 Russula
4 123546|UDB032584 Russula
1 GQ268639 Russula
25 JN197946 Lactarius
1 OWN Gomez A. 1 Lactarius californiensis
1 JQ991756 Lactarius
5 MH984923 Lactarius alutaceus
6 123137|UDB024492 Lactarius albocrobiculatus
1 KU518887 Lactarius acatlanensis
11 KU518886 Lactarius acatlanensis
1 KX389116 Lactarius evosmus
3 MF508922 Lactarius angustizonatus
1 EF141534 Lactarius sp. LTH332
31 TUF135826|UDB0802681 Lactarius controversus
25 HQ650733 Lactarius olympianus
1 JN235947 Martellia pila
1 AY331012 Lactarius stephensii
6 JX625291 Lactarius
13 MN044443 Lactarius stephensii
90 MK811405 Lactarius
1 KF133260 Lactarius chromospermus
1 KU145118 Lactarius keralensis
2 KC155399 Lactarius
5 LC122124 uncultured Lactarius
2 MK929291 Lactifluus umbilicatus
1 KY769844 Lactifluus sp.
4 OWN G5294 Lactifluus copiosus nom. prov.
4 OWN G6144 Lactifluus sp.
3 KY884995 Lactifluus ceraceus
b
c
d
e
f
g
h
i
j
k
l
m
n
o
p
q
r
s
t
u
v
w
x
y
z
aa
ab
ac
ad
ae
af
ag
ah
ai
aj
Subg. Russula
Subg. Malodora + unassigned
Subg. Compactae + Archaea + Glutinosae
Subg. Brevipes
Subg. Heterophyllidia
Subg. Crassotunicata
Subg. Lactarius
Subg. Russularia
Subg. Russularia
Subg. Plinthogalus + unassigned
Subg. Multifurca + Furcata
Subg. Lactifluus
Subg. Gymnocarpi
Subg. Lactariopsis
Subg. Pseudogymnocarpi
Russula
Lactarius
Multifurca
Lactifluus
60
55
50
45
40
35
30
25
20
15
10
5
[mya]

Figure S3 b

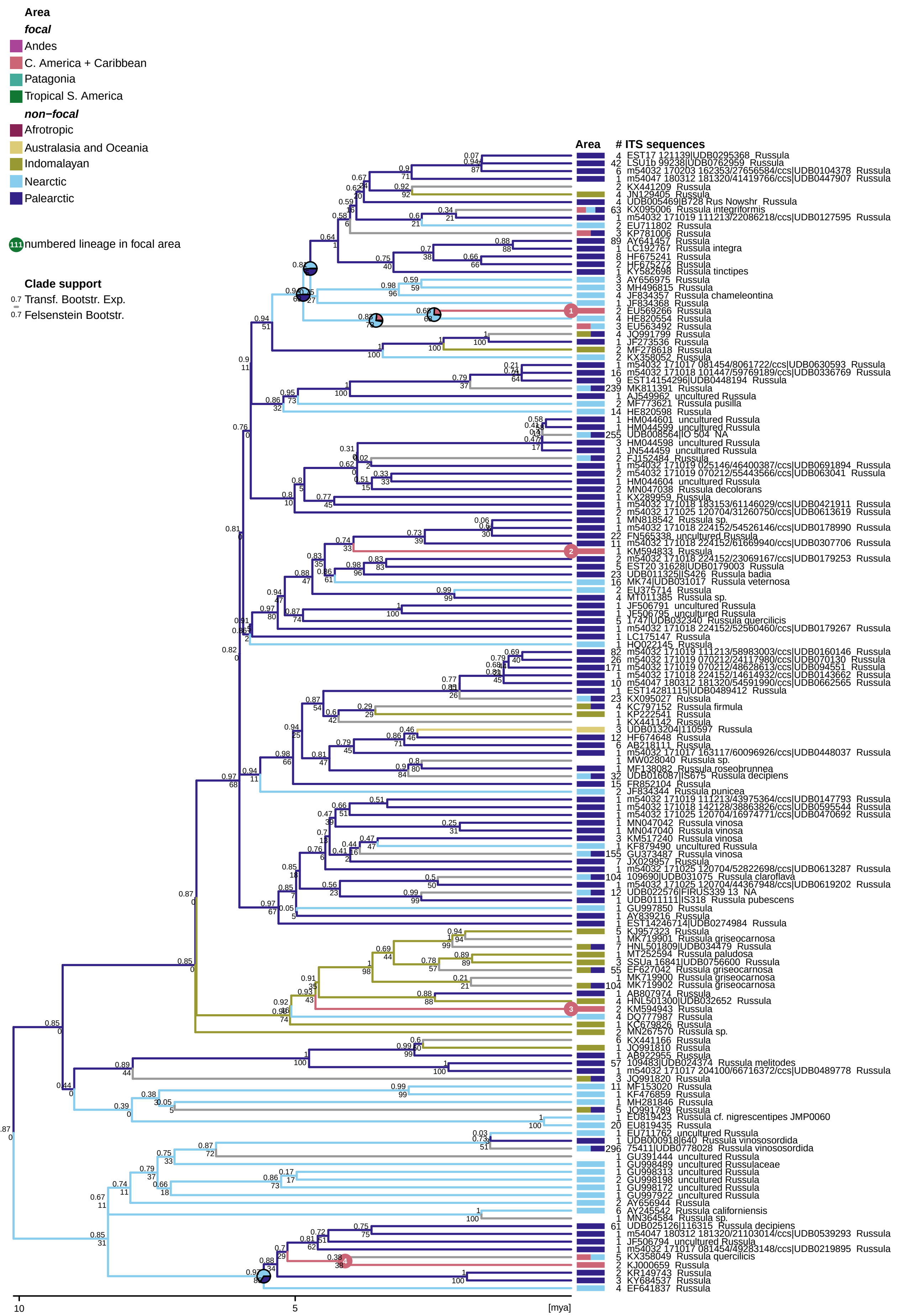

Area
focal
Andes
C. America + Caribbean
Patagonia
Tropical S. America
non−focal
Afrotropic
Australasia and Oceania
Indomalayan
Nearctic
Palearctic
111 numbered lineage in focal area
Clade support
0.7 Transf. Bootstr. Exp.
0.7 Felsenstein Bootstr.
Area
ITS sequences
4 EST17 121139|UDB0295368 Russula
42 LSU1b 99238|UDB0762959 Russula
6 m54032 170203 162353/27656584/ccs|UDB0104378 Russula
1 m54047 180312 181320/41419766/ccs|UDB0447907 Russula
2 KX441209 Russula
4 JN129405 Russula
4 UDB005469|B728 Rus Nowshr Russula
63 KX095006 Russula integriformis
1 m54032 171019 111213/22086218/ccs|UDB0127595 Russula
2 EU711802 Russula
3 KP781006 Russula
89 AY641457 Russula
1 LC192767 Russula integra
8 HF675241 Russula
2 HF675272 Russula
1 KY582698 Russula tinctipes
3 AY656975 Russula
3 MH496815 Russula
4 JF834357 Russula chameleontina
1 JF834368 Russula
2 EU569266 Russula
4 HE820554 Russula
3 EU563492 Russula
4 JQ991799 Russula
1 JF273536 Russula
2 MF278618 Russula
2 KX358052 Russula
1 m54032 171017 081454/8061722/ccs|UDB0630593 Russula
16 m54032 171018 101447/59769189/ccs|UDB0336769 Russula
9 EST14154296|UDB0448194 Russula
239 MK811391 Russula
1 AJ549962 uncultured Russula
2 MF773621 Russula pusilla
14 HE820598 Russula
1 HM044601 uncultured Russula
1 HM044599 uncultured Russula
255 UDB008564|IO 504 NA
3 HM044598 uncultured Russula
1 JN544459 uncultured Russula
2 FJ152484 Russula
1 m54032 171019 025146/46400387/ccs|UDB0691894 Russula
2 m54032 171019 070212/55443566/ccs|UDB063041 Russula
1 HM044604 uncultured Russula
2 MN047038 Russula decolorans
1 KX289959 Russula
1 m54032 171018 183153/61146029/ccs|UDB0421911 Russula
2 m54032 171025 120704/31260750/ccs|UDB0613619 Russula
1 MN818542 Russula sp.
1 m54032 171018 224152/54526146/ccs|UDB0178990 Russula
22 FN565338 uncultured Russula
11 m54032 171018 224152/61669940/ccs|UDB0307706 Russula
1 KM594833 Russula
2 m54032 171018 224152/23069167/ccs|UDB0179253 Russula
5 EST20 31628|UDB0179003 Russula
23 UDB011325|IS426 Russula badia
16 MK74|UDB031017 Russula veternosa
2 EU375714 Russula
4 MT011385 Russula sp.
1 JF506791 uncultured Russula
1 JF506795 uncultured Russula
5 1747|UDB032340 Russula quercilicis
1 m54032 171018 224152/52560460/ccs|UDB0179267 Russula
1 LC175147 Russula
1 HQ022145 Russula
82 m54032 171019 111213/58983003/ccs|UDB0160146 Russula
26 m54032 171019 070212/24117980/ccs|UDB070130 Russula
171 m54032 171019 070212/48628613/ccs|UDB094551 Russula
1 m54032 171018 224152/14614932/ccs|UDB0143662 Russula
10 m54047 180312 181320/54591990/ccs|UDB0662565 Russula
1 EST14281115|UDB0489412 Russula
23 KX095027 Russula
4 KC797152 Russula firmula
1 KP222541 Russula
1 KX441142 Russula
3 UDB013204|110597 Russula
12 HF674648 Russula
6 AB218111 Russula
1 m54032 171017 183117/60096926/ccs|UDB0448037 Russula
1 MW028040 Russula sp.
1 MF138082 Russula roseobrunnea
32 UDB016087|IS675 Russula decipiens
15 FR852104 Russula
2 JF834344 Russula punicea
1 m54032 171019 111213/43975364/ccs|UDB0147793 Russula
1 m54032 171018 142128/38863826/ccs|UDB0595544 Russula
1 m54032 171025 120704/16974771/ccs|UDB0470692 Russula
1 MN047042 Russula vinosa
1 MN047040 Russula vinosa
3 KM517240 Russula vinosa
1 KF879490 uncultured Russula
155 GU373487 Russula vinosa
7 JX029957 Russula
1 m54032 171025 120704/52822698/ccs|UDB0613287 Russula
104 109690|UDB031075 Russula claroflava
1 m54032 171025 120704/44367948/ccs|UDB0619202 Russula
12 UDB022576|FIRUS339 13 NA
1 UDB011111|IS318 Russula pubescens
1 GU997850 Russula
1 AY839216 Russula
1 EST14246771|UDB0274984 Russula
5 KJ957323 Russula
1 MK719901 Russula griseocarnosa
7 HNL501809|UDB034479 Russula
1 MT252594 Russula paludosa
3 SSUa 16841|UDB0756600 Russula
55 EF627042 Russula griseocarnosa
1 MK719900 Russula griseocarnosa
104 MK719902 Russula griseocarnosa
1 AB807974 Russula
4 HNL501300|UDB032652 Russula
2 KM594943 Russula
4 DQ777987 Russula
1 KC679826 Russula
2 MN267570 Russula sp.
6 KX441166 Russula
1 JQ991810 Russula
1 AB922955 Russula
57 109483|UDB024374 Russula melitodes
1 m54032 171017 204100/66716372/ccs|UDB0489778 Russula
3 JQ991820 Russula
11 MF153020 Russula
1 KF476859 Russula
1 MH281846 Russula
5 JQ991789 Russula
1 EU819423 Russula cf. nigrescentipes JMP0060
20 EU819435 Russula
1 EU711762 uncultured Russula
1 UDB000918|640 Russula vinososordida
296 75411|UDB0778028 Russula vinososordida
1 GU391444 uncultured Russula
1 GU998489 uncultured Russulaceae
1 GU998313 uncultured Russula
2 GU998198 uncultured Russula
1 GU998172 uncultured Russula
1 GU997922 uncultured Russula
2 AY656944 Russula
6 AY245542 Russula californiensis
1 MN364584 Russula sp.
61 UDB025126|116315 Russula decipiens
1 m54047 180312 181320/21103014/ccs|UDB0539293 Russula
1 JF506794 uncultured Russula
1 m54032 171017 081454/49283148/ccs|UDB0219895 Russula
5 KX358049 Russula quercilicis
2 KJ000659 Russula
2 KR149743 Russula
3 KY684537 Russula
4 EF641837 Russula
10
5
[mya]

Figure S3 c

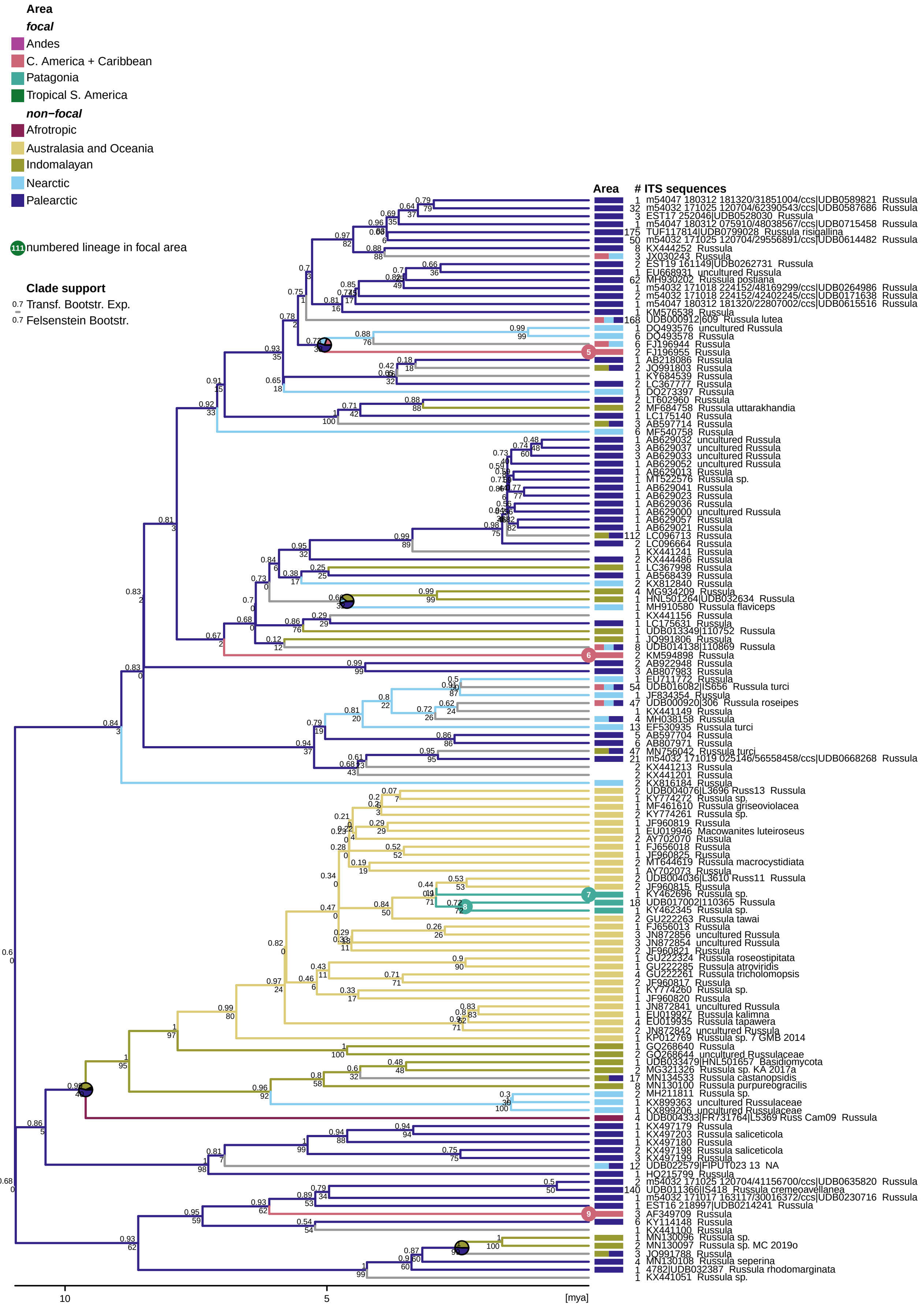

Area
focal
Andes
C. America + Caribbean
Patagonia
Tropical S. America
non−focal
Afrotropic
Australasia and Oceania
Indomalayan
Nearctic
Palearctic
111 numbered lineage in focal area
Clade support
0.7 Transf. Bootstr. Exp.
0.7 Felsenstein Bootstr.
Area
ITS sequences
1 m54047 180312 181320/31851004/ccs|UDB0589821 Russula
32 m54032 171025 120704/62390543/ccs|UDB0587686 Russula
3 EST17 252046|UDB0528030 Russula
1 m54047 180312 075910/48038567/ccs|UDB0715458 Russula
175 TUF117814|UDB0799028 Russula risigallina
50 m54032 171025 120704/29556891/ccs|UDB0614482 Russula
8 KX444252 Russula
3 JX030243 Russula
2 EST19 161149|UDB0262731 Russula
1 EU668931 uncultured Russula
62 MH930202 Russula postiana
1 m54032 171018 224152/48169299/ccs|UDB0264986 Russula
2 m54032 171018 224152/42402245/ccs|UDB0171638 Russula
1 m54047 180312 181320/22807002/ccs|UDB0615516 Russula
1 KM576538 Russula
168 UDB000912|609 Russula lutea
1 DQ493576 uncultured Russula
6 DQ493578 Russula
6 FJ196944 Russula
2 FJ196955 Russula
1 AB218086 Russula
2 JQ991803 Russula
1 KY684539 Russula
2 LC367777 Russula
1 DQ273397 Russula
2 LT602960 Russula
2 MF684758 Russula uttarakhandia
1 LC175140 Russula
3 AB597714 Russula
6 MF540758 Russula
1 AB629032 uncultured Russula
3 AB629037 uncultured Russula
3 AB629033 uncultured Russula
1 AB629052 uncultured Russula
1 AB629013 Russula
1 MT522576 Russula sp.
1 AB629041 Russula
1 AB629023 Russula
1 AB629036 Russula
1 AB629000 uncultured Russula
1 AB629057 Russula
1 AB629021 Russula
112 LC096713 Russula
2 LC096664 Russula
1 KX441241 Russula
2 KX444486 Russula
1 LC367998 Russula
1 AB568439 Russula
2 KX812840 Russula
4 MG934209 Russula
1 HNL501264|UDB032634 Russula
1 MH910580 Russula flaviceps
1 KX441156 Russula
1 LC175631 Russula
1 UDB013349|110752 Russula
1 JQ991806 Russula
8 UDB014138|110869 Russula
2 KM594898 Russula
2 AB922948 Russula
3 AB807983 Russula
1 EU711772 Russula
54 UDB016082|IS656 Russula turci
1 JF834354 Russula
47 UDB000920|306 Russula roseipes
1 KX441149 Russula
4 MH038158 Russula
13 EF530935 Russula turci
5 AB597704 Russula
6 AB807971 Russula
47 MN756042 Russula turci
21 m54032 171019 025146/56558458/ccs|UDB0668268 Russula
2 KX441213 Russula
2 KX441201 Russula
2 KX816184 Russula
2 UDB004076|L3696 Russ13 Russula
1 KY774272 Russula sp.
1 MF461610 Russula griseoviolacea
2 KY774261 Russula sp.
1 JF960819 Russula
1 EU019946 Macowanites luteiroseus
2 AY702070 Russula
1 FJ656018 Russula
1 JF960825 Russula
2 MT644619 Russula macrocystidiata
1 AY702073 Russula
2 UDB004036|L3610 Russ11 Russula
2 JF960815 Russula
1 KY462696 Russula sp.
18 UDB017002|110365 Russula
1 KY462345 Russula sp.
2 GU222263 Russula tawai
1 FJ656013 Russula
3 JN872856 uncultured Russula
3 JN872854 uncultured Russula
2 JF960821 Russula
1 GU222324 Russula roseostipitata
1 GU222285 Russula atroviridis
4 GU222261 Russula tricholomopsis
2 JF960817 Russula
1 KY774260 Russula sp.
1 JF960820 Russula
1 JN872841 uncultured Russula
1 EU019927 Russula kalimna
4 EU019935 Russula tapawera
2 JN872842 uncultured Russula
1 KP012769 Russula sp. 7 GMB 2014
1 GQ268640 Russula
2 GQ268644 uncultured Russulaceae
1 UDB033479|HNL501657 Basidiomycota
2 MG321326 Russula sp. KA 2017a
17 MN134533 Russula castanopsidis
8 MN130100 Russula purpureogracilis
2 MH211811 Russula sp.
1 KX899363 uncultured Russulaceae
1 KX899206 uncultured Russulaceae
4 UDB004333|FR731764|L5369 Russ Cam09 Russula
1 KX497179 Russula
1 KX497203 Russula saliceticola
1 KX497180 Russula
2 KX497198 Russula saliceticola
3 KX497199 Russula
12 UDB022579|FIPUT023 13 NA
1 HQ215799 Russula
2 m54032 171025 120704/41156700/ccs|UDB0635820 Russula
140 UDB011366|IS418 Russula cremeoavellanea
1 m54032 171017 163117/30016372/ccs|UDB0230716 Russula
1 EST16 218997|UDB0214241 Russula
3 AF349709 Russula
6 KY114148 Russula
1 KX441100 Russula
1 MN130096 Russula sp.
2 MN130097 Russula sp. MC 2019o
3 JQ991788 Russula
4 MN130108 Russula seperina
1 4782|UDB032387 Russula rhodomarginata
1 KX441051 Russula sp.
5
6
7
9
10
5
[mya]

Figure S3 d

Area

focal

- Andes
- C. America + Caribbean
- Patagonia
- Tropical S. America

non−focal

- Afrotropic
- Australasia and Oceania
- Indomalayan
- Nearctic
- Palearctic

111 numbered lineage in focal area

Clade support

0.7 Transf. Bootstr. Exp.

0.7 Felsenstein Bootstr.

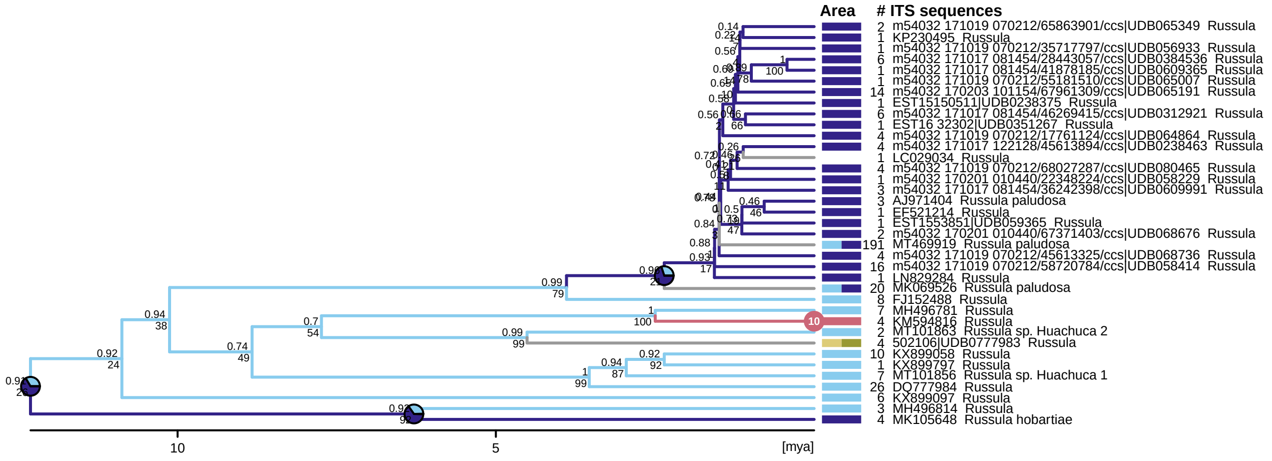

Figure S3 e

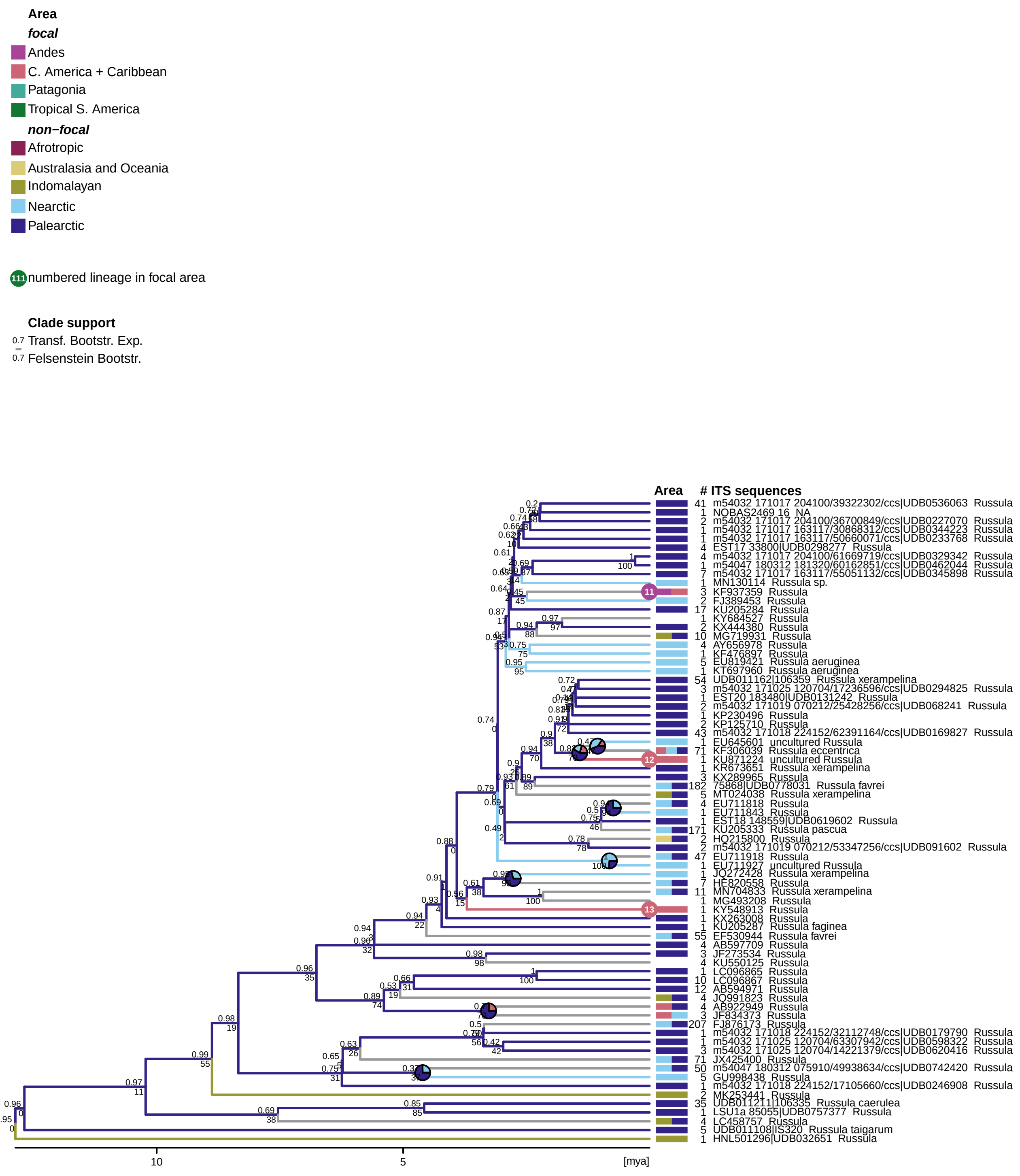
Area
focal
Andes
C. America + Caribbean
Patagonia
Tropical S. America
non−focal
Afrotropic
Australasia and Oceania
Indomalayan
Nearctic
Palearctic
111 numbered lineage in focal area
Clade support
0.7 Transf. Bootstr. Exp.
0.7 Felsenstein Bootstr.
Area
ITS sequences
41 m54032 171017 204100/39322302/ccs|UDB0536063 Russula
1 NOBAS2469 16 NA
2 m54032 171017 204100/36700849/ccs|UDB0227070 Russula
1 m54032 171017 163117/30868312/ccs|UDB0344223 Russula
1 m54032 171017 163117/50660071/ccs|UDB0233768 Russula
4 EST17 33800|UDB0298277 Russula
4 m54032 171017 204100/61669719/ccs|UDB0329342 Russula
1 m54047 180312 181320/60162851/ccs|UDB0462044 Russula
7 m54032 171017 163117/55051132/ccs|UDB0345898 Russula
1 MN130114 Russula sp.
11
3 KF937359 Russula
2 FJ389453 Russula
17 KU205284 Russula
1 KY684527 Russula
2 KX444380 Russula
10 MG719931 Russula
4 AY656978 Russula
1 KF476897 Russula
5 EU819421 Russula aeruginea
1 KT697960 Russula aeruginea
54 UDB011162|106359 Russula xerampelina
3 m54032 171025 120704/17236596/ccs|UDB0294825 Russula
1 EST20 183480|UDB0131242 Russula
2 m54032 171019 070212/25428256/ccs|UDB068241 Russula
1 KP230496 Russula
2 KP125710 Russula
43 m54032 171018 224152/62391164/ccs|UDB0169827 Russula
1 EU645601 uncultured Russula
71 KF306039 Russula eccentrica
12
1 KU871224 uncultured Russula
1 KR673651 Russula xerampelina
3 KX289965 Russula
182 75868|UDB0778031 Russula favrei
5 MT024038 Russula xerampelina
4 EU711818 Russula
1 EU711843 Russula
1 EST18 148559|UDB0619602 Russula
171 KU205333 Russula pascua
2 HQ215800 Russula
2 m54032 171019 070212/53347256/ccs|UDB091602 Russula
47 EU711918 Russula
1 EU711927 uncultured Russula
1 JQ272428 Russula xerampelina
7 HE820558 Russula
11 MN704833 Russula xerampelina
1 MG493208 Russula
13
1 KY548913 Russula
1 KX263008 Russula
1 KU205287 Russula faginea
55 EF530944 Russula favrei
4 AB597709 Russula
3 JF273534 Russula
4 KU550125 Russula
1 LC096865 Russula
10 LC096867 Russula
12 AB594971 Russula
4 JQ991823 Russula
4 AB922949 Russula
3 JF834373 Russula
207 FJ876173 Russula
1 m54032 171018 224152/32112748/ccs|UDB0179790 Russula
1 m54032 171025 120704/63307942/ccs|UDB0598322 Russula
3 m54032 171025 120704/14221379/ccs|UDB0620416 Russula
71 JX425400 Russula
50 m54047 180312 075910/49938634/ccs|UDB0742420 Russula
5 GU998438 Russula
1 m54032 171018 224152/17105660/ccs|UDB0246908 Russula
2 MK253441 Russula
35 UDB011211|106335 Russula caerulea
1 LSU1a 85055|UDB0757377 Russula
4 LC458757 Russula
5 UDB011108|IS320 Russula taigarum
1 HNL501296|UDB032651 Russula
10
5
[mya]

Figure S3 f

Area

focal

Andes

C. America + Caribbean

Patagonia

Tropical S. America

non−focal

Afrotropic

Australasia and Oceania

Indomalayan

Nearctic

Palearctic

111 numbered lineage in focal area

Clade support

0.7 Transf. Bootstr. Exp.

0.7 Felsenstein Bootstr.

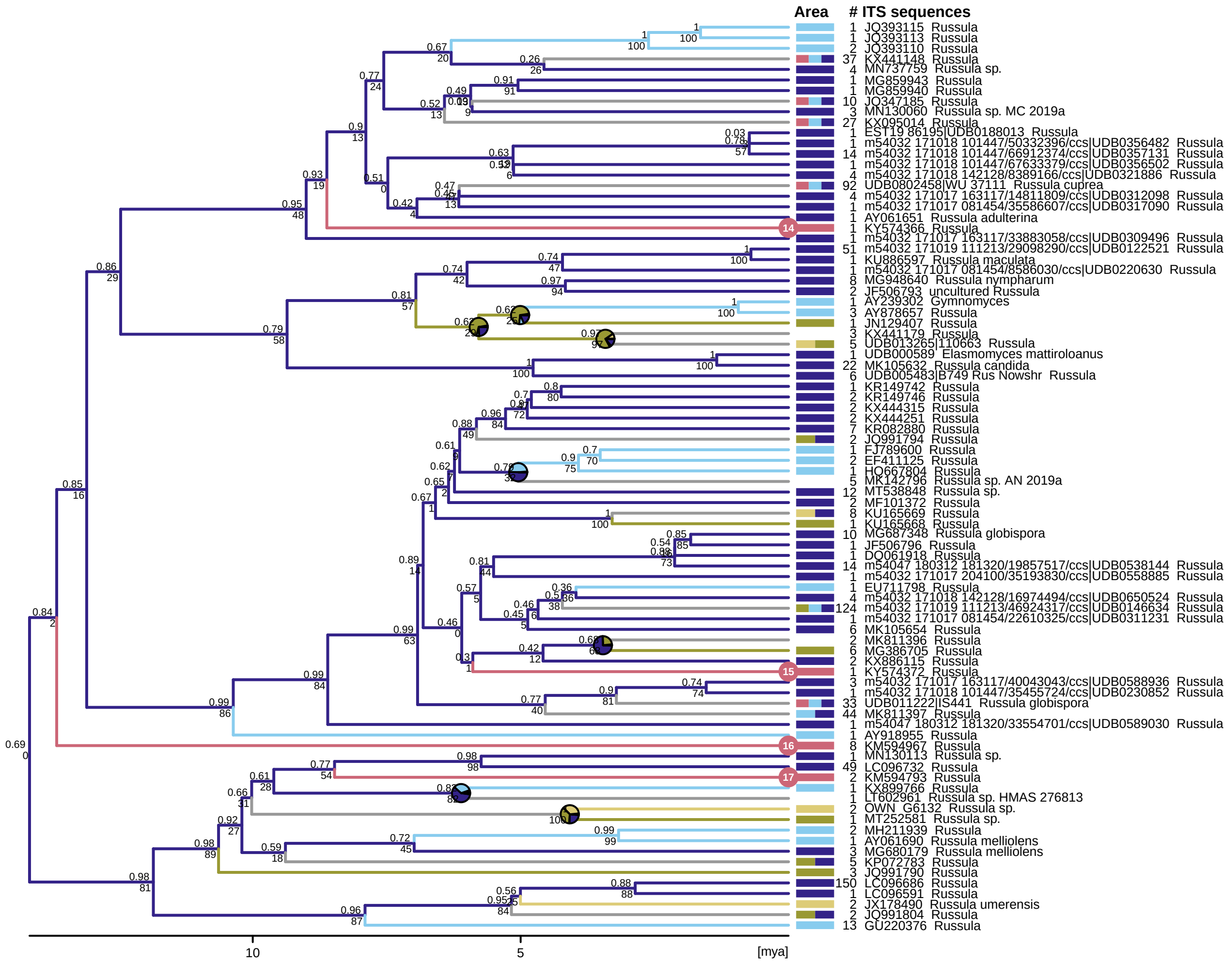

Figure S3 g

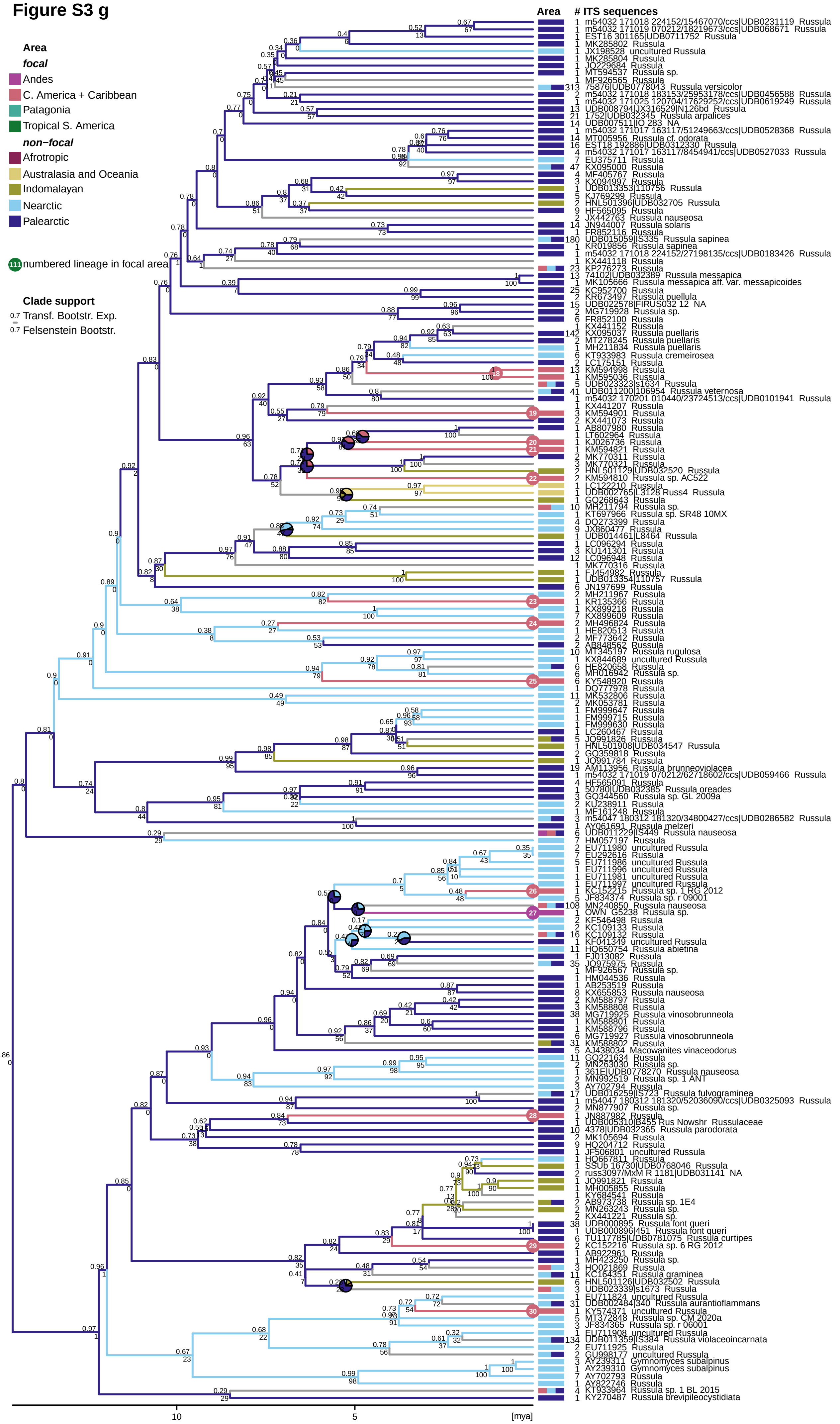

Area
focal
Andes
C. America + Caribbean
Patagonia
Tropical S. America
non−focal
Afrotropic
Australasia and Oceania
Indomalayan
Nearctic
Palearctic
111 numbered lineage in focal area
Clade support
0.7 Transf. Bootstr. Exp.
0.7 Felsenstein Bootstr.
Area
ITS sequences
1 m54032 171018 224152/15467070/ccs|UDB0231119 Russula
1 m54032 171019 070212/18219673/ccs|UDB068671 Russula
1 EST16 301165|UDB0711752 Russula
1 MK285802 Russula
1 JX198528 uncultured Russula
1 MK285804 Russula
1 JQ229684 Russula
1 MT594537 Russula sp.
1 MF926565 Russula
313 75876|UDB0778043 Russula versicolor
2 m54032 171018 183153/25953178/ccs|UDB0456588 Russula
1 m54032 171025 120704/17629252/ccs|UDB0619249 Russula
13 UDB008794|JX316529|N126bd Russula
21 1752|UDB032345 Russula arpalices
14 UDB007511|IO 283 NA
1 m54032 171017 163117/51249663/ccs|UDB0528368 Russula
14 MT005956 Russula cf. odorata
16 EST18 192886|UDB0312330 Russula
4 m54032 171017 163117/8454941/ccs|UDB0527033 Russula
7 EU375711 Russula
47 KX095000 Russula
4 MF405767 Russula
3 KX094997 Russula
1 UDB013353|110756 Russula
5 KJ769299 Russula
2 HNL501396|UDB032705 Russula
9 HF565095 Russula
2 JX442763 Russula nauseosa
14 JN944007 Russula solaris
1 FR852116 Russula
180 UDB015059|IS335 Russula sapinea
1 KR019856 Russula sapinea
1 m54032 171018 224152/27198135/ccs|UDB0183426 Russula
1 KX441118 Russula
23 KP276273 Russula
13 74102|UDB032389 Russula messapica
1 MK105666 Russula messapica aff. var. messapicoides
25 KC952700 Russula
2 KR673497 Russula puellula
15 UDB022578|FIRUS032 12 NA
2 MG719928 Russula sp.
6 FR852100 Russula
1 KX441152 Russula
142 KX095037 Russula puellaris
2 MT278245 Russula puellaris
1 MH211834 Russula puellaris
6 KT933983 Russula cremeirosea
2 LC175151 Russula
13 KM594998 Russula
1 KM595036 Russula
5 UDB023323|s1634 Russula
41 UDB011200|106954 Russula veternosa
1 m54032 170201 010440/23724513/ccs|UDB0101941 Russula
1 KX441207 Russula
3 KM594901 Russula
2 KX441073 Russula
1 AB807980 Russula
1 LT602964 Russula
1 KJ026736 Russula
1 KM594821 Russula
2 MK770311 Russula
3 MK770321 Russula
2 HNL501129|UDB032520 Russula
2 KM594810 Russula sp. AC522
1 LC122210 Russula
1 UDB002765|L3128 Russ4 Russula
1 GQ268643 Russula
10 MH211794 Russula sp.
1 KT697966 Russula sp. SR48 10MX
4 DQ273399 Russula
9 JX860477 Russula
1 UDB014461|L8464 Russula
1 LC096294 Russula
3 KU141301 Russula
12 LC096948 Russula
1 MK770316 Russula
1 FJ454982 Russula
1 UDB013354|110757 Russula
6 JN197699 Russula
2 MH211967 Russula
1 KR135366 Russula
1 KX899218 Russula
7 KX899609 Russula
2 MH496824 Russula
1 HE820513 Russula
2 MF773642 Russula
2 AB848562 Russula
10 MT345197 Russula rugulosa
1 KX844689 uncultured Russula
6 HE820658 Russula
6 MH016942 Russula sp.
6 KY548920 Russula
1 DQ777978 Russula
11 MK532806 Russula
2 MK053781 Russula
1 FM999647 Russula
1 FM999715 Russula
1 FM999630 Russula
1 LC260467 Russula
5 JQ991826 Russula
1 HNL501908|UDB034547 Russula
2 GQ359818 Russula
1 JQ991784 Russula
19 AM113956 Russula brunneoviolacea
1 m54032 171019 070212/62718602/ccs|UDB059466 Russula
4 HF565091 Russula
1 50780|UDB032385 Russula oreades
3 GQ344560 Russula sp. GL 2009a
2 KU238911 Russula
1 MF161248 Russula
3 m54047 180312 181320/34800427/ccs|UDB0286582 Russula
1 AY061691 Russula melzeri
6 UDB011229|IS449 Russula nauseosa
7 HM057197 Russula
2 EU711980 uncultured Russula
7 EU292616 Russula
5 EU711986 uncultured Russula
1 EU711996 uncultured Russula
1 EU711981 uncultured Russula
1 EU711997 uncultured Russula
1 KC152215 Russula sp. 1 RG 2012
5 JF834374 Russula sp. r 09001
108 MN240850 Russula nauseosa
1 OWN G5238 Russula sp.
2 KF546498 Russula
2 KC109133 Russula
16 KC109132 Russula
1 KF041349 uncultured Russula
11 HQ650754 Russula abietina
1 FJ013082 Russula
35 JQ975975 Russula
1 MF926567 Russula sp.
1 HM044536 Russula
1 AB253519 Russula
8 KX655853 Russula nauseosa
2 KM588797 Russula
3 KM588808 Russula
38 MG719925 Russula vinosobrunneola
1 KM588801 Russula
1 KM588796 Russula
6 MG719927 Russula vinosobrunneola
31 KM588802 Russula
5 AJ438034 Macowanites vinaceodorus
11 GQ221634 Russula
2 MN263030 Russula sp.
1 361E|UDB0778270 Russula nauseosa
2 MN992519 Russula sp. 1 ANT
3 AY702794 Russula
17 UDB016259|IS723 Russula fulvograminea
1 m54047 180312 181320/52036090/ccs|UDB0325093 Russula
2 MN877907 Russula sp.
1 JN887982 Russula
1 UDB005310|B455 Rus Nowshr Russulaceae
10 4378|UDB032365 Russula parodorata
2 MK105694 Russula
9 HQ204712 Russula
1 JF506801 uncultured Russula
1 HQ667811 Russula
1 SSUb 16730|UDB0768046 Russula
2 russ3097/MxM R 1181|UDB031141 NA
1 JQ991821 Russula
1 MH005855 Russula
1 KY684541 Russula
2 AB973738 Russula sp. 1E4
2 MN263243 Russula sp.
2 KX441221 Russula sp.
38 UDB000895 Russula font queri
1 UDB000896|451 Russula font queri
6 TU117785|UDB0781075 Russula curtipes
2 KC152216 Russula sp. 6 RG 2012
1 AB922961 Russula
1 MH423250 Russula sp.
3 HQ021869 Russula
11 KC164351 Russula graminea
6 HNL501126|UDB032502 Russula
3 UDB023339|s1673 Russula
1 EU711824 uncultured Russula
31 UDB002484|340 Russula aurantioflammans
1 KY574371 uncultured Russula
5 MT372848 Russula sp. CM 2020a
3 JF834365 Russula sp. r 06001
1 EU711908 uncultured Russula
134 UDB011359|IS384 Russula violaceoincarnata
2 EU711925 Russula
2 GU998177 uncultured Russula
3 AY239311 Gymnomyces subalpinus
1 AY239310 Gymnomyces subalpinus
7 AY702793 Russula
1 AY822746 Russula
4 KT933964 Russula sp. 1 BL 2015
1 KY270487 Russula brevipileocystidiata
18 19 20 21 22 23 24 25 26 27 28 29 30
10
5
[mya]

Figure S3 h

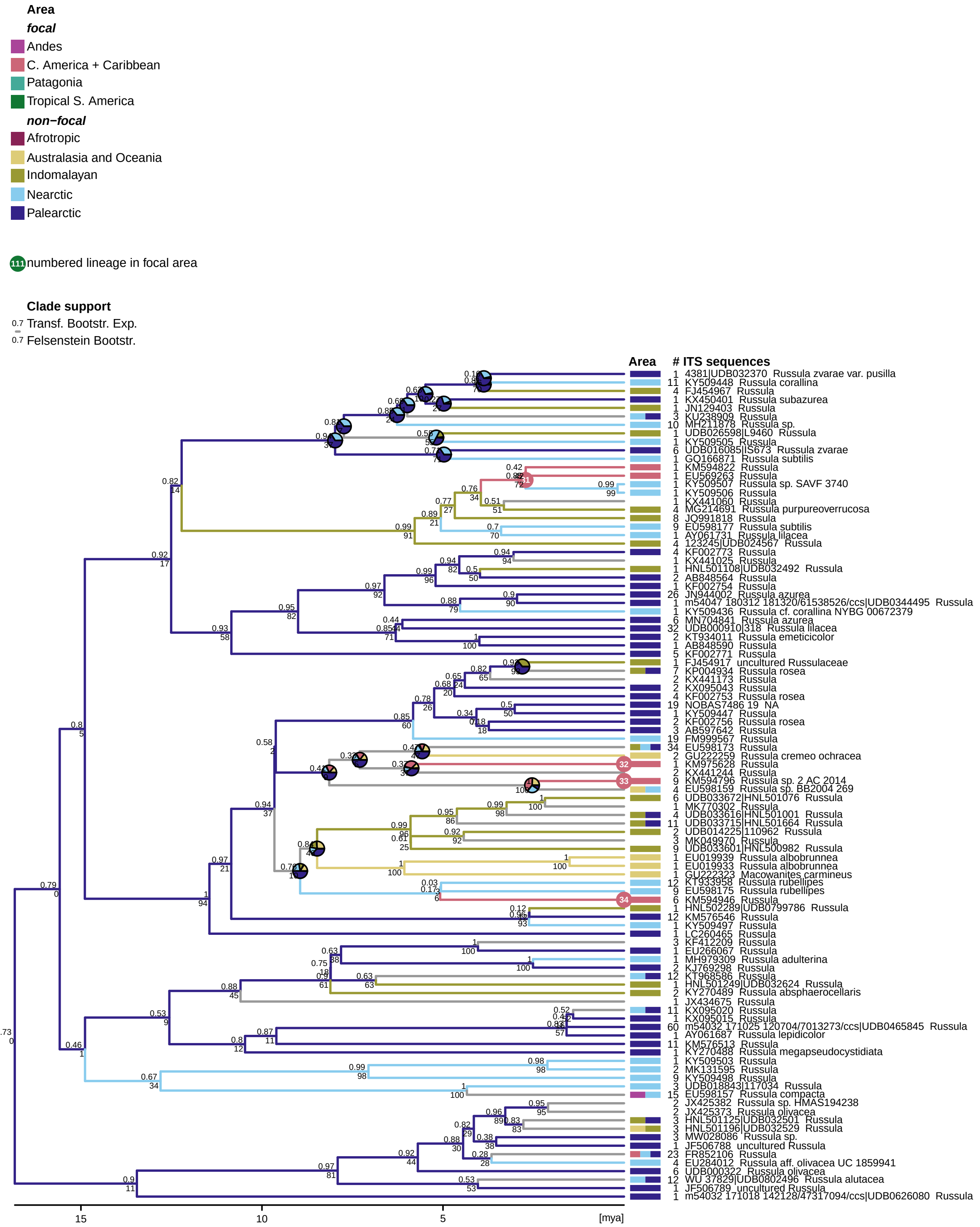
Area
focal
Andes
C. America + Caribbean
Patagonia
Tropical S. America
non−focal
Afrotropic
Australasia and Oceania
Indomalayan
Nearctic
Palearctic
111 numbered lineage in focal area
Clade support
0.7 Transf. Bootstr. Exp.
0.7 Felsenstein Bootstr.
Area
ITS sequences
1 4381|UDB032370 Russula zvarae var. pusilla
11 KY509448 Russula corallina
4 FJ454967 Russula
1 KX450401 Russula subazurea
1 JN129403 Russula
3 KU238909 Russula
10 MH211878 Russula sp.
1 UDB026598|L9460 Russula
1 KY509505 Russula
6 UDB016085|IS673 Russula zvarae
1 GQ166871 Russula subtilis
1 KM594822 Russula
1 EU569263 Russula
1 KY509507 Russula sp. SAVF 3740
1 KY509506 Russula
1 KX441060 Russula
4 MG214691 Russula purpureoverrucosa
8 JQ991818 Russula
9 EU598177 Russula subtilis
1 AY061731 Russula lilacea
4 123245|UDB024567 Russula
4 KF002773 Russula
1 KX441025 Russula
1 HNL501108|UDB032492 Russula
2 AB848564 Russula
1 KF002754 Russula
26 JN944002 Russula azurea
1 m54047 180312 181320/61538526/ccs|UDB0344495 Russula
1 KY509436 Russula cf. corallina NYBG 00672379
6 MN704841 Russula azurea
32 UDB000910|318 Russula lilacea
2 KT934011 Russula emeticicolor
1 AB848590 Russula
5 KF002771 Russula
1 FJ454917 uncultured Russulaceae
7 KP004934 Russula rosea
2 KX441173 Russula
2 KX095043 Russula
4 KF002753 Russula rosea
19 NOBAS7486 19 NA
1 KY509447 Russula
2 KF002756 Russula rosea
3 AB597642 Russula
19 FM999567 Russula
34 EU598173 Russula
2 GU222259 Russula cremeo ochracea
32
1 KM975628 Russula
2 KX441244 Russula
33
9 KM594796 Russula sp. 2 AC 2014
4 EU598159 Russula sp. BB2004 269
6 UDB033672|HNL501076 Russula
1 MK770302 Russula
4 UDB033616|HNL501001 Russula
11 UDB033715|HNL501664 Russula
2 UDB014225|110962 Russula
3 MK049970 Russula
9 UDB033601|HNL500982 Russula
1 EU019939 Russula albobrunnea
1 EU019933 Russula albobrunnea
1 GU222323 Macowanites carmineus
12 KT933958 Russula rubellipes
9 EU598175 Russula rubellipes
34
6 KM594946 Russula
1 HNL502289|UDB0799786 Russula
12 KM576546 Russula
1 KY509497 Russula
1 LC260465 Russula
3 KF412209 Russula
1 EU266067 Russula
1 MH979309 Russula adulterina
2 KJ769298 Russula
12 KT968586 Russula
1 HNL501249|UDB032624 Russula
2 KY270489 Russula absphaerocellaris
1 JX434675 Russula
11 KX095020 Russula
1 KX095015 Russula
60 m54032 171025 120704/7013273/ccs|UDB0465845 Russula
1 AY061687 Russula lepidicolor
11 KM576513 Russula
1 KY270488 Russula megapseudocystidiata
1 KY509503 Russula
2 MK131595 Russula
9 KY509498 Russula
3 UDB018843|117034 Russula
15 EU598157 Russula compacta
2 JX425382 Russula sp. HMAS194238
2 JX425373 Russula olivacea
3 HNL501125|UDB032501 Russula
3 HNL501196|UDB032529 Russula
3 MW028086 Russula sp.
1 JF506788 uncultured Russula
23 FR852106 Russula
4 EU284012 Russula aff. olivacea UC 1859941
6 UDB000322 Russula olivacea
12 WU 37829|UDB0802496 Russula alutacea
1 JF506789 uncultured Russula
1 m54032 171018 142128/47317094/ccs|UDB0626080 Russula
15
10
5
[mya]

Figure S3 i

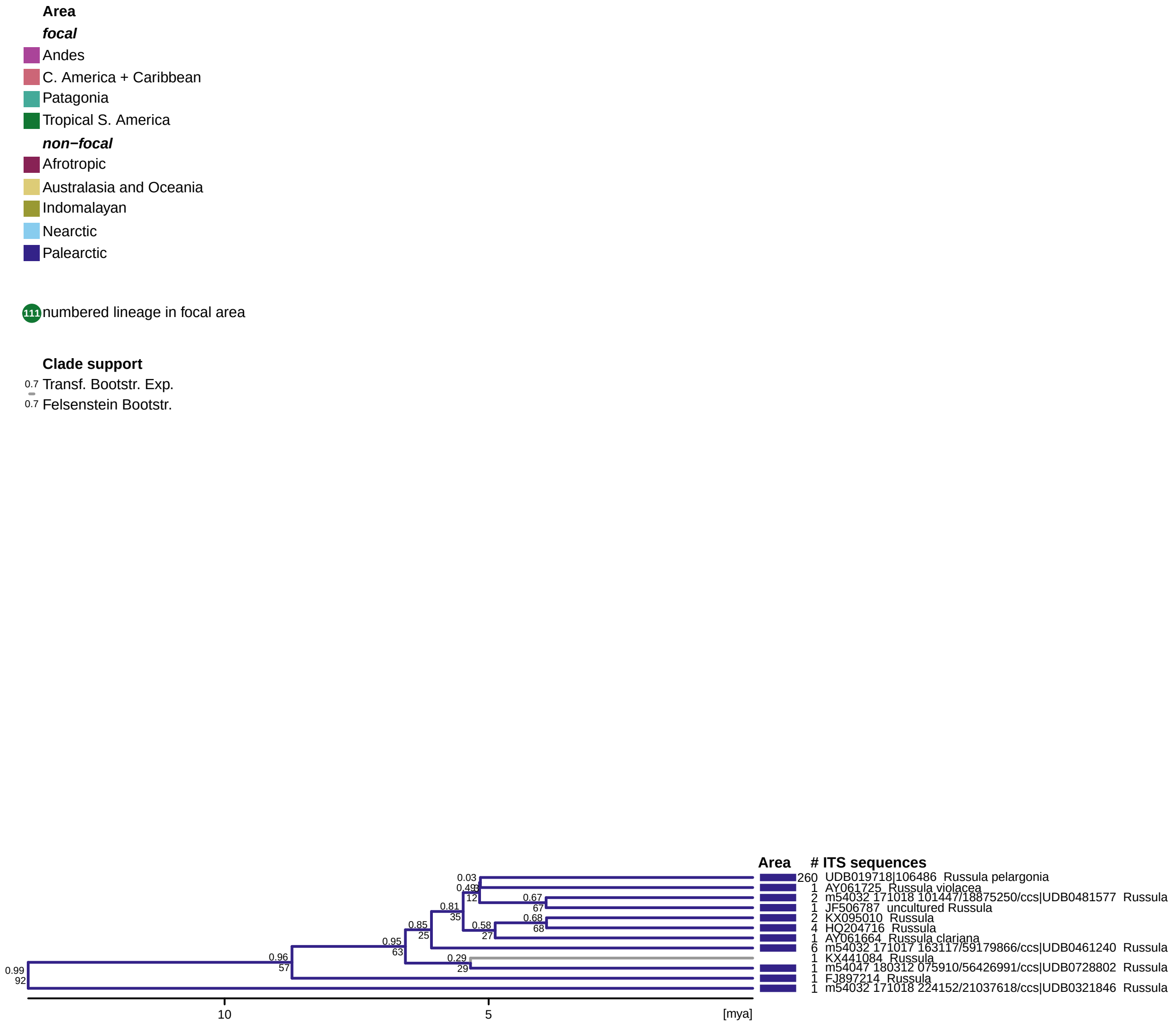
Area
focal
Andes
C. America + Caribbean
Patagonia
Tropical S. America
non−focal
Afrotropic
Australasia and Oceania
Indomalayan
Nearctic
Palearctic
111 numbered lineage in focal area
Clade support
0.7 Transf. Bootstr. Exp.
0.7 Felsenstein Bootstr.
0.03
0.49
12
0.67
67
0.81
35
0.68
68
0.85
25
0.58
27
0.95
63
0.29
29
0.96
57
0.99
92
10
5
[mya]
Area
ITS sequences
260 UDB019718|106486 Russula pelargonia
1 AY061725 Russula violacea
2 m54032 171018 101447/18875250/ccs|UDB0481577 Russula
1 JF506787 uncultured Russula
2 KX095010 Russula
4 HQ204716 Russula
1 AY061664 Russula clariana
6 m54032 171017 163117/59179866/ccs|UDB0461240 Russula
1 KX441084 Russula
1 m54047 180312 075910/56426991/ccs|UDB0728802 Russula
1 FJ897214 Russula
1 m54032 171018 224152/21037618/ccs|UDB0321846 Russula

Figure S3 j

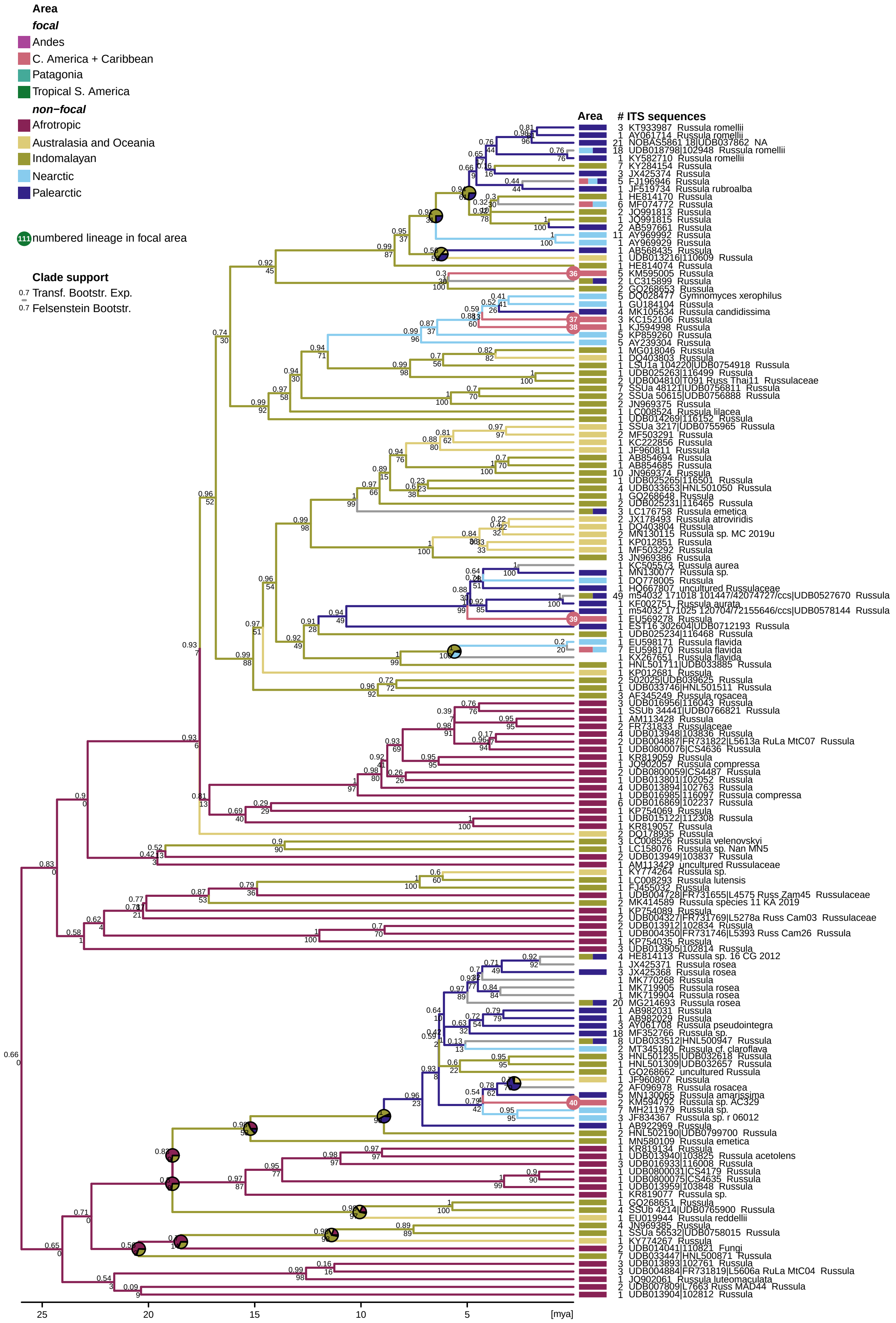

Area
focal
Andes
C. America + Caribbean
Patagonia
Tropical S. America
non−focal
Afrotropic
Australasia and Oceania
Indomalayan
Nearctic
Palearctic
111 numbered lineage in focal area
Clade support
0.7 Transf. Bootstr. Exp.
0.7 Felsenstein Bootstr.
Area
ITS sequences
3 KT933987 Russula romellii
1 AY061714 Russula romellii
21 NOBAS5861 18|UDB037862 NA
18 UDB018798|102948 Russula romellii
1 KY582710 Russula romellii
7 KY284154 Russula
3 JX425374 Russula
5 FJ196946 Russula
1 JF519734 Russula rubroalba
1 HE814170 Russula
6 MF074772 Russula
2 JQ991813 Russula
1 JQ991815 Russula
2 AB597661 Russula
11 AY969992 Russula
1 AY969929 Russula
1 AB568435 Russula
1 UDB013216|110609 Russula
1 HE814074 Russula
5 KM595005 Russula
2 LC315899 Russula
2 GQ268653 Russula
5 DQ028477 Gymnomyces xerophilus
1 GU184104 Russula
4 MK105634 Russula candidissima
3 KC152106 Russula
1 KJ594998 Russula
5 KP859260 Russula
5 AY239304 Russula
1 MG018046 Russula
1 DQ403803 Russula
1 LSU1a 104220|UDB0754918 Russula
1 UDB025263|116499 Russula
2 UDB004810|T091 Russ Thai11 Russulaceae
7 SSUa 48121|UDB0756811 Russula
2 SSUa 50615|UDB0756888 Russula
2 JN969375 Russula
1 LC008524 Russula lilacea
1 UDB014269|116152 Russula
1 SSUa 3217|UDB0755965 Russula
2 MF503291 Russula
1 KC222856 Russula
1 JF960811 Russula
1 AB854694 Russula
1 AB854685 Russula
10 JN969374 Russula
1 UDB025265|116501 Russula
4 UDB033653|HNL501050 Russula
1 GQ268648 Russula
2 UDB025231|116465 Russula
3 LC176758 Russula emetica
2 JX178493 Russula atroviridis
1 DQ403804 Russula
2 MN130115 Russula sp. MC 2019u
1 KP012851 Russula
1 MF503292 Russula
3 JN969386 Russula
1 KC505573 Russula aurea
1 MN130077 Russula sp.
1 DQ778005 Russula
1 HQ667807 uncultured Russulaceae
49 m54032 171018 101447/42074727/ccs|UDB0527670 Russula
1 KF002751 Russula aurata
1 m54032 171025 120704/72155646/ccs|UDB0578144 Russula
1 EU569278 Russula
1 EST16 302604|UDB0712193 Russula
1 UDB025234|116468 Russula
1 EU598171 Russula flavida
7 EU598170 Russula flavida
1 KX267651 Russula flavida
1 HNL501711|UDB033885 Russula
1 KP012681 Russula
2 502025|UDB039625 Russula
1 UDB033746|HNL501511 Russula
3 AF345249 Russula rosacea
3 UDB016956|116043 Russula
1 SSUb 34441|UDB0766821 Russula
1 AM113428 Russula
2 FR731833 Russulaceae
4 UDB013948|103836 Russula
2 UDB004887|FR731822|L5613a RuLa MtC07 Russula
1 UDB0800076|CS4636 Russula
1 KR819059 Russula
1 JQ902057 Russula compressa
2 UDB0800059|CS4487 Russula
1 UDB013801|102052 Russula
4 UDB013894|102763 Russula
1 UDB016985|116097 Russula compressa
6 UDB016869|102237 Russula
1 KP754069 Russula
1 UDB015122|112308 Russula
1 KR819057 Russula
2 DQ178935 Russula
3 LC008526 Russula velenovskyi
1 LC158076 Russula sp. Nan MN5
2 UDB013949|103837 Russula
1 AM113429 uncultured Russulaceae
1 KY774264 Russula sp.
1 LC008293 Russula lutensis
1 FJ455032 Russula
1 UDB004728|FR731655|L4575 Russ Zam45 Russulaceae
2 MK414589 Russula species 11 KA 2019
1 KP754089 Russula
2 UDB004327|FR731769|L5278a Russ Cam03 Russulaceae
2 UDB013912|102834 Russula
1 UDB004350|FR731746|L5393 Russ Cam26 Russula
1 KP754035 Russula
3 UDB013905|102814 Russula
4 HE814113 Russula sp. 16 CG 2012
1 JX425371 Russula rosea
3 JX425368 Russula rosea
1 MK770268 Russula
1 MK719905 Russula rosea
1 MK719904 Russula rosea
20 MG214693 Russula rosea
1 AB982031 Russula
1 AB982029 Russula
3 AY061708 Russula pseudointegra
18 MF352766 Russula sp.
8 UDB033512|HNL500947 Russula
2 MT345180 Russula cf. claroflava
3 HNL501235|UDB032618 Russula
1 HNL501309|UDB032657 Russula
1 GQ268662 uncultured Russula
1 JF960807 Russula
2 AF096978 Russula rosacea
5 MN130065 Russula amarissima
2 KM594792 Russula sp. AC329
7 MH211979 Russula sp.
3 JF834367 Russula sp. r 06012
1 AB922969 Russula
2 HNL502190|UDB0799700 Russula
1 MN580109 Russula emetica
1 KR819134 Russula
1 UDB013940|103825 Russula acetolens
3 UDB016933|116008 Russula
1 UDB0800031|CS4179 Russula
1 UDB0800075|CS4635 Russula
1 UDB013959|103848 Russula
1 KR819077 Russula sp.
1 GQ268651 Russula
4 SSUb 4214|UDB0765900 Russula
1 EU019944 Russula reddellii
4 JN969385 Russula
1 SSUa 56532|UDB0758015 Russula
1 KY774267 Russula
2 UDB014041|110821 Fungi
7 UDB033447|HNL500871 Russula
3 UDB013893|102761 Russula
3 UDB004884|FR731819|L5606a RuLa MtC04 Russula
1 JQ902061 Russula luteomaculata
2 UDB007809|L7663 Russ MAD44 Russula
1 UDB013904|102812 Russula
36
37
38
39
40
25
20
15
10
5
[mya]

Figure S3 k

Area

focal

- Andes
- C. America + Caribbean
- Patagonia
- Tropical S. America

non−focal

- Afrotropic
- Australasia and Oceania
- Indomalayan
- Nearctic
- Palearctic

111 numbered lineage in focal area

Clade support

0.7 Transf. Bootstr. Exp.

0.7 Felsenstein Bootstr.

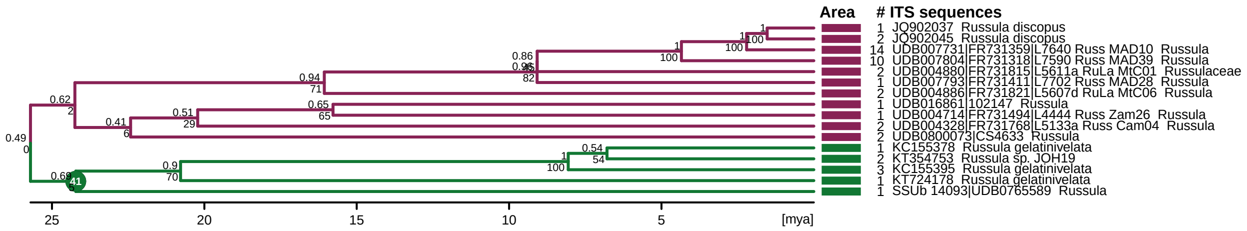

Figure S3 l

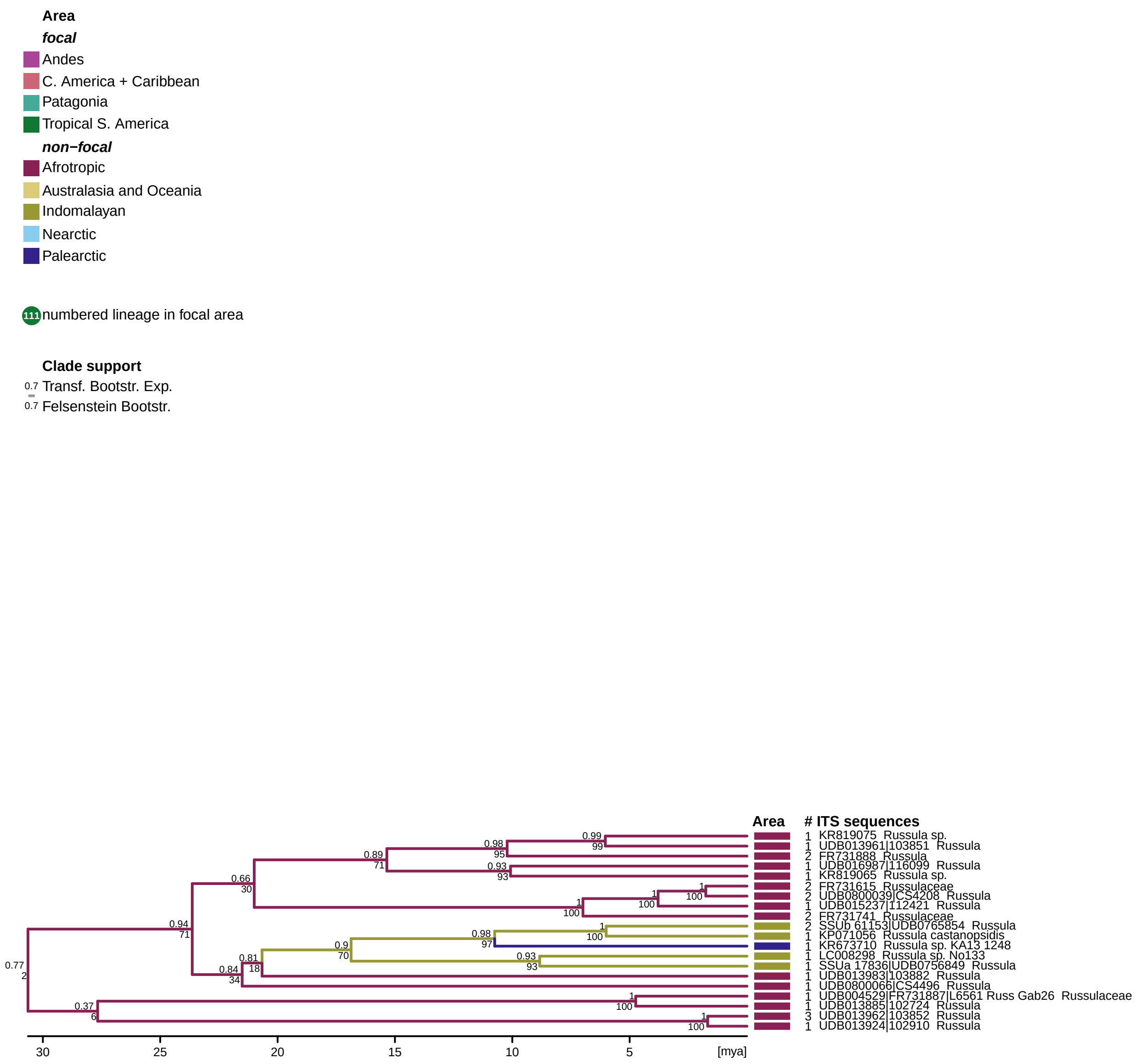

Area
focal
Andes
C. America + Caribbean
Patagonia
Tropical S. America
non−focal
Afrotropic
Australasia and Oceania
Indomalayan
Nearctic
Palearctic
111 numbered lineage in focal area
Clade support
0.7 Transf. Bootstr. Exp.
0.7 Felsenstein Bootstr.
Area
ITS sequences
1 KR819075 Russula sp.
1 UDB013961|103851 Russula
2 FR731888 Russula
1 UDB016987|116099 Russula
1 KR819065 Russula sp.
2 FR731615 Russulaceae
2 UDB0800039|CS4208 Russula
1 UDB015237|112421 Russula
2 FR731741 Russulaceae
2 SSUb 61153|UDB0765854 Russula
1 KP071056 Russula castanopsidis
1 KR673710 Russula sp. KA13 1248
1 LC008298 Russula sp. No133
1 SSUa 17836|UDB0756849 Russula
1 UDB013983|103882 Russula
1 UDB0800066|CS4496 Russula
1 UDB004529|FR731887|L6561 Russ Gab26 Russulaceae
1 UDB013885|102724 Russula
3 UDB013962|103852 Russula
1 UDB013924|102910 Russula
0.99
99
0.98
95
0.89
71
0.93
93
0.66
30
1
100
1
100
1
100
0.94
71
1
100
0.98
97
0.9
70
0.93
93
0.81
18
0.84
34
0.77
2
1
100
0.37
6
1
100
30
25
20
15
10
5
[mya]

Figure S3 m

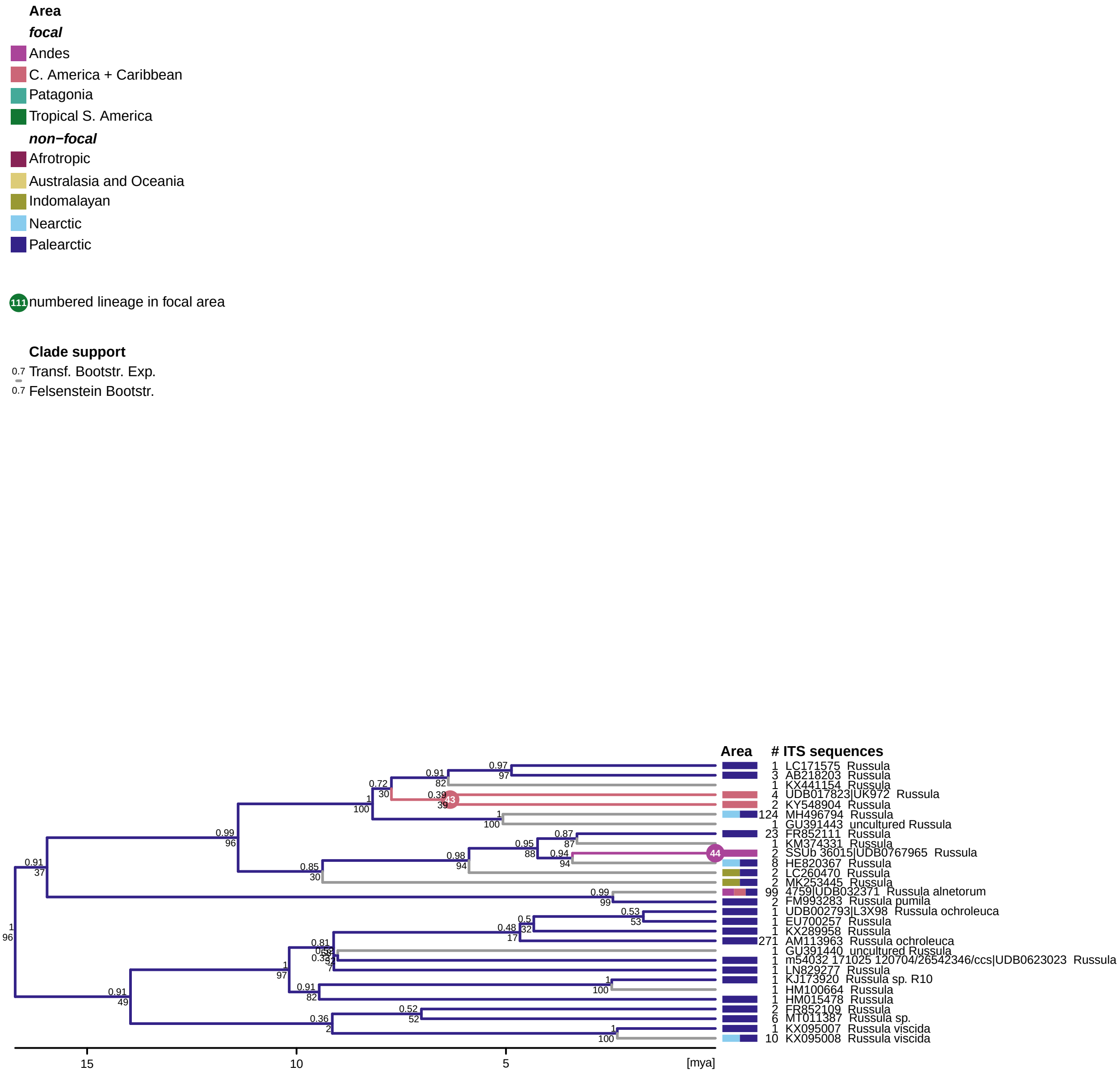

Area
focal
Andes
C. America + Caribbean
Patagonia
Tropical S. America
non−focal
Afrotropic
Australasia and Oceania
Indomalayan
Nearctic
Palearctic
111 numbered lineage in focal area
Clade support
0.7 Transf. Bootstr. Exp.
0.7 Felsenstein Bootstr.
Area
ITS sequences
1 LC171575 Russula
3 AB218203 Russula
1 KX441154 Russula
4 UDB017823|UK972 Russula
2 KY548904 Russula
124 MH496794 Russula
1 GU391443 uncultured Russula
23 FR852111 Russula
1 KM374331 Russula
2 SSUb 36015|UDB0767965 Russula
8 HE820367 Russula
2 LC260470 Russula
2 MK253445 Russula
99 4759|UDB032371 Russula alnetorum
2 FM993283 Russula pumila
1 UDB002793|L3X98 Russula ochroleuca
1 EU700257 Russula
1 KX289958 Russula
271 AM113963 Russula ochroleuca
1 GU391440 uncultured Russula
1 m54032 171025 120704/26542346/ccs|UDB0623023 Russula
1 LN829277 Russula
1 KJ173920 Russula sp. R10
1 HM100664 Russula
1 HM015478 Russula
2 FR852109 Russula
6 MT011387 Russula sp.
1 KX095007 Russula viscida
10 KX095008 Russula viscida
44
15
10
5
[mya]

Figure S3 n

Area

focal

- Andes
- C. America + Caribbean
- Patagonia
- Tropical S. America

non−focal

- Afrotropic
- Australasia and Oceania
- Indomalayan
- Nearctic
- Palearctic

111 numbered lineage in focal area

Clade support

0.7 Transf. Bootstr. Exp.
0.7 Felsenstein Bootstr.

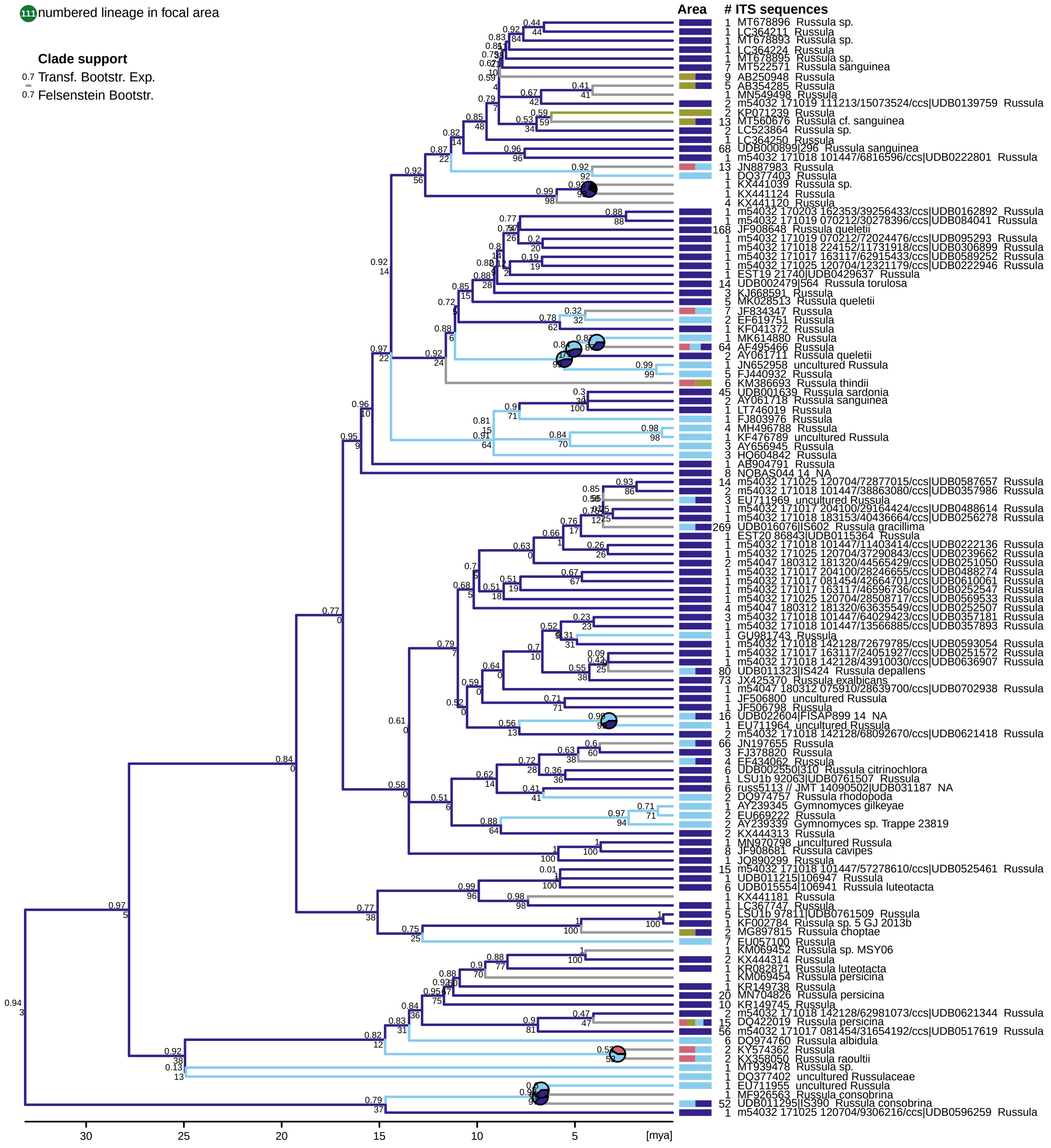

Figure S3 o

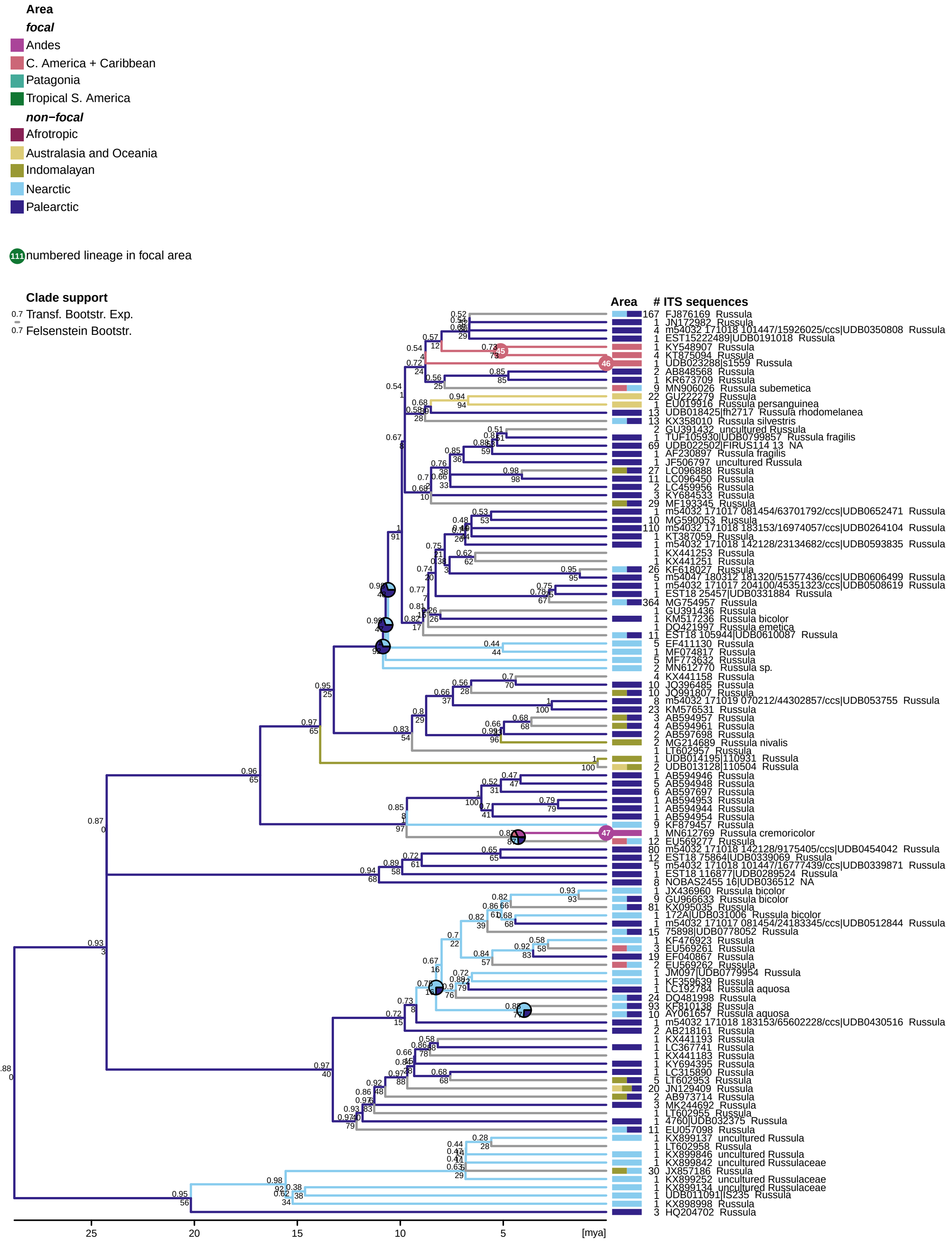

Area
focal
Andes
C. America + Caribbean
Patagonia
Tropical S. America
non−focal
Afrotropic
Australasia and Oceania
Indomalayan
Nearctic
Palearctic
111 numbered lineage in focal area
Clade support
0.7 Transf. Bootstr. Exp.
0.7 Felsenstein Bootstr.
Area
ITS sequences
167 FJ876169 Russula
1 JN172982 Russula
4 m54032 171018 101447/15926025/ccs|UDB0350808 Russula
1 EST15222489|UDB0191018 Russula
1 KY548907 Russula
4 KT875094 Russula
1 UDB023288|s1559 Russula
2 AB848568 Russula
1 KR673709 Russula
9 MN906026 Russula subemetica
22 GU222279 Russula
1 EU019916 Russula persanguinea
13 UDB018425|fh2717 Russula rhodomelanea
13 KX358010 Russula silvestris
2 GU391432 uncultured Russula
1 TUF105930|UDB0799857 Russula fragilis
69 UDB022502|FIRUS114 13 NA
1 AF230897 Russula fragilis
1 JF506797 uncultured Russula
27 LC096888 Russula
11 LC096450 Russula
2 LC459956 Russula
3 KY684533 Russula
29 MF193345 Russula
1 m54032 171017 081454/63701792/ccs|UDB0652471 Russula
10 MG590053 Russula
110 m54032 171018 183153/16974057/ccs|UDB0264104 Russula
1 KT387059 Russula
1 m54032 171018 142128/23134682/ccs|UDB0593835 Russula
1 KX441253 Russula
1 KX441251 Russula
26 KF618027 Russula
5 m54047 180312 181320/51577436/ccs|UDB0606499 Russula
1 m54032 171017 204100/45351323/ccs|UDB0508619 Russula
1 EST18 25457|UDB0331884 Russula
364 MG754957 Russula
1 GU391436 Russula
1 KM517236 Russula bicolor
1 DQ421997 Russula emetica
11 EST18 105944|UDB0610087 Russula
5 EF411130 Russula
1 MF074817 Russula
5 MF773632 Russula
2 MN612770 Russula sp.
4 KX441158 Russula
10 JQ396485 Russula
10 JQ991807 Russula
8 m54032 171019 070212/44302857/ccs|UDB053755 Russula
23 KM576531 Russula
3 AB594957 Russula
4 AB594961 Russula
2 AB597698 Russula
2 MG214689 Russula nivalis
1 LT602957 Russula
1 UDB014195|110931 Russula
2 UDB013128|110504 Russula
1 AB594946 Russula
5 AB594948 Russula
6 AB597697 Russula
1 AB594953 Russula
1 AB594944 Russula
1 AB594954 Russula
9 KF879457 Russula
1 MN612769 Russula cremoricolor
12 EU569277 Russula
80 m54032 171018 142128/9175405/ccs|UDB0454042 Russula
12 EST18 75864|UDB0339069 Russula
5 m54032 171018 101447/16777439/ccs|UDB0339871 Russula
1 EST18 116877|UDB0289524 Russula
8 NOBAS2455 16|UDB036512 NA
1 JX436960 Russula bicolor
9 GU966633 Russula bicolor
81 KX095035 Russula
1 172A|UDB031006 Russula bicolor
1 m54032 171017 081454/24183345/ccs|UDB0512844 Russula
15 75898|UDB0778052 Russula
1 KF476923 Russula
3 EU569261 Russula
19 EF040867 Russula
2 EU569262 Russula
1 JM097|UDB0779954 Russula
1 KF359639 Russula
1 LC192784 Russula aquosa
24 DQ481998 Russula
93 KF810138 Russula
10 AY061657 Russula aquosa
1 m54032 171018 183153/65602228/ccs|UDB0430516 Russula
2 AB218161 Russula
1 KX441193 Russula
1 LC367741 Russula
1 KX441183 Russula
1 KY694395 Russula
1 LC315890 Russula
5 LT602953 Russula
20 JN129409 Russula
2 AB973714 Russula
3 MK244692 Russula
1 LT602955 Russula
1 4760|UDB032375 Russula
11 EU057098 Russula
1 KX899137 uncultured Russula
1 LT602958 Russula
1 KX899846 uncultured Russula
1 KX899842 uncultured Russulaceae
30 JX857186 Russula
1 KX899252 uncultured Russulaceae
1 KX899134 uncultured Russulaceae
1 UDB011091|IS235 Russula
1 KX898998 Russula
3 HQ204702 Russula
25
20
15
10
5
[mya]

Figure S3 p

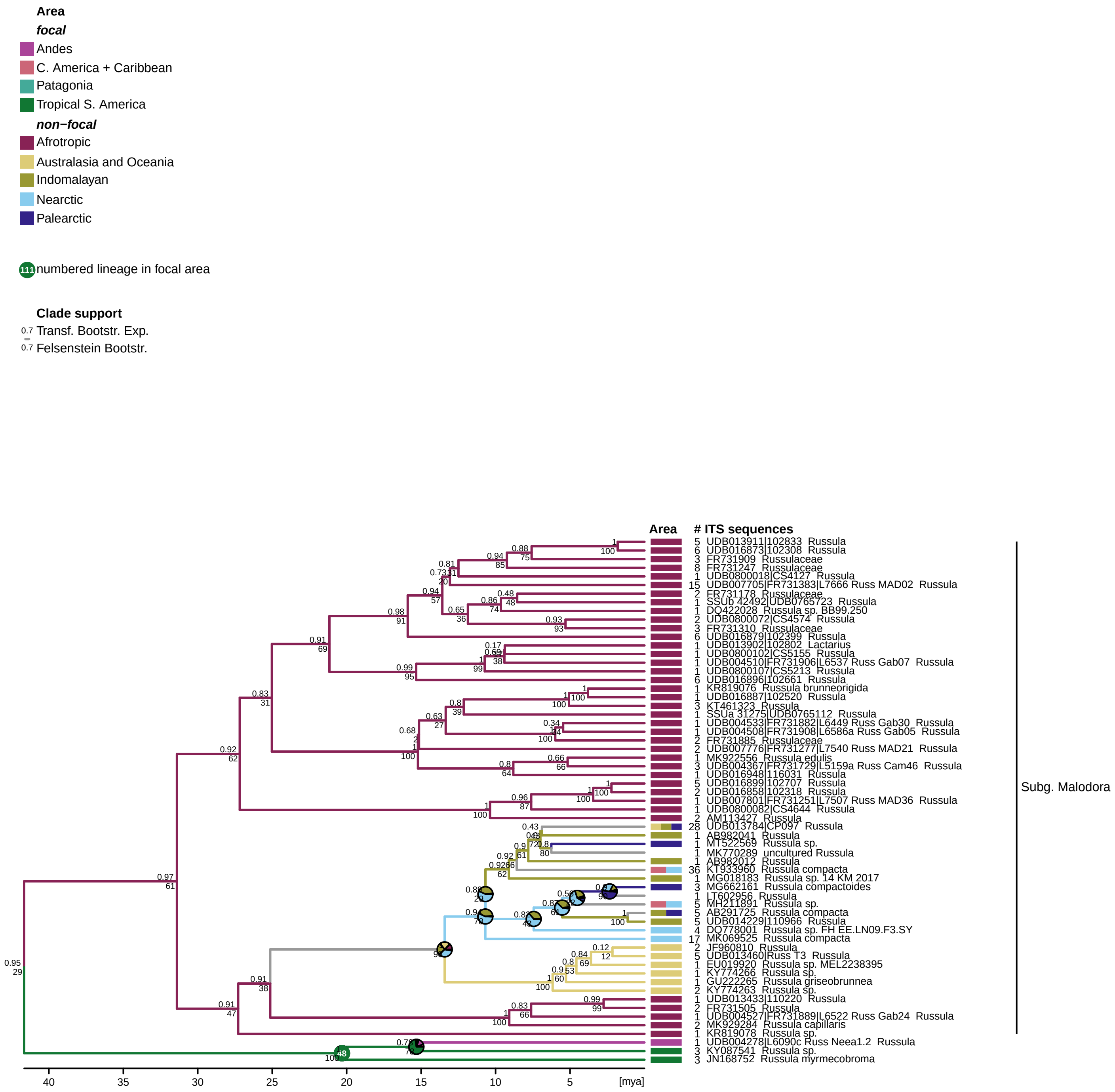
Area
focal
Andes
C. America + Caribbean
Patagonia
Tropical S. America
non−focal
Afrotropic
Australasia and Oceania
Indomalayan
Nearctic
Palearctic
111 numbered lineage in focal area
Clade support
0.7 Transf. Bootstr. Exp.
0.7 Felsenstein Bootstr.
Area
ITS sequences
5 UDB013911|102833 Russula
6 UDB016873|102308 Russula
3 FR731909 Russulaceae
8 FR731247 Russulaceae
1 UDB0800018|CS4127 Russula
15 UDB007705|FR731383|L7666 Russ MAD02 Russula
2 FR731178 Russulaceae
1 SSUb 42492|UDB0765723 Russula
1 DQ422028 Russula sp. BB99.250
2 UDB0800072|CS4574 Russula
3 FR731310 Russulaceae
6 UDB016879|102399 Russula
1 UDB013902|102802 Lactarius
1 UDB0800102|CS5155 Russula
1 UDB004510|FR731906|L6537 Russ Gab07 Russula
1 UDB0800107|CS5213 Russula
6 UDB016896|102661 Russula
1 KR819076 Russula brunneorigida
1 UDB016887|102520 Russula
3 KT461323 Russula
1 SSUa 31275|UDB0765112 Russula
1 UDB004533|FR731882|L6449 Russ Gab30 Russula
1 UDB004508|FR731908|L6586a Russ Gab05 Russula
2 FR731885 Russulaceae
2 UDB007776|FR731277|L7540 Russ MAD21 Russula
1 MK922556 Russula edulis
3 UDB004367|FR731729|L5159a Russ Cam46 Russula
1 UDB016948|116031 Russula
5 UDB016899|102707 Russula
2 UDB016858|102318 Russula
1 UDB007801|FR731251|L7507 Russ MAD36 Russula
1 UDB0800082|CS4644 Russula
2 AM113427 Russula
28 UDB013784|CP097 Russula
1 AB982041 Russula
1 MT522569 Russula sp.
1 MK770289 uncultured Russula
1 AB982012 Russula
36 KT933960 Russula compacta
1 MG018183 Russula sp. 14 KM 2017
3 MG662161 Russula compactoides
1 LT602956 Russula
5 MH211891 Russula sp.
5 AB291725 Russula compacta
5 UDB014229|110966 Russula
4 DQ778001 Russula sp. FH EE.LN09.F3.SY
17 MK069525 Russula compacta
2 JF960810 Russula
5 UDB013460|Russ T3 Russula
1 EU019920 Russula sp. MEL2238395
1 KY774266 Russula sp.
1 GU222265 Russula griseobrunnea
2 KY774263 Russula sp.
1 UDB013433|110220 Russula
2 FR731505 Russula
1 UDB004527|FR731889|L6522 Russ Gab24 Russula
2 MK929284 Russula capillaris
1 KR819078 Russula sp.
1 UDB004278|L6090c Russ Neea1.2 Russula
3 KY087541 Russula sp.
3 JN168752 Russula myrmecobroma
Subg. Malodora
48
40 35 30 25 20 15 10 5 [mya]

Figure S3 q

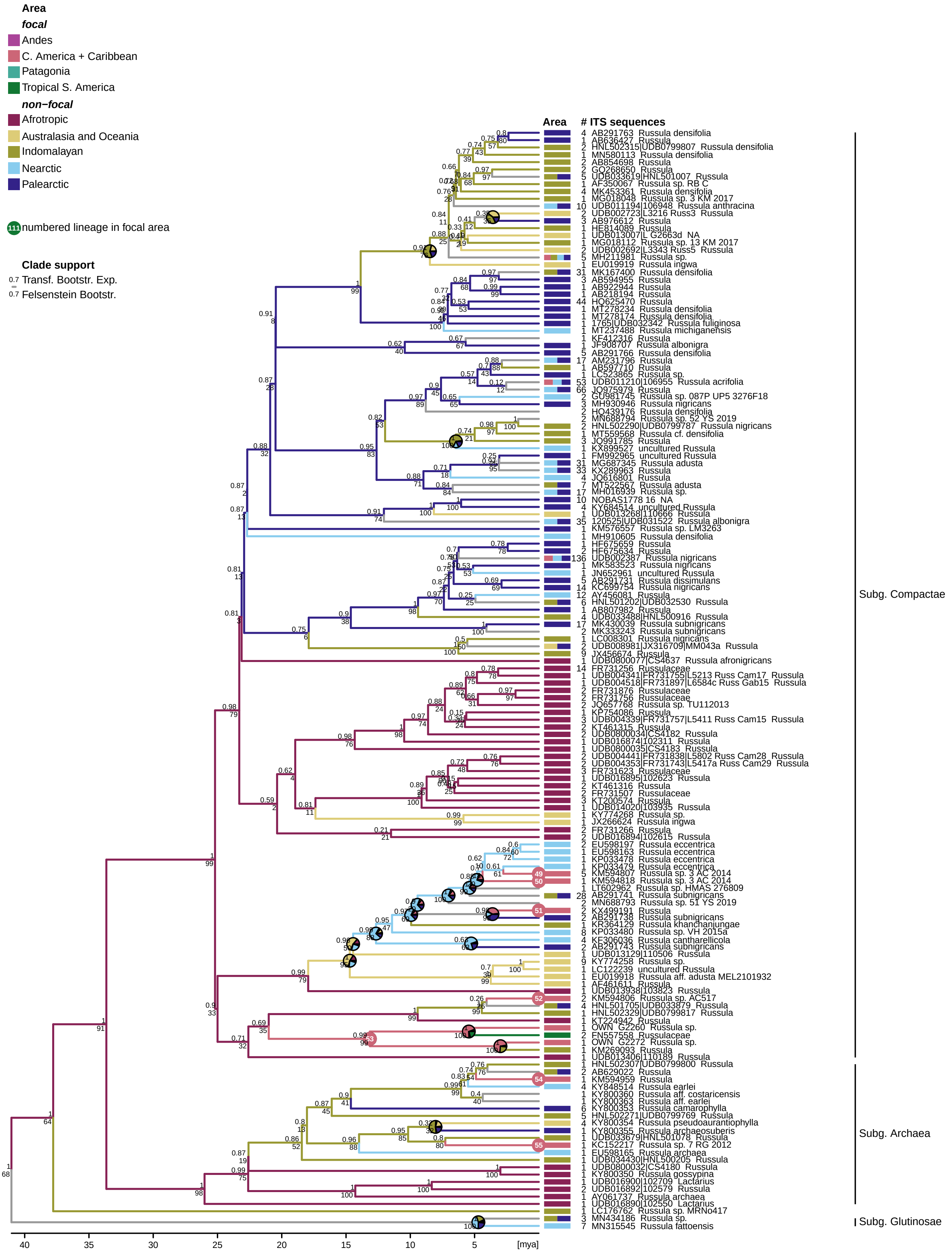

Area
focal
Andes
C. America + Caribbean
Patagonia
Tropical S. America
non−focal
Afrotropic
Australasia and Oceania
Indomalayan
Nearctic
Palearctic
111 numbered lineage in focal area
Clade support
0.7 Transf. Bootstr. Exp.
0.7 Felsenstein Bootstr.
Area
ITS sequences
4 AB291763 Russula densifolia
1 AB636427 Russula
2 HNL502315|UDB0799807 Russula densifolia
1 MN580113 Russula densifolia
2 AB854698 Russula
2 GQ268650 Russula
5 UDB033619|HNL501007 Russula
1 AF350067 Russula sp. RB C
4 MK453361 Russula densifolia
1 MG018048 Russula sp. 3 KM 2017
10 UDB011194|106948 Russula anthracina
2 UDB002723|L3216 Russ3 Russula
3 AB976612 Russula
1 HE814089 Russula
1 UDB013007|L G2663d NA
1 MG018112 Russula sp. 13 KM 2017
2 UDB002692|L3343 Russ5 Russula
5 MH211981 Russula sp.
1 EU019919 Russula ingwa
31 MK167400 Russula densifolia
3 AB594955 Russula
1 AB922944 Russula
1 AB218194 Russula
44 HQ625470 Russula
1 MT278234 Russula densifolia
1 MT278174 Russula densifolia
1 1765|UDB032342 Russula fuliginosa
1 MT237488 Russula michiganensis
1 KF412316 Russula
1 JF908707 Russula albonigra
5 AB291766 Russula densifolia
17 AM231796 Russula
1 AB597710 Russula
1 LC523865 Russula sp.
53 UDB011210|106955 Russula acrifolia
66 JQ975979 Russula
2 GU981745 Russula sp. 087P UP5 3276F18
3 MH930946 Russula nigricans
2 HQ439176 Russula densifolia
2 MN688794 Russula sp. 52 YS 2019
2 HNL502290|UDB0799787 Russula nigricans
1 MT559568 Russula cf. densifolia
3 JQ991785 Russula
1 KX899527 uncultured Russula
1 FM992965 uncultured Russula
31 MG687345 Russula adusta
33 KX289963 Russula
4 JQ616801 Russula
7 MT522567 Russula adusta
17 MH016939 Russula sp.
10 NOBAS1778 16 NA
4 KY684514 uncultured Russula
1 UDB013268|110666 Russula
35 120525|UDB031522 Russula albonigra
1 KM576557 Russula sp. LM3263
1 MH910605 Russula densifolia
1 HF675659 Russula
2 HF675634 Russula
136 UDB002387 Russula nigricans
1 MK583523 Russula nigricans
1 JN652961 uncultured Russula
5 AB291731 Russula dissimulans
14 KC699754 Russula nigricans
12 AY456081 Russula
6 HNL501202|UDB032530 Russula
1 AB807982 Russula
4 UDB033488|HNL500916 Russula
17 MK430039 Russula subnigricans
2 MK333243 Russula subnigricans
1 LC008301 Russula nigricans
2 UDB008981|JX316709|MM043a Russula
9 JX456674 Russula
1 UDB0800077|CS4637 Russula afronigricans
14 FR731256 Russulaceae
1 UDB004341|FR731755|L5213 Russ Cam17 Russula
1 UDB004518|FR731897|L6584c Russ Gab15 Russula
2 FR731876 Russulaceae
2 FR731756 Russulaceae
2 JQ657768 Russula sp. TU112013
1 KP754086 Russula
3 UDB004339|FR731757|L5411 Russ Cam15 Russula
2 KT461315 Russula
2 UDB0800034|CS4182 Russula
1 UDB016874|102311 Russula
1 UDB0800035|CS4183 Russula
2 UDB004441|FR731838|L5802 Russ Cam28 Russula
2 UDB004353|FR731743|L5417a Russ Cam29 Russula
3 FR731623 Russulaceae
1 UDB016895|102623 Russula
2 KT461316 Russula
2 FR731507 Russulaceae
3 KT200574 Russula
1 UDB014020|103935 Russula
1 KY774268 Russula sp.
1 JX266624 Russula ingwa
2 FR731266 Russula
2 UDB016894|102615 Russula
2 EU598197 Russula eccentrica
1 EU598163 Russula eccentrica
1 KP033478 Russula eccentrica
1 KP033479 Russula eccentrica
5 KM594807 Russula sp. 3 AC 2014
1 KM594818 Russula sp. 3 AC 2014
1 LT602962 Russula sp. HMAS 276809
28 AB291741 Russula subnigricans
2 MN688793 Russula sp. 51 YS 2019
2 KX499191 Russula
2 AB291738 Russula subnigricans
1 KR364129 Russula khanchanjungae
8 KP033480 Russula sp. VH 2015a
4 KF306036 Russula cantharellicola
2 AB291743 Russula subnigricans
1 UDB013129|110506 Russula
9 KY774258 Russula sp.
1 LC122239 uncultured Russula
1 EU019918 Russula aff. adusta MEL2101932
1 AF461611 Russula
1 UDB013938|103823 Russula
2 KM594806 Russula sp. AC517
4 HNL501705|UDB033879 Russula
1 HNL502329|UDB0799817 Russula
1 KT224942 Russula
1 OWN G2260 Russula sp.
2 FN557558 Russulaceae
1 OWN G2272 Russula sp.
1 KM269093 Russula
1 UDB013406|110189 Russula
1 HNL502307|UDB0799800 Russula
2 AB629022 Russula
1 KM594959 Russula
4 KY848514 Russula earlei
1 KY800360 Russula aff. costaricensis
1 KY800363 Russula aff. earlei
6 KY800353 Russula camarophylla
6 HNL502271|UDB0799769 Russula
4 KY800354 Russula pseudoaurantiophylla
1 KY800355 Russula archaeosuberis
1 UDB033679|HNL501078 Russula
1 KC152217 Russula sp. 7 RG 2012
1 EU598165 Russula archaea
1 UDB034430|HNL500205 Russula
1 UDB0800032|CS4180 Russula
1 KY800350 Russula gossypina
1 UDB016900|102709 Lactarius
2 UDB016892|102579 Russula
1 AY061737 Russula archaea
1 UDB016890|102550 Lactarius
1 LC176762 Russula sp. MRN0417
3 MN434186 Russula sp.
7 MN315545 Russula fattoensis
Subg. Compactae
Subg. Archaea
Subg. Glutinosae
40 35 30 25 20 15 10 5 [mya]

Figure S3 r

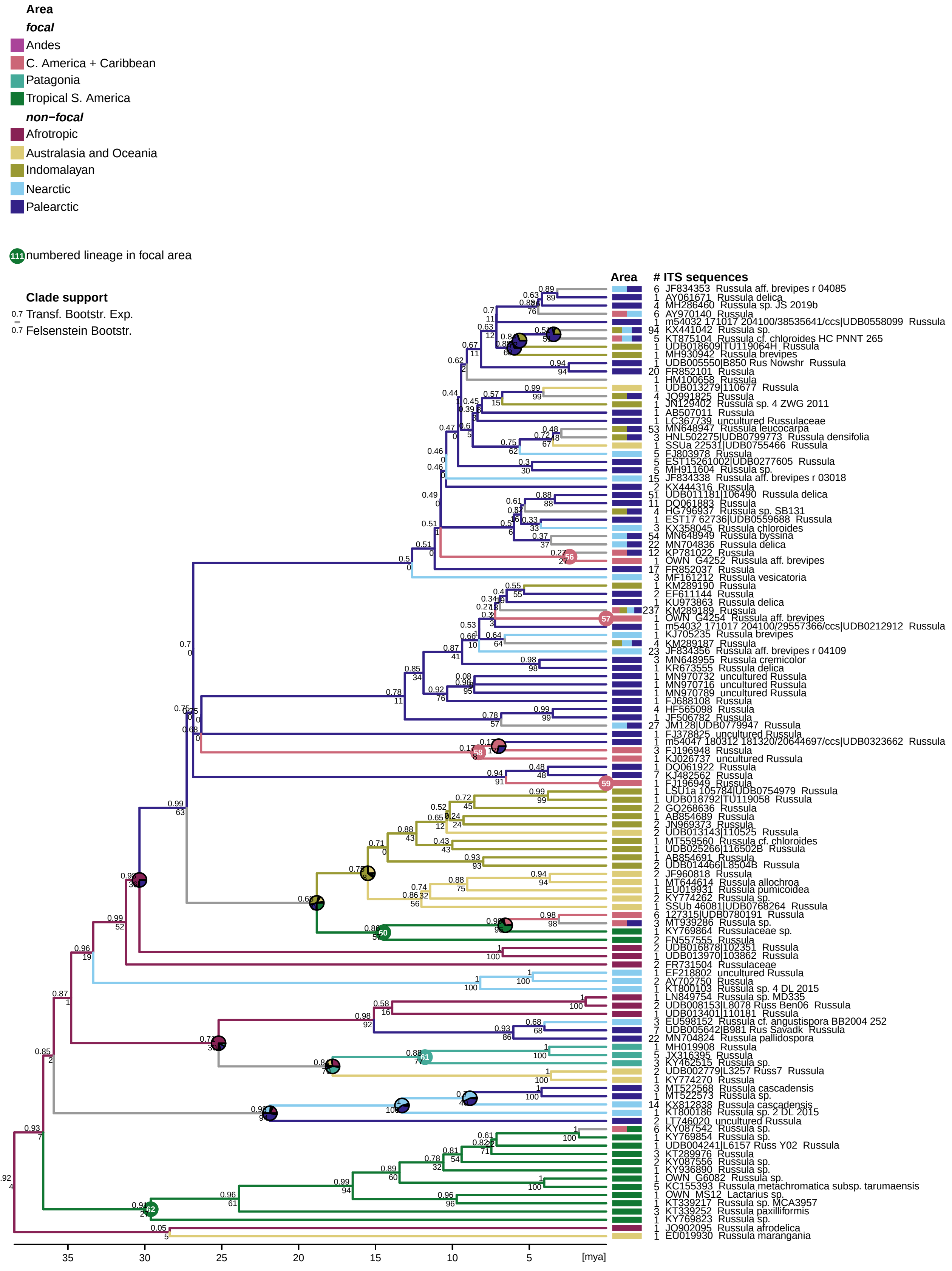
Area
focal
Andes
C. America + Caribbean
Patagonia
Tropical S. America
non−focal
Afrotropic
Australasia and Oceania
Indomalayan
Nearctic
Palearctic
111 numbered lineage in focal area
Clade support
0.7 Transf. Bootstr. Exp.
0.7 Felsenstein Bootstr.
Area
ITS sequences
6 JF834353 Russula aff. brevipes r 04085
1 AY061671 Russula delica
4 MH286460 Russula sp. JS 2019b
6 AY970140 Russula
1 m54032 171017 204100/38535641/ccs|UDB0558099 Russula
94 KX441042 Russula sp.
5 KT875104 Russula cf. chloroides HC PNNT 265
1 UDB018609|TU119064H Russula
1 MH930942 Russula brevipes
1 UDB005550|B850 Rus Nowshr Russula
20 FR852101 Russula
1 HM100658 Russula
1 UDB013279|110677 Russula
4 JQ991825 Russula
1 JN129402 Russula sp. 4 ZWG 2011
1 AB507011 Russula
1 LC367739 uncultured Russulaceae
53 MN648947 Russula leucocarpa
3 HNL502275|UDB0799773 Russula densifolia
1 SSUa 22531|UDB0755466 Russula
5 FJ803978 Russula
5 EST15261002|UDB0277605 Russula
5 MH911604 Russula sp.
15 JF834338 Russula aff. brevipes r 03018
2 KX444316 Russula
51 UDB011181|106490 Russula delica
11 DQ061883 Russula
4 HG796937 Russula sp. SB131
1 EST17 62736|UDB0559688 Russula
3 KX358045 Russula chloroides
54 MN648949 Russula byssina
22 MN704836 Russula delica
12 KP781022 Russula
1 OWN G4252 Russula aff. brevipes
17 FR852037 Russula
3 MF161212 Russula vesicatoria
1 KM289190 Russula
2 EF611144 Russula
1 KU973863 Russula delica
237 KM289189 Russula
1 OWN G4254 Russula aff. brevipes
1 m54032 171017 204100/29557366/ccs|UDB0212912 Russula
1 KJ705235 Russula brevipes
4 KM289187 Russula
23 JF834356 Russula aff. brevipes r 04109
3 MN648955 Russula cremicolor
1 KR673555 Russula delica
1 MN970732 uncultured Russula
1 MN970716 uncultured Russula
1 MN970789 uncultured Russula
1 FJ688108 Russula
4 HF565098 Russula
1 JF506782 Russula
27 JM128|UDB0779947 Russula
1 FJ378825 uncultured Russula
1 m54047 180312 181320/20644697/ccs|UDB0323662 Russula
3 FJ196948 Russula
1 KJ026737 uncultured Russula
1 DQ061922 Russula
7 KJ482562 Russula
1 FJ196949 Russula
1 LSU1a 105784|UDB0754979 Russula
1 UDB018792|TU119058 Russula
2 GQ268636 Russula
1 AB854689 Russula
2 JN969373 Russula
2 UDB013143|110525 Russula
1 MT559560 Russula cf. chloroides
1 UDB025266|1165028 Russula
1 AB854691 Russula
2 UDB014466|L8504B Russula
2 JF960818 Russula
1 MT644614 Russula allochroa
1 EU019931 Russula pumicoidea
2 KY774262 Russula sp.
1 SSUb 46081|UDB0768264 Russula
6 127315|UDB0780191 Russula
3 MT939286 Russula sp.
1 KY769864 Russulaceae sp.
2 FN557555 Russula
2 UDB016878|102351 Russula
1 UDB013970|103862 Russula
2 FR731504 Russulaceae
1 EF218802 uncultured Russula
2 AY702750 Russula
1 KT800103 Russula sp. 4 DL 2015
1 LN849754 Russula sp. MD335
2 UDB008153|L8078 Russ Ben06 Russula
1 UDB013401|110181 Russula
3 EU598152 Russula cf. angustispora BB2004 252
7 UDB005642|B981 Rus Savadk Russula
22 MN704824 Russula pallidospora
1 MH019908 Russula
5 JX316395 Russula
3 KY462515 Russula sp.
2 UDB002779|L3257 Russ7 Russula
1 KY774270 Russula
3 MT522568 Russula cascadensis
1 MT522573 Russula sp.
14 KX812838 Russula cascadensis
1 KT800186 Russula sp. 2 DL 2015
2 LT746020 uncultured Russula
6 KY087542 Russula sp.
1 KY769854 Russula sp.
1 UDB004241|L6157 Russ Y02 Russula
3 KT289976 Russula
2 KY087556 Russula sp.
1 KY936890 Russula sp.
1 OWN G6082 Russula sp.
5 KC155393 Russula metachromatica subsp. tarumaensis
1 OWN MS12 Lactarius sp.
1 KT339217 Russula sp. MCA3957
3 KT339252 Russula paxilliformis
1 KY769823 Russula sp.
1 JQ902095 Russula afrodelica
1 EU019930 Russula marangania
56
57
58
59
60
61
62
35
30
25
20
15
10
5
[mya]

Figure S3 s

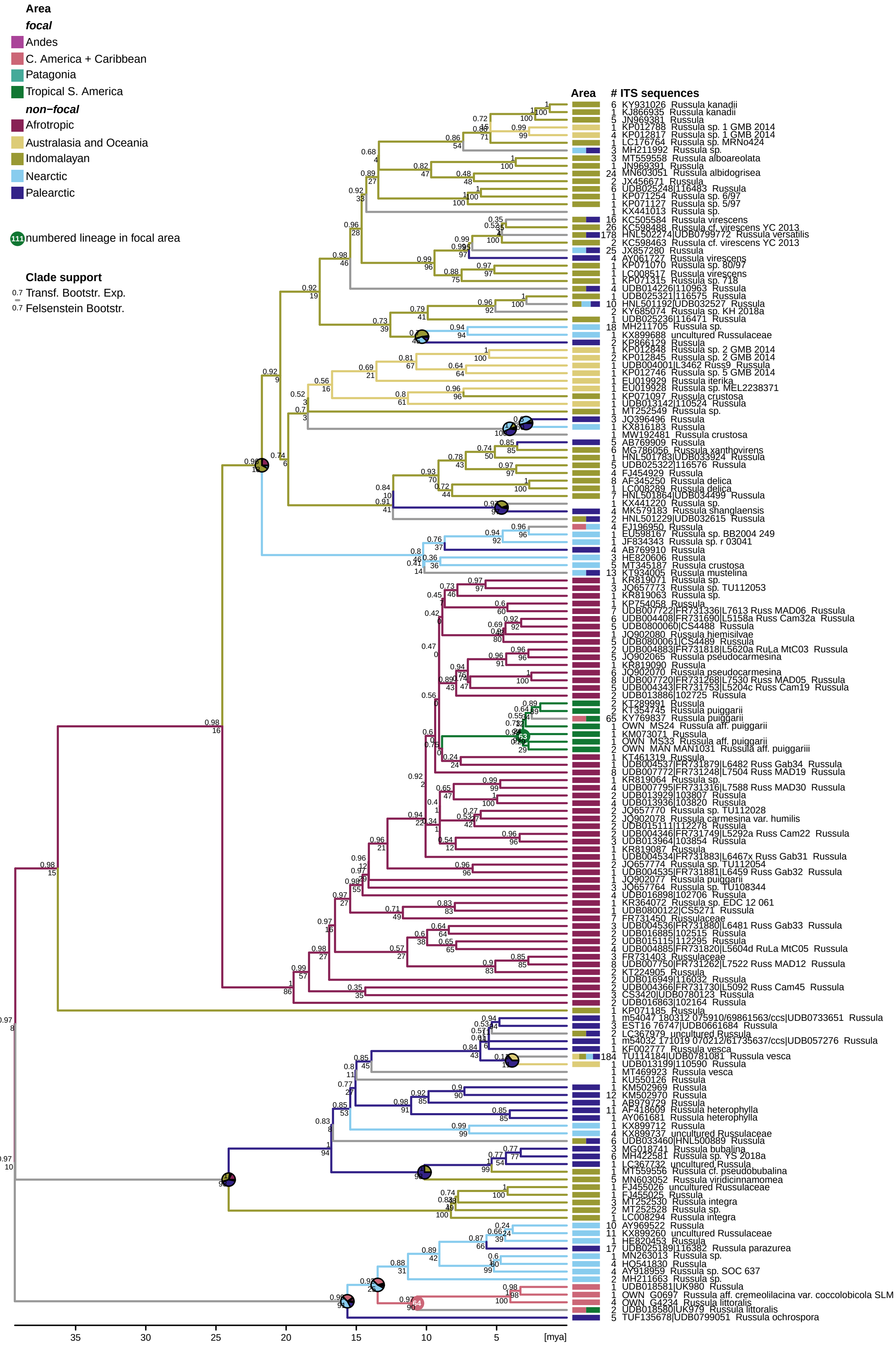

Area
focal
Andes
C. America + Caribbean
Patagonia
Tropical S. America
non−focal
Afrotropic
Australasia and Oceania
Indomalayan
Nearctic
Palearctic
111 numbered lineage in focal area
Clade support
0.7 Transf. Bootstr. Exp.
0.7 Felsenstein Bootstr.
Area
ITS sequences
6 KY931026 Russula kanadii
1 KJ866935 Russula kanadii
5 JN969381 Russula
1 KP012788 Russula sp. 1 GMB 2014
4 KP012817 Russula sp. 1 GMB 2014
1 LC176764 Russula sp. MRNo424
3 MH211992 Russula sp.
3 MT559558 Russula alboareolata
1 JN969391 Russula
24 MN603051 Russula albidogrisea
2 JX456671 Russula
6 UDB025248|116483 Russula
1 KP071254 Russula sp. 6/97
1 KP071127 Russula sp. 5/97
1 KX441013 Russula sp.
16 KC505584 Russula virescens
26 KC598488 Russula cf. virescens YC 2013
178 HNL502274|UDB0799772 Russula versatilis
2 KC598463 Russula cf. virescens YC 2013
25 JX857280 Russula
4 AY061727 Russula virescens
1 KP071070 Russula sp. 80/97
1 LC008517 Russula virescens
1 KP071315 Russula sp. 718
4 UDB014226|110963 Russula
1 UDB025321|116575 Russula
10 HNL501192|UDB032527 Russula
2 KY685074 Russula sp. KH 2018a
1 UDB025236|116471 Russula
18 MH211705 Russula sp.
1 KX899688 uncultured Russulaceae
2 KP866129 Russula
1 KP012848 Russula sp. 2 GMB 2014
2 KP012845 Russula sp. 2 GMB 2014
1 UDB004001|L3462 Russ9 Russula
1 KP012746 Russula sp. 5 GMB 2014
1 EU019929 Russula iterika
1 EU019928 Russula sp. MEL2238371
1 KP071097 Russula crustosa
1 UDB013142|110524 Russula
1 MT252549 Russula sp.
3 JQ396496 Russula
1 KX816183 Russula
1 MW192481 Russula crustosa
5 AB769909 Russula
6 MG786056 Russula xanthovirens
1 HNL501783|UDB033924 Russula
5 UDB025322|116576 Russula
4 FJ454929 Russula
8 AF345250 Russula delica
1 LC008289 Russula delica
7 HNL501864|UDB034499 Russula
1 KX441220 Russula sp.
4 MK579183 Russula shanglaensis
2 HNL501229|UDB032615 Russula
4 FJ196950 Russula
1 EU598167 Russula sp. BB2004 249
1 JF834343 Russula sp. r 03041
4 AB769910 Russula
3 HE820606 Russula
5 MT345187 Russula crustosa
13 KT934005 Russula mustelina
1 KR819071 Russula sp.
3 JQ657773 Russula sp. TU112053
1 KR819063 Russula sp.
1 KP754058 Russula
7 UDB007722|FR731336|L7613 Russ MAD06 Russula
6 UDB004408|FR731690|L5158a Russ Cam32a Russula
5 UDB0800060|CS4488 Russula
1 JQ902080 Russula hiemisilvae
5 UDB0800061|CS4489 Russula
2 UDB004883|FR731818|L5620a RuLa MtC03 Russula
5 JQ902065 Russula pseudocarmesina
1 KR819090 Russula
6 JQ902070 Russula pseudocarmesina
8 UDB007720|FR731268|L7530 Russ MAD05 Russula
5 UDB004343|FR731753|L5204c Russ Cam19 Russula
2 UDB013886|102725 Russula
2 KT289991 Russula
2 KT354745 Russula puiggarii
65 KY769837 Russula puiggarii
1 OWN_MS24 Russula aff. puiggarii
1 KM073071 Russula
1 OWN_MS33 Russula aff. puiggarii
2 OWN_MAN MAN1031 Russula aff. puiggariii
1 KT461319 Russula
1 UDB004537|FR731879|L6482 Russ Gab34 Russula
8 UDB007772|FR731248|L7504 Russ MAD19 Russula
1 KR819064 Russula sp.
4 UDB007795|FR731316|L7588 Russ MAD30 Russula
2 UDB013929|103807 Russula
4 UDB013936|103820 Russula
2 JQ657770 Russula sp. TU112028
2 JQ902078 Russula carmesina var. humilis
2 UDB015111|112278 Russula
2 UDB004346|FR731749|L5292a Russ Cam22 Russula
3 UDB013964|103854 Russula
1 KR819087 Russula
1 UDB004534|FR731883|L6467x Russ Gab31 Russula
2 JQ657774 Russula sp. TU112054
1 UDB004535|FR731881|L6459 Russ Gab32 Russula
1 JQ902077 Russula puiggarii
3 JQ657764 Russula sp. TU108344
4 UDB016898|102706 Russula
1 KR364072 Russula sp. EDC 12 061
1 UDB0800122|CS5271 Russula
7 FR731450 Russulaceae
3 UDB004536|FR731880|L6481 Russ Gab33 Russula
2 UDB016885|102515 Russula
2 UDB015115|112295 Russula
4 UDB004885|FR731820|L5604d RuLa MtC05 Russula
3 FR731403 Russulaceae
8 UDB007750|FR731262|L7522 Russ MAD12 Russula
2 KT224905 Russula
2 UDB016949|116032 Russula
2 UDB004366|FR731730|L5092 Russ Cam45 Russula
3 CS3420|UDB0780123 Russula
2 UDB016863|102164 Russula
1 KP071185 Russula
1 m54047 180312 075910/69861563/ccs|UDB0733651 Russula
3 EST16 76747|UDB0661684 Russula
2 LC367979 uncultured Russula
1 m54032 171019 070212/61735637/ccs|UDB057276 Russula
1 KF002777 Russula vesca
184 TU114184|UDB0781081 Russula vesca
1 UDB013199|110590 Russula
1 MT469923 Russula vesca
1 KU550126 Russula
1 KM502969 Russula
12 KM502970 Russula
1 AB979729 Russula
11 AF418609 Russula heterophylla
1 AY061681 Russula heterophylla
1 KX899712 Russula
4 KX899737 uncultured Russulaceae
6 UDB033460|HNL500889 Russula
3 MG018741 Russula bubalina
6 MH422581 Russula sp. YS 2018a
1 LC367732 uncultured Russula
1 MT559556 Russula cf. pseudobubalina
5 MN603052 Russula viridicinnamomea
1 FJ455026 uncultured Russulaceae
1 FJ455025 Russula
3 MT252530 Russula integra
2 MT252528 Russula sp.
1 LC008294 Russula integra
10 AY969522 Russula
11 KX899260 uncultured Russulaceae
1 HE820453 Russula
17 UDB025189|116382 Russula parazurea
1 MN263013 Russula sp.
4 HQ541830 Russula
4 AY918959 Russula sp. SOC 637
2 MH211663 Russula sp.
1 UDB018581|UK980 Russula
1 OWN_G0697 Russula aff. cremeolilacina var. coccolobicola SLM
4 OWN_G4234 Russula littoralis
2 UDB018580|UK979 Russula littoralis
5 TUF135678|UDB0799051 Russula ochrospora
35
30
25
20
15
10
5
[mya]

Figure S3 t

Area

focal

Andes

C. America + Caribbean

Patagonia

Tropical S. America

non−focal

Afrotropic

Australasia and Oceania

Indomalayan

Nearctic

Palearctic

111 numbered lineage in focal area

Clade support

0.7 Transf. Bootstr. Exp.

0.7 Felsenstein Bootstr.

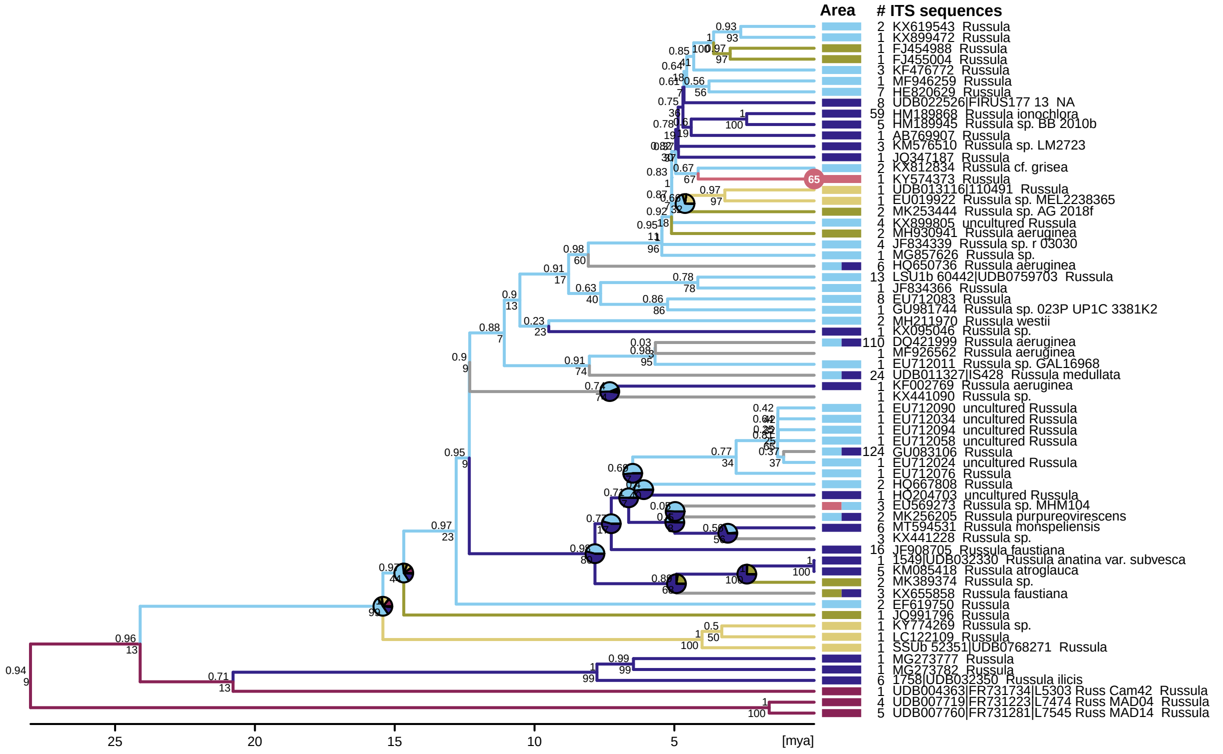

Figure S3 u

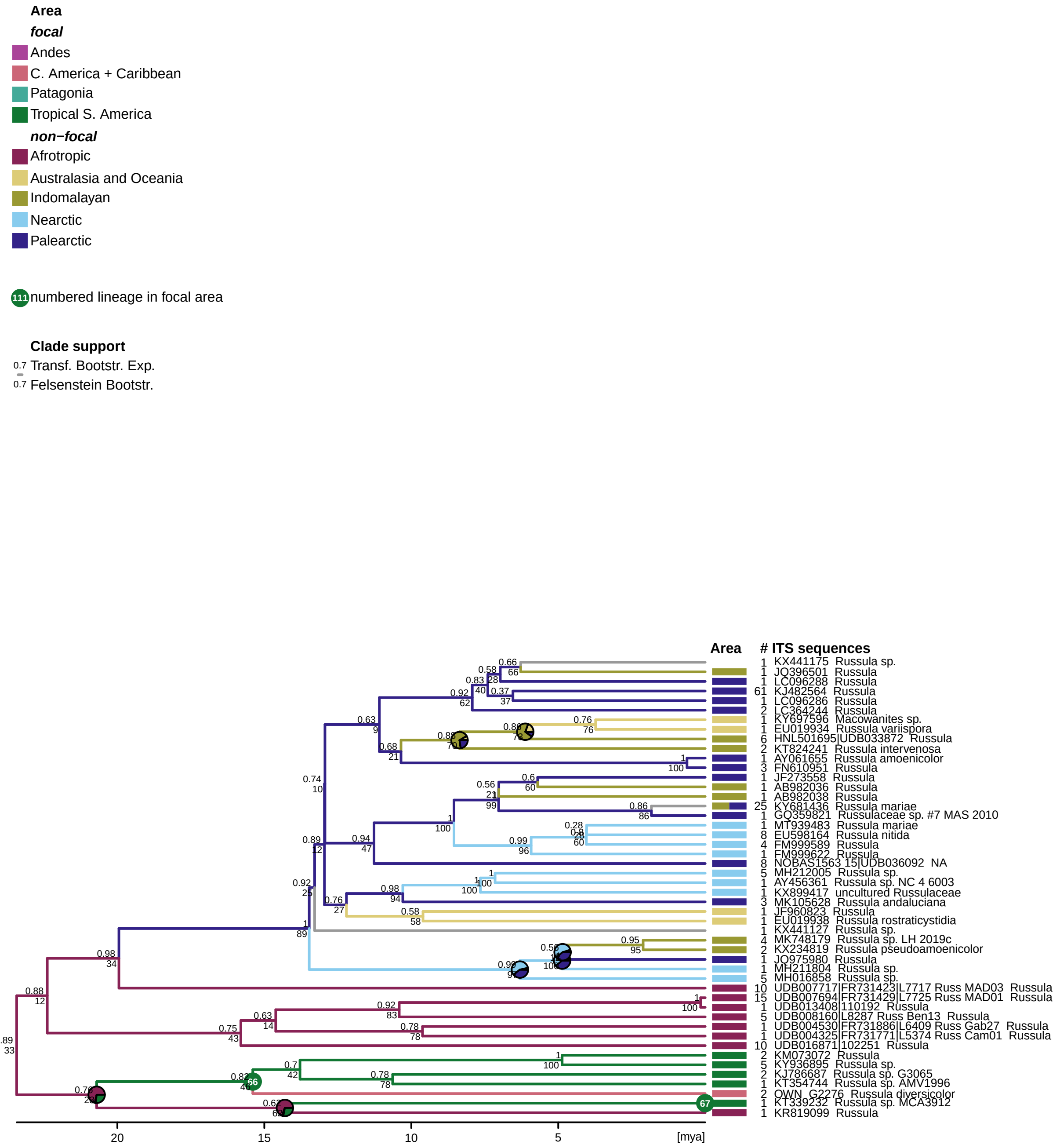

Area
focal
Andes
C. America + Caribbean
Patagonia
Tropical S. America
non−focal
Afrotropic
Australasia and Oceania
Indomalayan
Nearctic
Palearctic
111 numbered lineage in focal area
Clade support
0.7 Transf. Bootstr. Exp.
0.7 Felsenstein Bootstr.
Area
ITS sequences
1 KX441175 Russula sp.
1 JQ396501 Russula
1 LC096288 Russula
61 KJ482564 Russula
1 LC096286 Russula
2 LC364244 Russula
1 KY697596 Macowanites sp.
1 EU019934 Russula variispora
6 HNL501695|UDB033872 Russula
2 KT824241 Russula intervenosa
1 AY061655 Russula amoenicolor
3 FN610951 Russula
1 JF273558 Russula
1 AB982036 Russula
1 AB982038 Russula
25 KY681436 Russula mariae
1 GQ359821 Russulaceae sp. #7 MAS 2010
1 MT939483 Russula mariae
8 EU598164 Russula nitida
4 FM999589 Russula
1 FM999622 Russula
8 NOBAS1563 15|UDB036092 NA
5 MH212005 Russula sp.
1 AY456361 Russula sp. NC 4 6003
1 KX899417 uncultured Russulaceae
3 MK105628 Russula andaluciana
1 JF960823 Russula
1 EU019938 Russula rostraticystidia
1 KX441127 Russula sp.
4 MK748179 Russula sp. LH 2019c
2 KX234819 Russula pseudoamoenicolor
1 JQ975980 Russula
1 MH211804 Russula sp.
5 MH016858 Russula sp.
10 UDB007717|FR731423|L7717 Russ MAD03 Russula
15 UDB007694|FR731429|L7725 Russ MAD01 Russula
1 UDB013408|110192 Russula
5 UDB008160|L8287 Russ Ben13 Russula
1 UDB004530|FR731886|L6409 Russ Gab27 Russula
1 UDB004325|FR731771|L5374 Russ Cam01 Russula
10 UDB016871|102251 Russula
2 KM073072 Russula
5 KY936895 Russula sp.
2 KJ786687 Russula sp. G3065
1 KT354744 Russula sp. AMV1996
2 OWN G2276 Russula diversicolor
1 KT339232 Russula sp. MCA3912
1 KR819099 Russula
66
67
20
15
10
5
[mya]

Figure S3 v

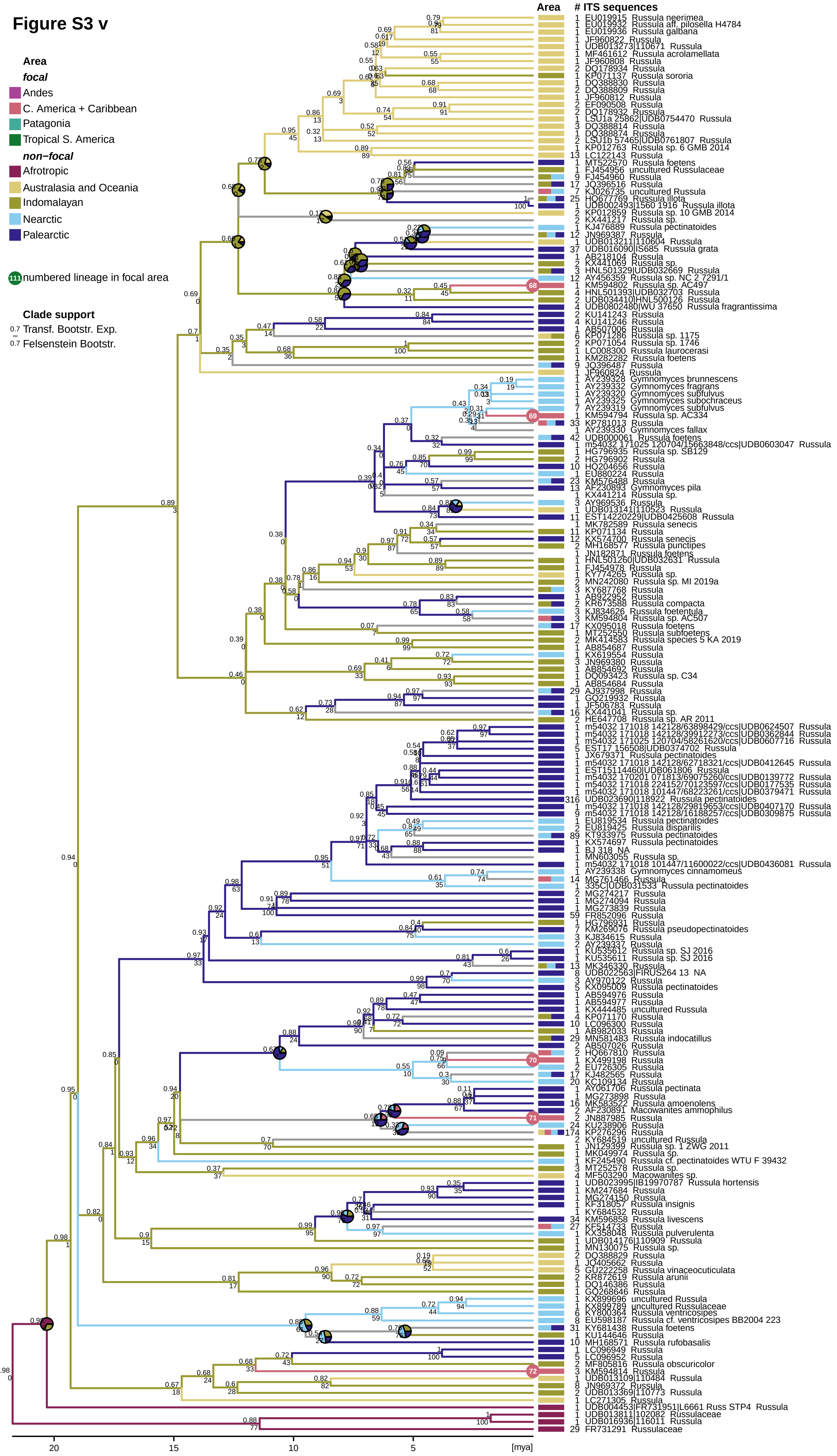
Area
focal
Andes
C. America + Caribbean
Patagonia
Tropical S. America
non–focal
Afrotropic
Australasia and Oceania
Indomalayan
Nearctic
Palearctic
111 numbered lineage in focal area
Clade support
0.7 Transf. Bootstr. Exp.
0.7 Felsenstein Bootstr.
Area
ITS sequences
1 EU019915 Russula neerimea
1 EU019932 Russula aff. pilosella H4784
1 EU019936 Russula galbana
1 JF960822 Russula
1 UDB013273|110671 Russula
1 MF461612 Russula acrolamellata
1 JF960808 Russula
2 DQ178934 Russula
1 KP071137 Russula sororia
1 DQ388830 Russula
2 DQ388809 Russula
1 JF960812 Russula
2 EF090508 Russula
2 DQ178932 Russula
1 LSU1a 25862|UDB0754470 Russula
3 DQ388814 Russula
1 DQ388874 Russula
2 LSU1b 57465|UDB0761807 Russula
1 KP012763 Russula sp. 6 GMB 2014
13 LC122143 Russula
1 MT522570 Russula foetens
1 FJ454956 uncultured Russulaceae
9 FJ454960 Russula
17 JQ396516 Russula
7 KJ026735 uncultured Russula
25 HQ677769 Russula illota
1 UDB002493|1560 1916 Russula illota
2 KP012859 Russula sp. 10 GMB 2014
2 KX441217 Russula sp.
1 KJ476889 Russula pectinatoides
12 JN969387 Russula
1 UDB013211|110604 Russula
37 UDB016090|IS685 Russula grata
1 AB218104 Russula
2 KX441069 Russula sp.
3 HNL501329|UDB032669 Russula
12 AY456359 Russula sp. NC 2 7291/1
68
1 KM594802 Russula sp. AC497
4 HNL501393|UDB032703 Russula
2 UDB034410|HNL500126 Russula
4 UDB0802480|WU 37650 Russula fragrantissima
2 KU141243 Russula
4 KU141246 Russula
1 AB507006 Russula
6 KP071286 Russula sp. 1175
2 KP071054 Russula sp. 1746
1 LC008300 Russula laurocerasi
1 KM282282 Russula foetens
9 JQ396487 Russula
1 JF960824 Russula
1 AY239328 Gymnomyces brunnescens
1 AY239332 Gymnomyces fragrans
1 AY239320 Gymnomyces subfulvus
1 AY239325 Gymnomyces subochraceus
7 AY239319 Gymnomyces subfulvus
69
1 KM594794 Russula sp. AC334
33 KP781013 Russula
1 AY239330 Gymnomyces fallax
42 UDB000061 Russula foetens
1 m54032 171025 120704/15663848/ccs|UDB0603047 Russula
1 HG796935 Russula sp. SB129
2 HG796902 Russula
10 HQ204656 Russula
1 EU880224 Russula
23 KM576488 Russula
13 AF230893 Gymnomyces pila
1 KX441214 Russula sp.
3 AY969536 Russula
1 UDB013141|110523 Russula
11 EST14220229|UDB0425608 Russula
1 MK782589 Russula senecis
11 KP071134 Russula
12 KX574700 Russula senecis
2 MH168577 Russula punctipes
1 JN182871 Russula foetens
1 HNL501260|UDB032631 Russula
1 FJ454978 Russula
1 KY774265 Russula sp.
2 MN242080 Russula sp. MI 2019a
3 KY687768 Russula
1 AB922952 Russula
2 KR673588 Russula compacta
3 KJ834626 Russula foetentula
3 KM594804 Russula sp. AC507
17 KX095018 Russula foetens
1 MT252550 Russula subfoetens
2 MK414583 Russula species 5 KA 2019
1 AB854687 Russula
1 KX619554 Russula
3 JN969380 Russula
1 AB854692 Russula
1 DQ093423 Russula sp. C34
1 AB854684 Russula
29 AJ937998 Russula
1 GQ219932 Russula
1 JF506783 Russula
16 KX441041 Russula sp.
2 HE647708 Russula sp. AR 2011
1 m54032 171018 142128/63898429/ccs|UDB0624507 Russula
1 m54032 171018 142128/39912273/ccs|UDB0362844 Russula
1 m54032 171025 120704/58261620/ccs|UDB0607716 Russula
5 EST17 156508|UDB0374702 Russula
1 JX679371 Russula pectinatoides
1 m54032 171018 142128/62718321/ccs|UDB0412645 Russula
1 EST15114460|UDB061806 Russula
1 m54032 170201 071813/69075260/ccs|UDB0139772 Russula
1 m54032 171018 224152/70123597/ccs|UDB0177535 Russula
1 m54032 171018 101447/68223261/ccs|UDB0379471 Russula
316 UDB023690|118922 Russula pectinatoides
1 m54032 171018 142128/29819653/ccs|UDB0407170 Russula
9 m54032 171018 142128/16188257/ccs|UDB0309875 Russula
1 EU819534 Russula pectinatoides
2 EU819425 Russula disparilis
89 KT933975 Russula pectinatoides
1 KX574697 Russula pectinatoides
1 BJ 318 NA
1 MN603055 Russula sp.
1 m54032 171018 101447/11600022/ccs|UDB0436081 Russula
1 AY239338 Gymnomyces cinnamomeus
14 MG761466 Russula
1 335C|UDB031533 Russula pectinatoides
2 MG274217 Russula
1 MG274094 Russula
1 MG273839 Russula
59 FR852096 Russula
1 HG796931 Russula
7 KM269076 Russula pseudopectinatoides
3 KJ834615 Russula
2 AY239337 Russula
1 KU535612 Russula sp. SJ 2016
1 KU535611 Russula sp. SJ 2016
13 MK346330 Russula
8 UDB022563|FIRUS264 13 NA
3 AY970122 Russula
5 KX095009 Russula pectinatoides
1 AB594976 Russula
1 AB594977 Russula
1 KX444485 uncultured Russula
4 KP071170 Russula
10 LC096300 Russula
1 AB982033 Russula
29 MN581483 Russula indocatillus
2 AB507026 Russula
2 HQ667810 Russula
70
1 KX499198 Russula
2 EU726305 Russula
17 KJ482565 Russula
20 KC109134 Russula
1 AY061706 Russula pectinata
1 MG273898 Russula
16 MK583522 Russula amoenolens
2 AF230891 Macowanites ammophilus
71
1 JN887985 Russula
24 KU238906 Russula
174 KP276296 Russula
2 KY684519 uncultured Russula
1 JN129399 Russula sp. 1 ZWG 2011
1 MK049974 Russula sp.
1 KF245490 Russula cf. pectinatoides WTU F 39432
3 MT252578 Russula sp.
4 MF503290 Macowanites sp.
1 UDB023995|IB19970787 Russula hortensis
1 KM247684 Russula
1 MG274150 Russula
1 KF318057 Russula insignis
1 KY684532 Russula
34 KM596858 Russula livescens
27 KF514733 Russula
1 KX358048 Russula pulverulenta
1 UDB014176|110909 Russula
1 MN130075 Russula sp.
2 DQ388829 Russula
1 JQ405662 Russula
5 GU222258 Russula vinaceocuticulata
2 KR872619 Russula arunii
1 DQ146386 Russula
1 GQ268646 Russula
1 KX899696 uncultured Russula
1 KX899789 uncultured Russulaceae
6 KY800364 Russula ventricosipes
8 EU598187 Russula cf. ventricosipes BB2004 223
31 KY681438 Russula foetens
1 KU144646 Russula
10 MH168571 Russula rufobasalis
1 LC096949 Russula
5 LC096952 Russula
2 MF805816 Russula obscuricolor
72
3 KM594814 Russula
1 UDB013109|110484 Russula
8 JN969372 Russula
2 UDB013369|110773 Russula
1 LC271305 Russula
1 UDB004453|FR731951|L6661 Russ STP4 Russula
1 UDB013811|102082 Russulaceae
1 UDB016936|116011 Russula
29 FR731291 Russulaceae
20
15
10
5
[mya]

Figure S3 w

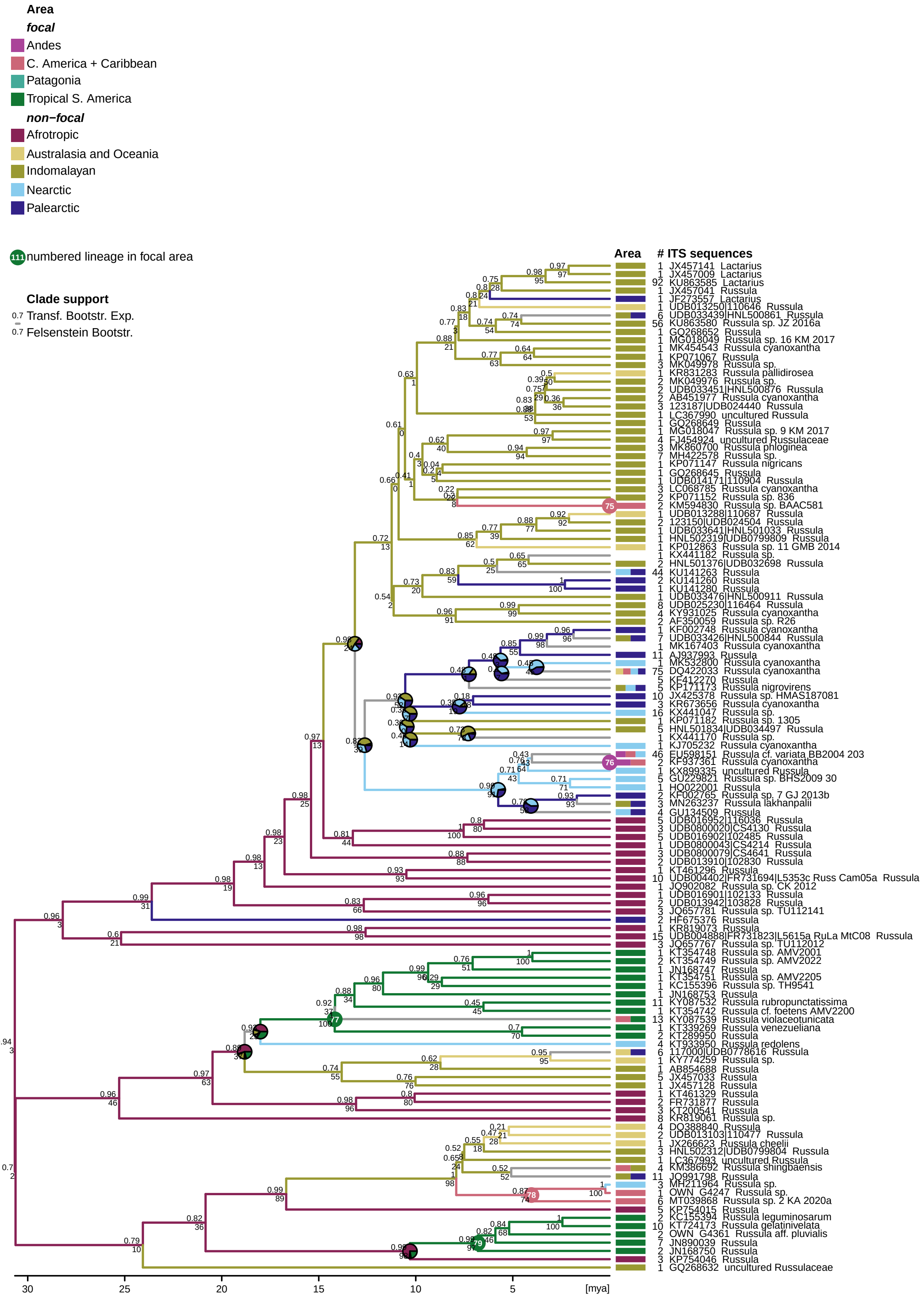

Area
focal
Andes
C. America + Caribbean
Patagonia
Tropical S. America
non−focal
Afrotropic
Australasia and Oceania
Indomalayan
Nearctic
Palearctic
numbered lineage in focal area
Clade support
Transf. Bootstr. Exp.
Felsenstein Bootstr.
Area
ITS sequences
1 JX457141 Lactarius
1 JX457009 Lactarius
92 KU863585 Lactarius
1 JX457041 Russula
1 JF273557 Lactarius
1 UDB013250|110646 Russula
6 UDB033439|HNL500861 Russula
56 KU863580 Russula sp. JZ 2016a
1 GQ268652 Russula
1 MG018049 Russula sp. 16 KM 2017
1 MK454543 Russula cyanoxantha
1 KP071067 Russula
3 MK049978 Russula sp.
1 KR831283 Russula pallidirosea
2 MK049976 Russula sp.
2 UDB033451|HNL500876 Russula
2 AB451977 Russula cyanoxantha
3 123187|UDB024440 Russula
1 LC367990 uncultured Russula
1 GQ268649 Russula
1 MG018047 Russula sp. 9 KM 2017
4 FJ454924 uncultured Russulaceae
3 MK860700 Russula phloginea
7 MH422578 Russula sp.
1 KP071147 Russula nigricans
1 GQ268645 Russula
1 UDB014171|110904 Russula
3 LC068785 Russula cyanoxantha
2 KP071152 Russula sp. 836
2 KM594830 Russula sp. BAAC581
1 UDB013288|110687 Russula
2 123150|UDB024504 Russula
1 UDB033641|HNL501033 Russula
1 HNL502319|UDB0799809 Russula
1 KP012863 Russula sp. 11 GMB 2014
1 KX441182 Russula sp.
2 HNL501376|UDB032698 Russula
44 KU141263 Russula
2 KU141260 Russula
1 KU141280 Russula
1 UDB033476|HNL500911 Russula
8 UDB025230|116464 Russula
4 KY931025 Russula cyanoxantha
2 AF350059 Russula sp. R26
1 KF002748 Russula cyanoxantha
7 UDB033426|HNL500844 Russula
1 MK167403 Russula cyanoxantha
11 AJ937993 Russula
1 MK532800 Russula cyanoxantha
75 DQ422033 Russula cyanoxantha
5 KF412270 Russula
5 KP171173 Russula nigrovirens
10 JX425378 Russula sp. HMAS187081
3 KR673656 Russula cyanoxantha
16 KX441047 Russula sp.
1 KP071182 Russula sp. 1305
5 HNL501834|UDB034497 Russula
1 KX441170 Russula sp.
1 KJ705232 Russula cyanoxantha
46 EU598151 Russula cf. variata BB2004 203
2 KF937361 Russula cyanoxantha
1 KX899335 uncultured Russula
5 GU229821 Russula sp. BHS2009 30
1 HQ022001 Russula
2 KF002765 Russula sp. 7 GJ 2013b
3 MN263237 Russula lakhanpalii
4 GU134509 Russula
5 UDB016952|116036 Russula
3 UDB0800020|CS4130 Russula
5 UDB016902|102485 Russula
1 UDB0800043|CS4214 Russula
3 UDB0800079|CS4641 Russula
2 UDB013910|102830 Russula
1 KT461296 Russula
10 UDB004402|FR731694|L5353c Russ Cam05a Russula
1 JQ902082 Russula sp. CK 2012
1 UDB016901|102133 Russula
2 UDB013942|103828 Russula
3 JQ657781 Russula sp. TU112141
2 HF675376 Russula
1 KR819073 Russula
15 UDB004888|FR731823|L5615a RuLa MtC08 Russula
3 JQ657767 Russula sp. TU112012
1 KT354748 Russula sp. AMV2001
2 KT354749 Russula sp. AMV2022
1 JN168747 Russula
1 KT354751 Russula sp. AMV2205
1 KC155396 Russula sp. TH9541
1 JN168753 Russula
11 KY087532 Russula rubropunctatissima
1 KT354742 Russula cf. foetens AMV2200
13 KY087539 Russula violaceotunicata
1 KT339269 Russula venezueliana
2 KT289950 Russula
4 KT933950 Russula redolens
6 117000|UDB0778616 Russula
1 KY774259 Russula sp.
1 AB854688 Russula
5 JX457033 Russula
1 JX457128 Russula
1 KT461329 Russula
2 FR731877 Russula
3 KT200541 Russula
8 KR819061 Russula sp.
4 DQ388840 Russula
2 UDB013103|110477 Russula
1 JX266623 Russula cheelii
3 HNL502312|UDB0799804 Russula
1 LC367993 uncultured Russula
4 KM386692 Russula shingbaensis
11 JQ991798 Russula
3 MH211964 Russula sp.
1 OWN G4247 Russula sp.
6 MT039868 Russula sp. 2 KA 2020a
5 KP754015 Russula
2 KC155394 Russula leguminosarum
10 KT724173 Russula gelatinivelata
2 OWN G4361 Russula aff. pluvialis
7 JN890039 Russula
2 JN168750 Russula
3 KP754046 Russula
1 GQ268632 uncultured Russulaceae
75
76
77
78
79
30
25
20
15
10
5
[mya]

Figure S3 x

Area

focal

Andes

C. America + Caribbean

Patagonia

Tropical S. America

non−focal

Afrotropic

Australasia and Oceania

Indomalayan

Nearctic

Palearctic

111 numbered lineage in focal area

Clade support

0.7 Transf. Bootstr. Exp.

0.7 Felsenstein Bootstr.

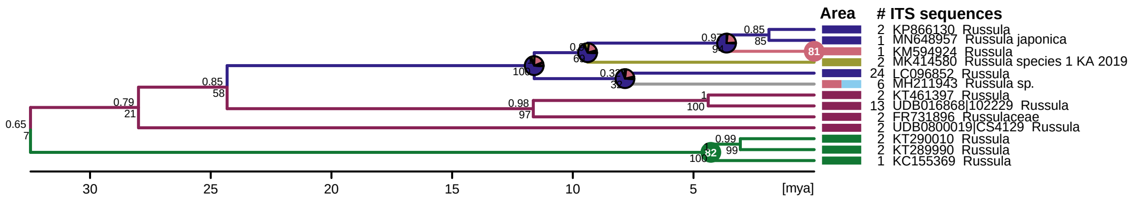

Figure S3 y

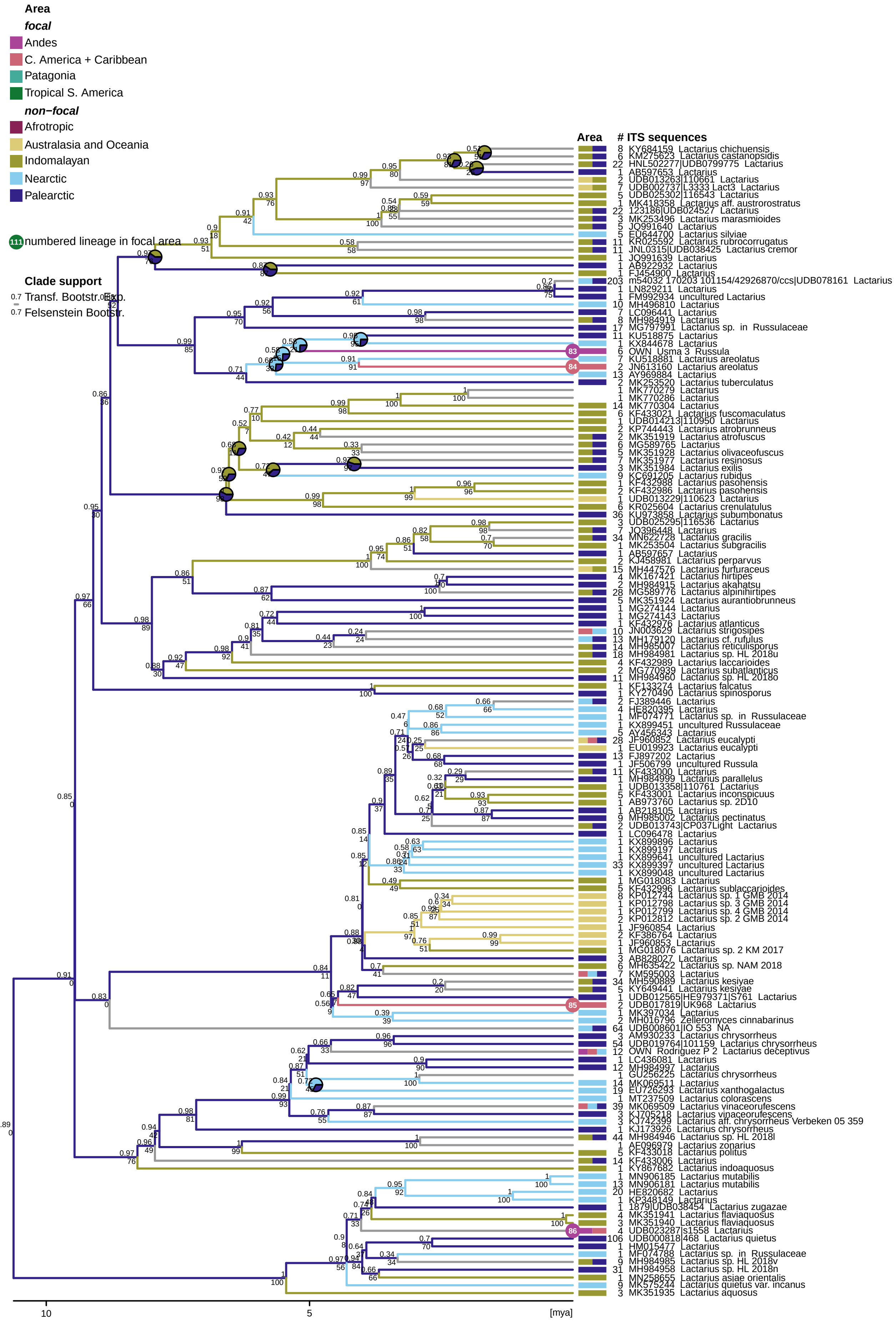
Area
focal
Andes
C. America + Caribbean
Patagonia
Tropical S. America
non−focal
Afrotropic
Australasia and Oceania
Indomalayan
Nearctic
Palearctic
111 numbered lineage in focal area
Clade support
0.7 Transf. Bootstr. Exp.
0.7 Felsenstein Bootstr.
Area
ITS sequences
8 KY684159 Lactarius chichuensis
6 KM275623 Lactarius castanopsidis
22 HNL502277|UDB0799775 Lactarius
1 AB597653 Lactarius
2 UDB013263|110661 Lactarius
7 UDB002737|L3333 Lact3 Lactarius
5 UDB025302|116543 Lactarius
1 MK418358 Lactarius aff. austrorostratus
22 123186|UDB024527 Lactarius
3 MK253496 Lactarius marasmioides
5 JQ991640 Lactarius
5 EU644700 Lactarius silviae
11 KR025592 Lactarius rubrocorrugatus
11 JNL0315|UDB038425 Lactarius cremor
1 JQ991639 Lactarius
1 AB922932 Lactarius
1 FJ454900 Lactarius
203 m54032 170203 101154/42926870/ccs|UDB078161 Lactarius
1 LN829211 Lactarius
1 FM992934 uncultured Lactarius
10 MH496810 Lactarius
7 LC096441 Lactarius
8 MH984919 Lactarius
17 MG797991 Lactarius sp. in Russulaceae
11 KU518875 Lactarius
1 KX844678 Lactarius
83
6 OWN Usma 3 Russula
7 KU518881 Lactarius areolatus
84
2 JN613160 Lactarius areolatus
13 AY969884 Lactarius
2 MK253520 Lactarius tuberculatus
1 MK770279 Lactarius
1 MK770286 Lactarius
14 MK770304 Lactarius
6 KF433021 Lactarius fuscomaculatus
1 UDB014213|110950 Lactarius
2 KP744443 Lactarius atrobrunneus
2 MK351919 Lactarius atrofuscus
6 MG589765 Lactarius
5 MK351928 Lactarius olivaceofuscus
7 MK351977 Lactarius resinosus
3 MK351984 Lactarius exilis
9 KC691205 Lactarius rubidus
1 KF432988 Lactarius pasohensis
2 KF432986 Lactarius pasohensis
1 UDB013229|110623 Lactarius
6 KR025604 Lactarius crenulatulus
36 KU973858 Lactarius subumbonatus
3 UDB025295|116536 Lactarius
7 JQ396448 Lactarius
34 MN622728 Lactarius gracilis
1 MK253504 Lactarius subgracilis
1 AB597657 Lactarius
2 KJ458981 Lactarius perparvus
15 MH447576 Lactarius furfuraceus
4 MK167421 Lactarius hirtipes
2 MH984915 Lactarius akahatsu
28 MG589776 Lactarius alpinihirtipes
5 MK351924 Lactarius aurantiobrunneus
1 MG274144 Lactarius
1 MG274143 Lactarius
1 KF432976 Lactarius atlanticus
10 JN003629 Lactarius strigosipes
13 MH179120 Lactarius cf. rufulus
14 MH985007 Lactarius reticulisporus
18 MH984981 Lactarius sp. HL 2018u
4 KF432989 Lactarius laccarioides
2 MG770939 Lactarius subatlanticus
11 MH984960 Lactarius sp. HL 2018o
1 KF133274 Lactarius falcatus
1 KY270490 Lactarius spinosporus
2 FJ389446 Lactarius
4 HE820395 Lactarius
1 MF074771 Lactarius sp. in Russulaceae
1 KX899451 uncultured Russulaceae
5 AY456343 Lactarius
28 JF960852 Lactarius eucalypti
1 EU019923 Lactarius eucalypti
13 FJ897202 Lactarius
1 JF506799 uncultured Russula
11 KF433000 Lactarius
1 MH984999 Lactarius parallelus
1 UDB013358|110761 Lactarius
5 KF433001 Lactarius inconspicuus
1 AB973760 Lactarius sp. 2D10
1 AB218105 Lactarius
9 MH985002 Lactarius pectinatus
2 UDB013743|CP037Light Lactarius
1 LC096478 Lactarius
1 KX899896 Lactarius
1 KX899197 Lactarius
1 KX899641 uncultured Lactarius
33 KX899397 uncultured Lactarius
1 KX899048 uncultured Lactarius
1 MG018083 Lactarius
5 KF432996 Lactarius sublaccarioides
8 KP012744 Lactarius sp. 1 GMB 2014
1 KP012798 Lactarius sp. 3 GMB 2014
1 KP012799 Lactarius sp. 4 GMB 2014
2 KP012812 Lactarius sp. 2 GMB 2014
1 JF960854 Lactarius
2 KF386764 Lactarius
1 JF960853 Lactarius
1 MG018076 Lactarius sp. 2 KM 2017
3 AB828027 Lactarius
6 MH635422 Lactarius sp. NAM 2018
7 KM595003 Lactarius
34 MH590889 Lactarius kesiyae
5 KY649441 Lactarius kesiyae
1 UDB012565|HE979371|S761 Lactarius
85
2 UDB017819|UK968 Lactarius
1 MK397034 Lactarius
2 MH016796 Zelleromyces cinnabarinus
64 UDB008601|IO 553 NA
3 AM930233 Lactarius chrysorrheus
54 UDB019764|101159 Lactarius chrysorrheus
12 OWN Rodriguez P 2 Lactarius deceptivus
1 LC436081 Lactarius
12 MH984997 Lactarius
1 GU256225 Lactarius chrysorrheus
14 MK069511 Lactarius
19 EU726293 Lactarius xanthogalactus
1 MT237509 Lactarius colorascens
39 MK069509 Lactarius vinaceorufescens
3 KJ705218 Lactarius vinaceorufescens
3 KJ742399 Lactarius aff. chrysorrheus Verbeken 05 359
1 KJ173926 Lactarius chrysorrheus
44 MH984946 Lactarius sp. HL 2018l
1 AF096979 Lactarius zonarius
5 KF433018 Lactarius politus
14 KF433006 Lactarius
1 KY867682 Lactarius indoaquosus
1 MN906185 Lactarius mutabilis
13 MN906181 Lactarius mutabilis
20 HE820682 Lactarius
1 KP348149 Lactarius
1 1879|UDB038454 Lactarius zugazae
4 MK351941 Lactarius flaviaquosus
3 MK351940 Lactarius flaviaquosus
86
4 UDB023287|s1558 Lactarius
106 UDB000818|468 Lactarius quietus
1 HM015477 Lactarius
1 MF074788 Lactarius sp. in Russulaceae
9 MH984985 Lactarius sp. HL 2018v
31 MH984958 Lactarius sp. HL 2018n
1 MN258655 Lactarius asiae orientalis
9 MK575244 Lactarius quietus var. incanus
3 MK351935 Lactarius aquosus
10
5
[mya]

Figure S3 z

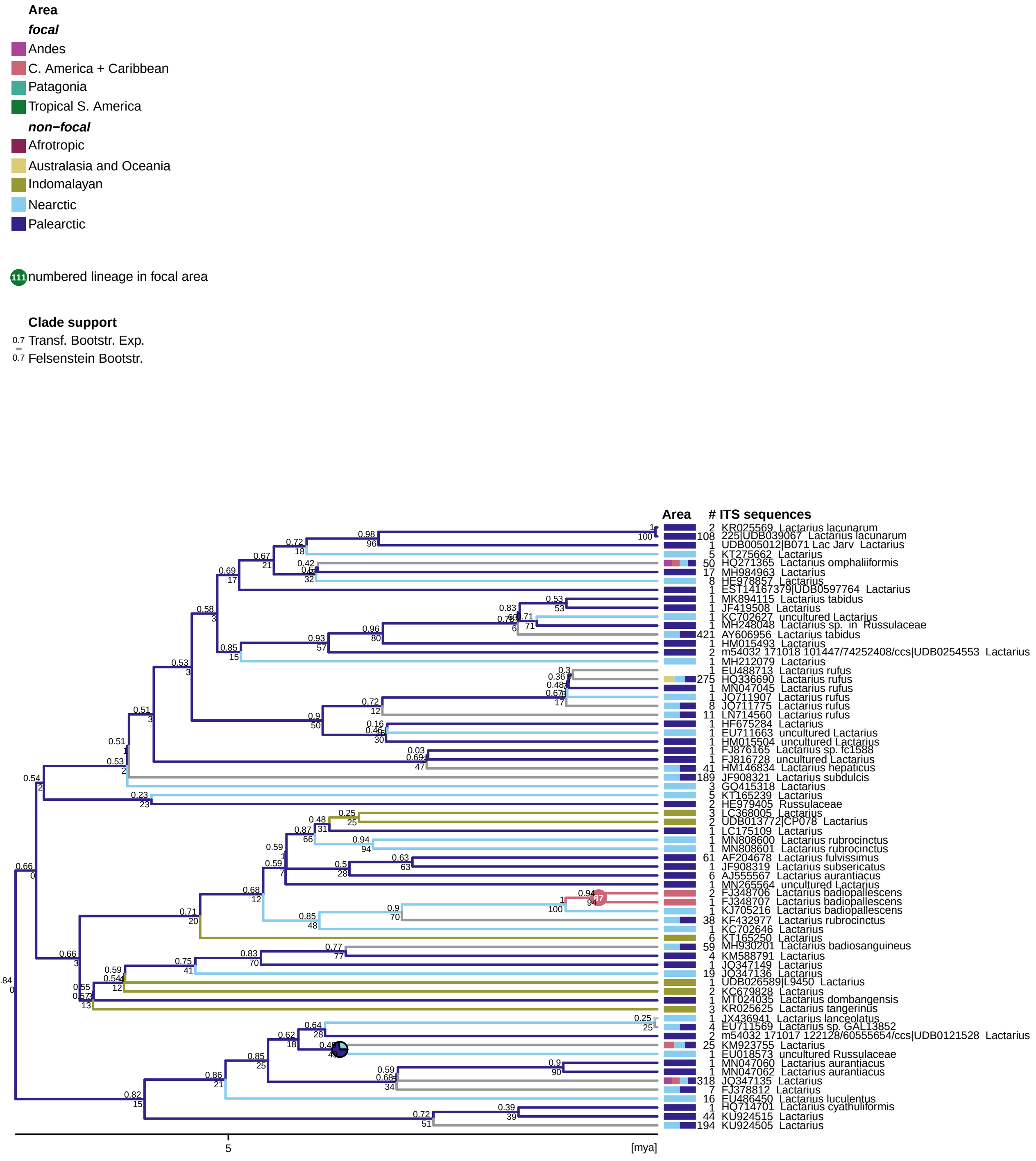
Area
focal
Andes
C. America + Caribbean
Patagonia
Tropical S. America
non−focal
Afrotropic
Australasia and Oceania
Indomalayan
Nearctic
Palearctic
numbered lineage in focal area
Clade support
0.7 Transf. Bootstr. Exp.
0.7 Felsenstein Bootstr.
Area
ITS sequences
2 KR025569 Lactarius lacunarum
108 225|UDB039067 Lactarius lacunarum
1 UDB005012|B071 Lac Jarv Lactarius
5 KT275662 Lactarius
50 HQ271365 Lactarius omphaliiformis
17 MH984963 Lactarius
8 HE978857 Lactarius
1 EST14167379|UDB0597764 Lactarius
1 MK894115 Lactarius tabidus
1 JF419508 Lactarius
1 KC702627 uncultured Lactarius
1 MH248048 Lactarius sp. in Russulaceae
421 AY606956 Lactarius tabidus
1 HM015493 Lactarius
2 m54032 171018 101447/74252408/ccs|UDB0254553 Lactarius
1 MH212079 Lactarius
1 EU488713 Lactarius rufus
275 HQ336690 Lactarius rufus
1 MN047045 Lactarius rufus
1 JQ711907 Lactarius rufus
8 JQ711775 Lactarius rufus
11 LN714560 Lactarius rufus
1 HF675284 Lactarius
1 EU711663 uncultured Lactarius
1 HM015504 uncultured Lactarius
1 FJ876165 Lactarius sp. fc1588
1 FJ816728 uncultured Lactarius
41 HM146834 Lactarius hepaticus
189 JF908321 Lactarius subdulcis
3 GQ415318 Lactarius
5 KT165239 Lactarius
2 HE979405 Russulaceae
3 LC368005 Lactarius
2 UDB013772|CP078 Lactarius
1 LC175109 Lactarius
1 MN808600 Lactarius rubrocinctus
1 MN808601 Lactarius rubrocinctus
61 AF204678 Lactarius fulvissimus
1 JF908319 Lactarius subsericatus
6 AJ555567 Lactarius aurantiacus
1 MN265564 uncultured Lactarius
2 FJ348706 Lactarius badiopallescens
1 FJ348707 Lactarius badiopallescens
1 KJ705216 Lactarius badiopallescens
38 KF432977 Lactarius rubrocinctus
1 KC702646 Lactarius
6 KT165250 Lactarius
59 MH930201 Lactarius badiosanguineus
4 KM588791 Lactarius
1 JQ347149 Lactarius
19 JQ347136 Lactarius
1 UDB026589|L9450 Lactarius
2 KC679828 Lactarius
1 MT024035 Lactarius dombangensis
3 KR025625 Lactarius tangerinus
1 JX436941 Lactarius lanceolatus
4 EU711569 Lactarius sp. GAL13852
2 m54032 171017 122128/60555654/ccs|UDB0121528 Lactarius
25 KM923755 Lactarius
1 EU018573 uncultured Russulaceae
1 MN047060 Lactarius aurantiacus
1 MN047062 Lactarius aurantiacus
318 JQ347135 Lactarius
7 FJ378812 Lactarius
16 EU486450 Lactarius luculentus
1 HQ714701 Lactarius cyathuliformis
44 KU924515 Lactarius
194 KU924505 Lactarius
5
[mya]

Figure S3 aa

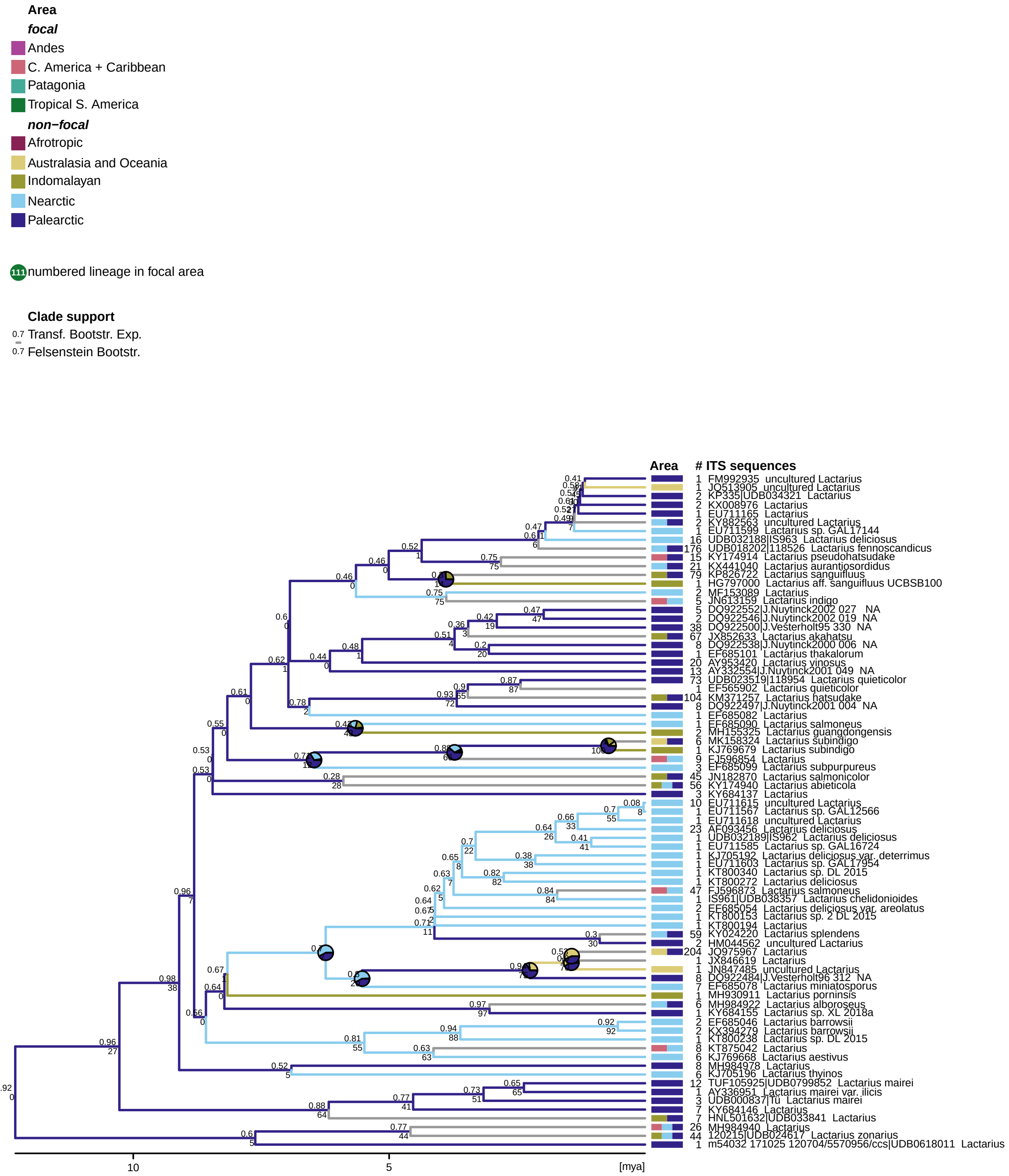
Area
focal
Andes
C. America + Caribbean
Patagonia
Tropical S. America
non−focal
Afrotropic
Australasia and Oceania
Indomalayan
Nearctic
Palearctic
111 numbered lineage in focal area
Clade support
0.7 Transf. Bootstr. Exp.
0.7 Felsenstein Bootstr.
Area
ITS sequences
1 FM992935 uncultured Lactarius
1 JQ513905 uncultured Lactarius
2 KP335|UDB034321 Lactarius
2 KX008976 Lactarius
1 EU711165 Lactarius
2 KY882563 uncultured Lactarius
1 EU711599 Lactarius sp. GAL17144
16 UDB032188|IS963 Lactarius deliciosus
176 UDB018202|118526 Lactarius fennoscandicus
15 KY174914 Lactarius pseudohatsudake
21 KX441040 Lactarius aurantiosordidus
79 KP826722 Lactarius sanguifluus
1 HG797000 Lactarius aff. sanguifluus UCBSB100
2 MF153089 Lactarius
5 JN613159 Lactarius indigo
5 DQ922552|J.Nuytinck2002 027 NA
2 DQ922546|J.Nuytinck2002 019 NA
38 DQ922500|J.Vesterholt95 330 NA
67 JX852633 Lactarius akahatsu
8 DQ922538|J.Nuytinck2000 006 NA
1 EF685101 Lactarius thakalorum
20 AY953420 Lactarius vinosus
13 AY332554|J.Nuytinck2001 049 NA
73 UDB023519|118954 Lactarius quieticolor
1 EF565902 Lactarius quieticolor
104 KM371257 Lactarius hatsudake
8 DQ922497|J.Nuytinck2001 004 NA
1 EF685082 Lactarius
1 EF685090 Lactarius salmoneus
2 MH155325 Lactarius guangdongensis
6 MK158324 Lactarius subindigo
1 KJ769679 Lactarius subindigo
9 FJ596854 Lactarius
3 EF685099 Lactarius subpurpureus
45 JN182870 Lactarius salmonicolor
56 KY174940 Lactarius abieticola
3 KY684137 Lactarius
10 EU711615 uncultured Lactarius
1 EU711567 Lactarius sp. GAL12566
1 EU711618 uncultured Lactarius
23 AF093456 Lactarius deliciosus
1 UDB032189|IS962 Lactarius deliciosus
1 EU711585 Lactarius sp. GAL16724
1 KJ705192 Lactarius deliciosus var. deterrimus
1 EU711603 Lactarius sp. GAL17954
1 KT800340 Lactarius sp. DL 2015
1 KT800272 Lactarius deliciosus
47 FJ596873 Lactarius salmoneus
1 IS961|UDB038357 Lactarius chelidonioides
2 EF685054 Lactarius deliciosus var. areolatus
1 KT800153 Lactarius sp. 2 DL 2015
1 KT800194 Lactarius
59 KY024220 Lactarius splendens
2 HM044562 uncultured Lactarius
204 JQ975967 Lactarius
1 JX846619 Lactarius
1 JN847485 uncultured Lactarius
8 DQ922484|J.Vesterholt96 312 NA
7 EF685078 Lactarius miniatosporus
1 MH930911 Lactarius porninsis
6 MH984922 Lactarius alboroseus
1 KY684155 Lactarius sp. XL 2018a
2 EF685046 Lactarius barrowsii
2 KX394279 Lactarius barrowsii
1 KT800238 Lactarius sp. DL 2015
8 KT875042 Lactarius
6 KJ769668 Lactarius aestivus
8 MH984978 Lactarius
6 KJ705196 Lactarius thyinos
12 TUF105925|UDB0799852 Lactarius mairei
1 AY336951 Lactarius mairei var. ilicis
3 UDB000837|Tü Lactarius mairei
7 KY684146 Lactarius
7 HNL501632|UDB033841 Lactarius
26 MH984940 Lactarius
44 120215|UDB024617 Lactarius zonarius
1 m54032 171025 120704/5570956/ccs|UDB0618011 Lactarius
10
5
[mya]

Figure S3 ab

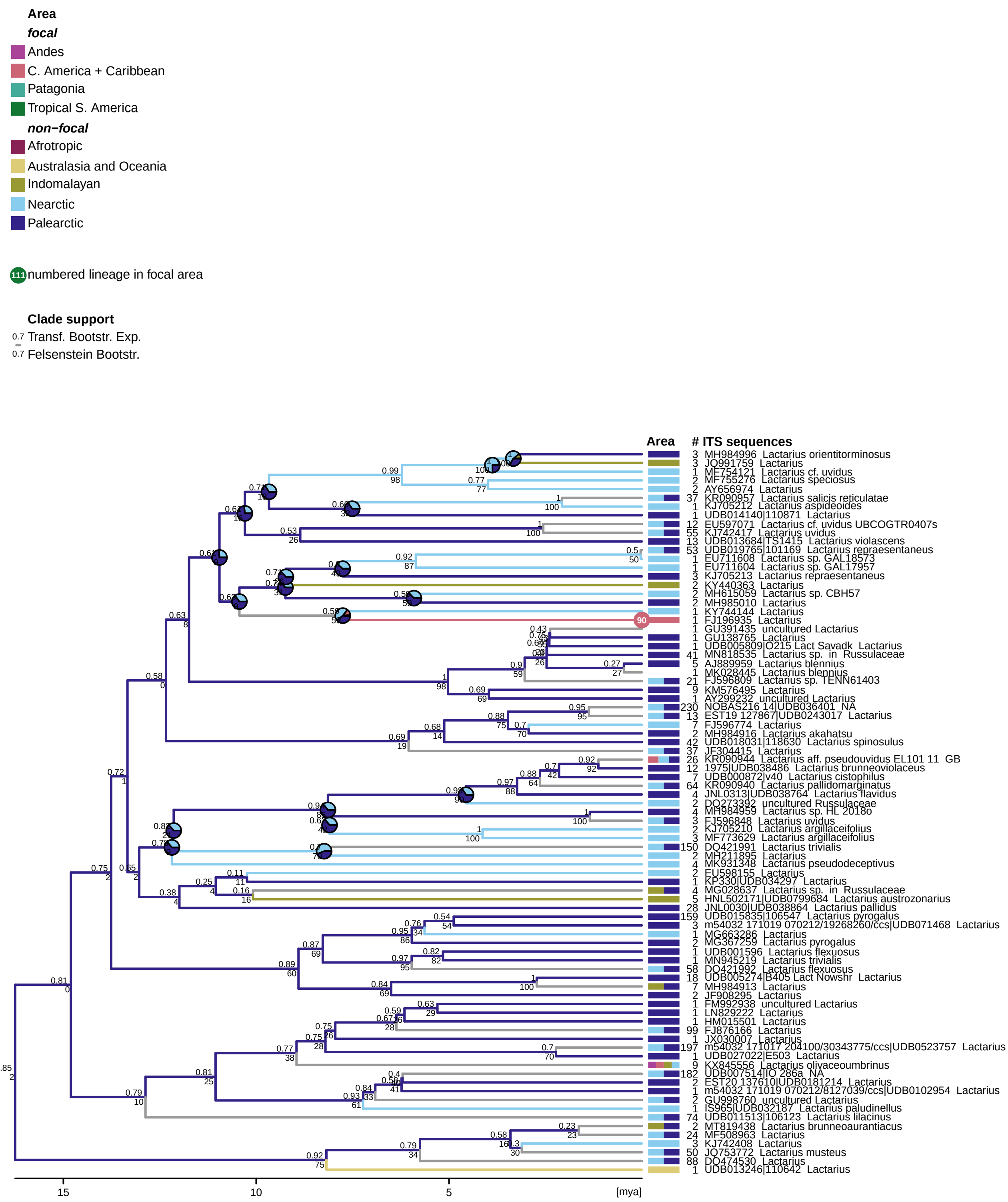
Area
focal
Andes
C. America + Caribbean
Patagonia
Tropical S. America
non−focal
Afrotropic
Australasia and Oceania
Indomalayan
Nearctic
Palearctic
111 numbered lineage in focal area
Clade support
0.7 Transf. Bootstr. Exp.
0.7 Felsenstein Bootstr.
Area
ITS sequences
3 MH984996 Lactarius orientitorminosus
3 JQ991759 Lactarius
1 MF754121 Lactarius cf. uvidus
2 MF755276 Lactarius speciosus
2 AY656974 Lactarius
37 KR090957 Lactarius salicis reticulatae
1 KJ705212 Lactarius aspideoides
1 UDB014140|110871 Lactarius
12 EU597071 Lactarius cf. uvidus UBCOGTR0407s
55 KJ742417 Lactarius uvidus
13 UDB013684|TS1415 Lactarius violascens
53 UDB019765|101169 Lactarius repraesentaneus
1 EU711608 Lactarius sp. GAL18573
1 EU711604 Lactarius sp. GAL17957
3 KJ705213 Lactarius repraesentaneus
2 KY440363 Lactarius
2 MH615059 Lactarius sp. CBH57
2 MH985010 Lactarius
1 KY744144 Lactarius
90
1 FJ196935 Lactarius
1 GU391435 uncultured Lactarius
1 GU138765 Lactarius
1 UDB005809|O215 Lact Savadk Lactarius
41 MN818535 Lactarius sp. in Russulaceae
5 AJ889959 Lactarius blennius
1 MK028445 Lactarius blennius
21 FJ596809 Lactarius sp. TENN61403
9 KM576495 Lactarius
1 AY299232 uncultured Lactarius
230 NOBAS216 14|UDB036401 NA
13 EST19 127867|UDB0243017 Lactarius
7 FJ596774 Lactarius
2 MH984916 Lactarius akahatsu
42 UDB018031|118630 Lactarius spinosulus
37 JF304415 Lactarius
26 KR090944 Lactarius aff. pseudouvidus EL101 11 GB
12 1975|UDB038486 Lactarius brunneoviolaceus
7 UDB000872|v40 Lactarius cistophilus
64 KR090940 Lactarius pallidomarginatus
4 JNL0313|UDB038764 Lactarius flavidus
2 DQ273392 uncultured Russulaceae
4 MH984959 Lactarius sp. HL 2018o
3 FJ596848 Lactarius uvidus
2 KJ705210 Lactarius argillaceifolius
3 MF773629 Lactarius argillaceifolius
150 DQ421991 Lactarius trivialis
2 MH211895 Lactarius
4 MK931348 Lactarius pseudodeceptivus
2 EU598155 Lactarius
1 KP330|UDB034297 Lactarius
4 MG028637 Lactarius sp. in Russulaceae
5 HNL502171|UDB0799684 Lactarius austrozonarius
28 JNL0030|UDB038864 Lactarius pallidus
159 UDB015835|106547 Lactarius pyrogalus
3 m54032 171019 070212/19268260/ccs|UDB071468 Lactarius
1 MG663286 Lactarius
2 MG367259 Lactarius pyrogalus
1 UDB001596 Lactarius flexuosus
1 MN945219 Lactarius trivialis
58 DQ421992 Lactarius flexuosus
18 UDB005274|B405 Lact Nowshr Lactarius
7 MH984913 Lactarius
2 JF908295 Lactarius
1 FM992938 uncultured Lactarius
1 LN829222 Lactarius
1 HM015501 Lactarius
99 FJ876166 Lactarius
1 JX030007 Lactarius
197 m54032 171017 204100/30343775/ccs|UDB0523757 Lactarius
1 UDB027022|E503 Lactarius
9 KX845556 Lactarius olivaceoumbrinus
182 UDB007514|IO 286a NA
2 EST20 137610|UDB0181214 Lactarius
1 m54032 171019 070212/8127039/ccs|UDB0102954 Lactarius
2 GU998760 uncultured Lactarius
1 IS965|UDB032187 Lactarius paludinellus
74 UDB011513|106123 Lactarius lilacinus
2 MT819438 Lactarius brunneoaurantiacus
24 MF508963 Lactarius
3 KJ742408 Lactarius
50 JQ753772 Lactarius musteus
88 DQ474530 Lactarius
1 UDB013246|110642 Lactarius
15
10
5
[mya]

Figure S3 ac

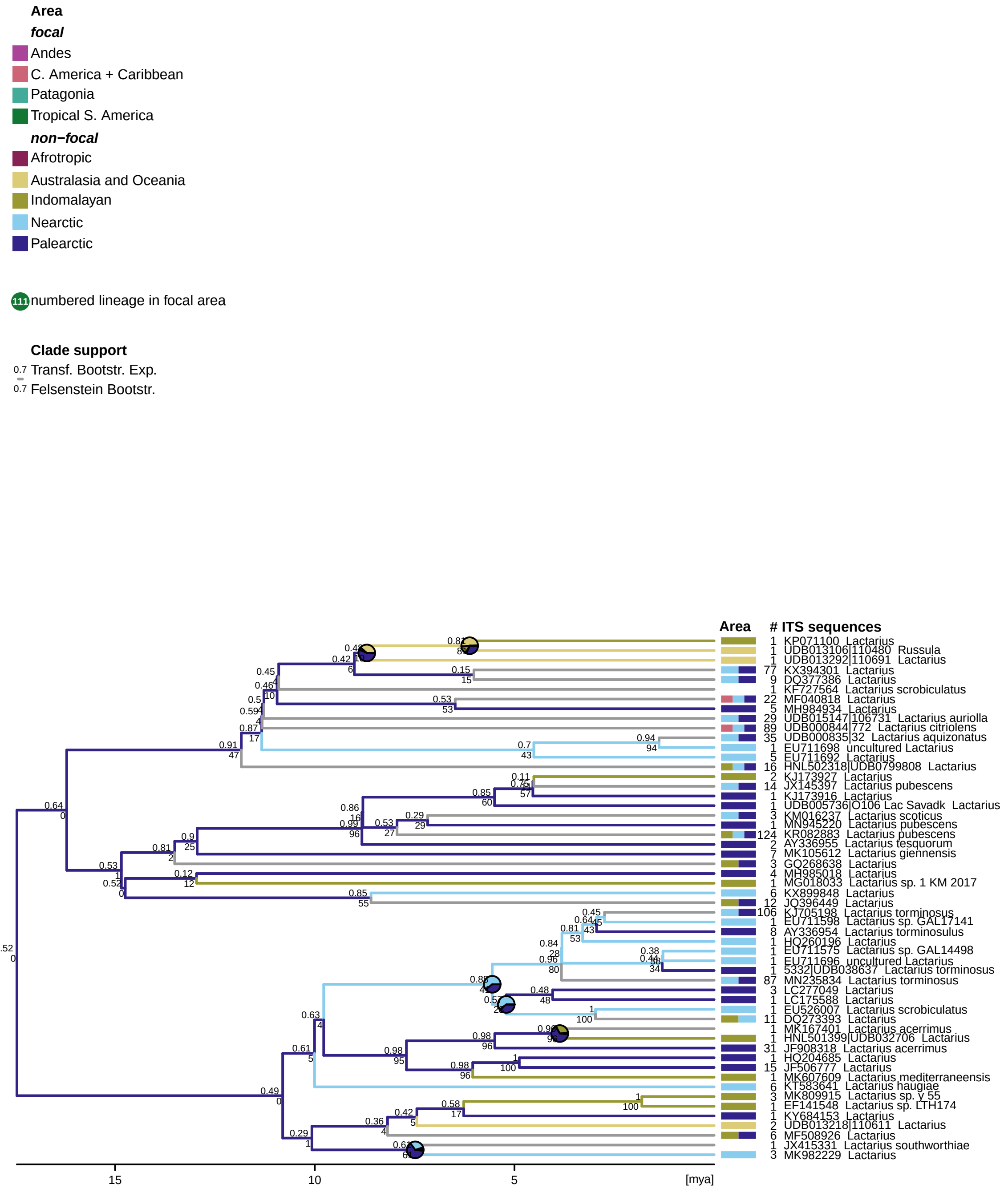

Area
focal
Andes
C. America + Caribbean
Patagonia
Tropical S. America
non−focal
Afrotropic
Australasia and Oceania
Indomalayan
Nearctic
Palearctic
111 numbered lineage in focal area
Clade support
0.7 Transf. Bootstr. Exp.
0.7 Felsenstein Bootstr.
Area
ITS sequences
1 KP071100 Lactarius
1 UDB013106|110480 Russula
1 UDB013292|110691 Lactarius
77 KX394301 Lactarius
9 DQ377386 Lactarius
1 KF727564 Lactarius scrobiculatus
22 MF040818 Lactarius
5 MH984934 Lactarius
29 UDB015147|106731 Lactarius auriolla
89 UDB000844|772 Lactarius citriolens
35 UDB000835|32 Lactarius aquizonatus
1 EU711698 uncultured Lactarius
5 EU711692 Lactarius
16 HNL502318|UDB0799808 Lactarius
2 KJ173927 Lactarius
14 JX145397 Lactarius pubescens
1 KJ173916 Lactarius
1 UDB005736|O106 Lac Savadk Lactarius
3 KM016237 Lactarius scoticus
1 MN945220 Lactarius pubescens
124 KR082883 Lactarius pubescens
2 AY336955 Lactarius tesquorum
7 MK105612 Lactarius giennensis
3 GQ268638 Lactarius
4 MH985018 Lactarius
1 MG018033 Lactarius sp. 1 KM 2017
6 KX899848 Lactarius
12 JQ396449 Lactarius
106 KJ705198 Lactarius torminosus
1 EU711598 Lactarius sp. GAL17141
8 AY336954 Lactarius torminosulus
1 HQ260196 Lactarius
1 EU711575 Lactarius sp. GAL14498
1 EU711696 uncultured Lactarius
1 5332|UDB038637 Lactarius torminosus
87 MN235834 Lactarius torminosus
3 LC277049 Lactarius
1 LC175588 Lactarius
1 EU526007 Lactarius scrobiculatus
11 DQ273393 Lactarius
1 MK167401 Lactarius acerrimus
1 HNL501399|UDB032706 Lactarius
31 JF908318 Lactarius acerrimus
1 HQ204685 Lactarius
15 JF506777 Lactarius
1 MK607609 Lactarius mediterraneensis
6 KT583641 Lactarius haugiae
3 MK809915 Lactarius sp. y 55
1 EF141548 Lactarius sp. LTH174
1 KY684153 Lactarius
2 UDB013218|110611 Lactarius
6 MF508926 Lactarius
1 JX415331 Lactarius southworthiae
3 MK982229 Lactarius
15
10
5
[mya]

Figure S3 ad

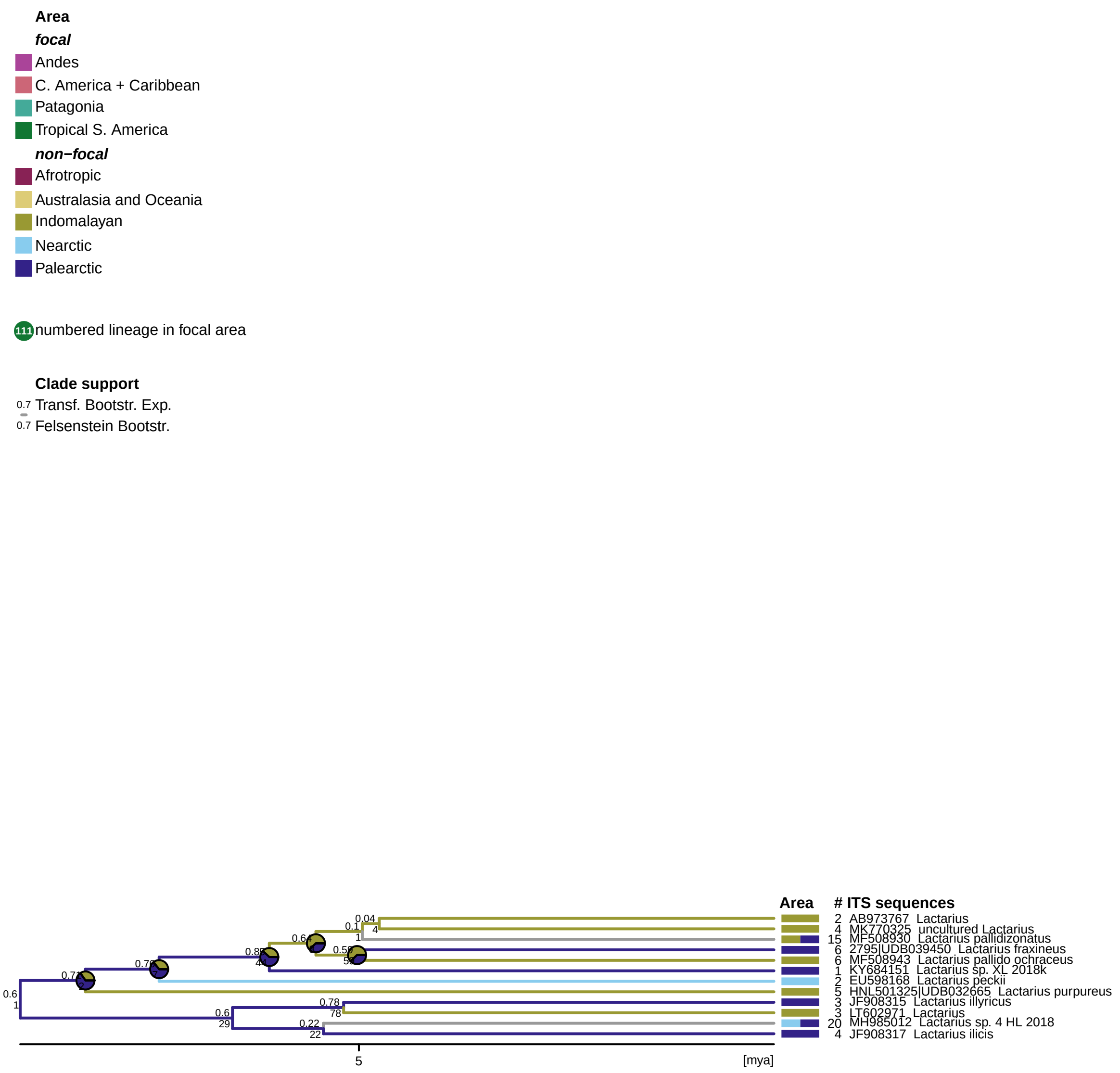

Area
focal
Andes
C. America + Caribbean
Patagonia
Tropical S. America
non−focal
Afrotropic
Australasia and Oceania
Indomalayan
Nearctic
Palearctic
111 numbered lineage in focal area
Clade support
0.7 Transf. Bootstr. Exp.
0.7 Felsenstein Bootstr.
Area
ITS sequences
2 AB973767 Lactarius
4 MK770325 uncultured Lactarius
15 MF508930 Lactarius pallidizonatus
6 2795|UDB039450 Lactarius fraxineus
6 MF508943 Lactarius pallido ochraceus
1 KY684151 Lactarius sp. XL 2018k
2 EU598168 Lactarius peckii
5 HNL501325|UDB032665 Lactarius purpureus
3 JF908315 Lactarius illyricus
3 LT602971 Lactarius
20 MH985012 Lactarius sp. 4 HL 2018
4 JF908317 Lactarius ilicis
5
[mya]

Figure S3 ae

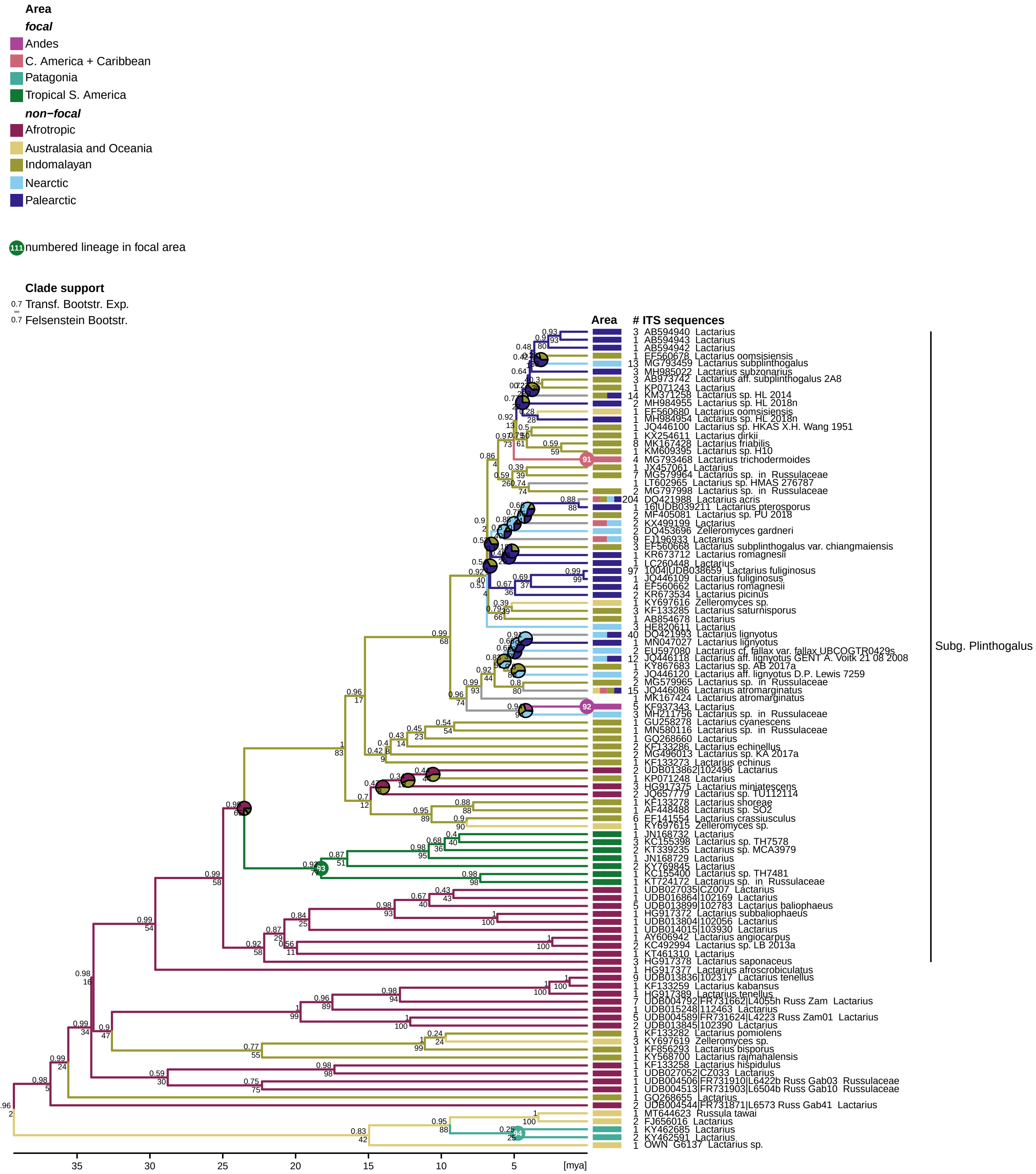

Area
focal
Andes
C. America + Caribbean
Patagonia
Tropical S. America
non−focal
Afrotropic
Australasia and Oceania
Indomalayan
Nearctic
Palearctic
111
numbered lineage in focal area
Clade support
0.7 Transf. Bootstr. Exp.
0.7 Felsenstein Bootstr.
Area
ITS sequences
3 AB594940 Lactarius
1 AB594943 Lactarius
1 AB594942 Lactarius
1 EF560678 Lactarius oomsisiensis
13 MG793459 Lactarius subplinthogalus
3 MH985022 Lactarius subzonarius
3 AB973742 Lactarius aff. subplinthogalus 2A8
1 KP071243 Lactarius
14 KM371258 Lactarius sp. HL 2014
2 MH984955 Lactarius sp. HL 2018n
1 EF560680 Lactarius oomsisiensis
1 MH984954 Lactarius sp. HL 2018n
1 JQ446100 Lactarius sp. HKAS X.H. Wang 1951
1 KX254611 Lactarius dirkii
8 MK167428 Lactarius friabilis
1 KM609395 Lactarius sp. H10
91
4 MG793468 Lactarius trichodermoides
1 JX457061 Lactarius
7 MG579964 Lactarius sp. in Russulaceae
1 LT602965 Lactarius sp. HMAS 276787
2 MG797998 Lactarius sp. in Russulaceae
204 DQ421988 Lactarius acris
1 16|UDB039211 Lactarius pterosporus
2 MF405081 Lactarius sp. PU 2018
2 KX499199 Lactarius
2 DQ453696 Zelleromyces gardneri
9 FJ196933 Lactarius
3 EF560668 Lactarius subplinthogalus var. chiangmaiensis
1 KR673712 Lactarius romagnesii
1 LC260448 Lactarius
97 1004|UDB038659 Lactarius fuliginosus
1 JQ446109 Lactarius fuliginosus
4 EF560662 Lactarius romagnesii
2 KR673534 Lactarius picinus
1 KY697616 Zelleromyces sp.
3 KF133285 Lactarius saturnisporus
1 AB854678 Lactarius
3 HE820611 Lactarius
40 DQ421993 Lactarius lignyotus
1 MN047027 Lactarius lignyotus
2 EU597080 Lactarius cf. fallax var. fallax UBCOGTR0429s
12 JQ446118 Lactarius aff. lignyotus GENT A. Voitk 21 08 2008
1 KY867683 Lactarius sp. AB 2017a
2 JQ446120 Lactarius aff. lignyotus D.P. Lewis 7259
2 MG579965 Lactarius sp. in Russulaceae
15 JQ446086 Lactarius atromarginatus
1 MK167424 Lactarius atromarginatus
92
5 KF937343 Lactarius
3 MH211756 Lactarius sp. in Russulaceae
1 GU258278 Lactarius cyanescens
1 MN580116 Lactarius sp. in Russulaceae
1 GQ268660 Lactarius
2 KF133286 Lactarius echinellus
2 MG496013 Lactarius sp. KA 2017a
1 KF133273 Lactarius echinus
2 UDB013862|102496 Lactarius
1 KP071248 Lactarius
3 HG917375 Lactarius miniatescens
2 JQ657779 Lactarius sp. TU112114
1 KF133278 Lactarius shoreae
1 AF448488 Lactarius sp. SO2
6 EF141554 Lactarius crassiusculus
1 KY697615 Zelleromyces sp.
93
1 JN168732 Lactarius
3 KC155398 Lactarius sp. TH7578
2 KT339235 Lactarius sp. MCA3979
1 JN168729 Lactarius
2 KY769845 Lactarius
1 KC155400 Lactarius sp. TH7481
1 KT724172 Lactarius sp. in Russulaceae
1 UDB027035|CZ007 Lactarius
1 UDB016864|102169 Lactarius
5 UDB013899|102783 Lactarius baliophaeus
1 HG917372 Lactarius subbaliophaeus
1 UDB013804|102056 Lactarius
1 UDB014015|103930 Lactarius
1 AY606942 Lactarius angiocarpus
2 KC492994 Lactarius sp. LB 2013a
1 KT461310 Lactarius
3 HG917378 Lactarius saponaceus
1 HG917377 Lactarius afroscrobiculatus
9 UDB013836|102317 Lactarius tenellus
1 KF133259 Lactarius kabansus
1 HG917389 Lactarius tenellus
7 UDB004792|FR731662|L4055h Russ Zam Lactarius
1 UDB015248|112463 Lactarius
5 UDB004589|FR731624|L4223 Russ Zam01 Lactarius
2 UDB013845|102390 Lactarius
1 KF133282 Lactarius pomiolens
3 KY697619 Zelleromyces sp.
1 KF856293 Lactarius bisporus
1 KY568700 Lactarius rajmahalensis
1 KF133258 Lactarius hispidulus
1 UDB027052|CZ033 Lactarius
1 UDB004506|FR731910|L6422b Russ Gab03 Russulaceae
1 UDB004513|FR731903|L6504b Russ Gab10 Russulaceae
1 GQ268655 Lactarius
2 UDB004544|FR731871|L6573 Russ Gab41 Lactarius
1 MT644623 Russula tawai
2 FJ656016 Lactarius
94
1 KY462685 Lactarius
2 KY462591 Lactarius
1 OWN G6137 Lactarius sp.
Subg. Plinthogalus
35 30 25 20 15 10 5 [mya]

Figure S3 af

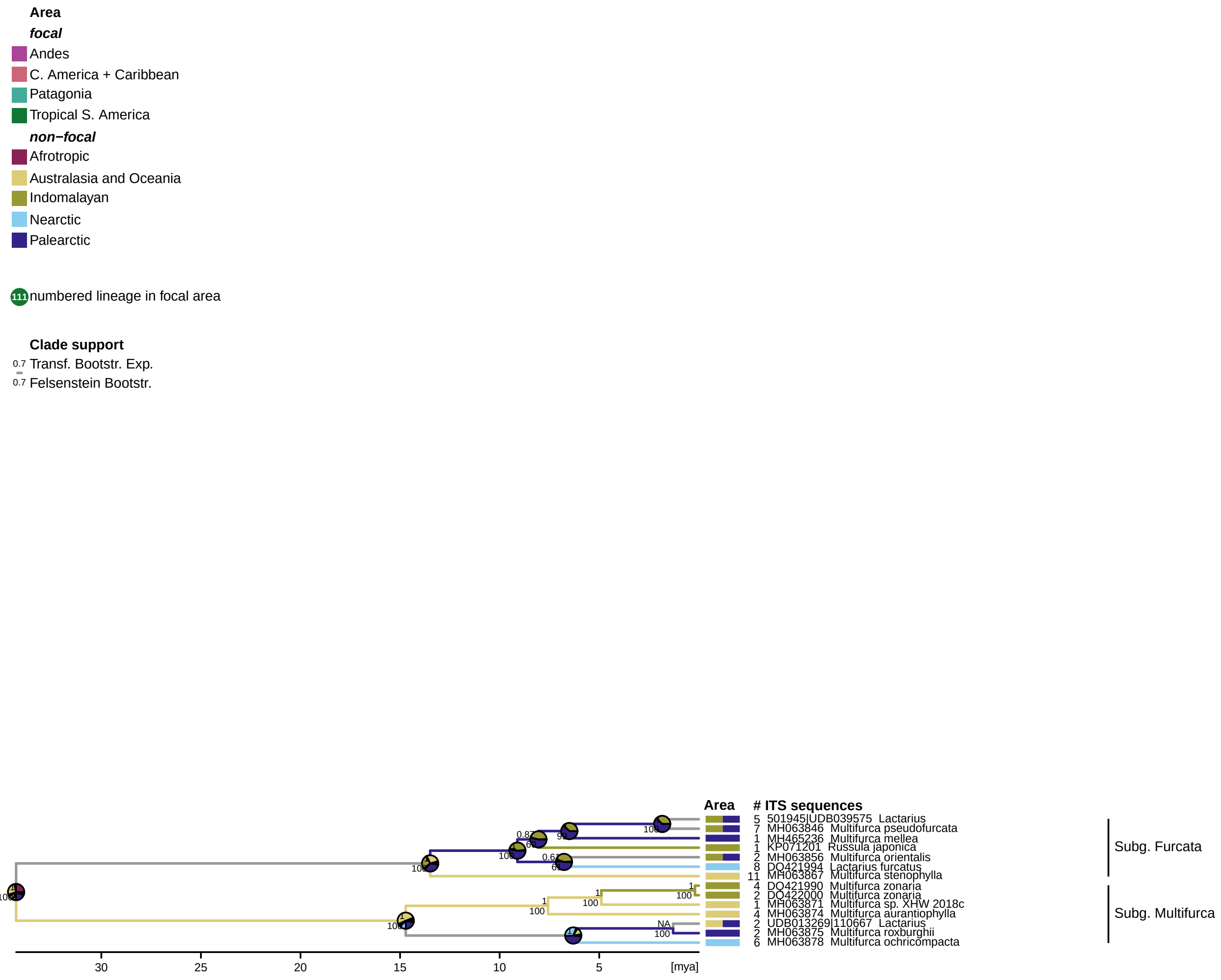

Area
focal
Andes
C. America + Caribbean
Patagonia
Tropical S. America
non−focal
Afrotropic
Australasia and Oceania
Indomalayan
Nearctic
Palearctic
111
numbered lineage in focal area
Clade support
0.7 Transf. Bootstr. Exp.
0.7 Felsenstein Bootstr.
Area
ITS sequences
5 501945|UDB039575 Lactarius
7 MH063846 Multifurca pseudofurcata
1 MH465236 Multifurca mellea
1 KP071201 Russula japonica
2 MH063856 Multifurca orientalis
8 DQ421994 Lactarius furcatus
11 MH063867 Multifurca stenophylla
4 DQ421990 Multifurca zonaria
2 DQ422000 Multifurca zonaria
1 MH063871 Multifurca sp. XHW 2018c
4 MH063874 Multifurca aurantiophylla
2 UDB013269|110667 Lactarius
2 MH063875 Multifurca roxburghii
6 MH063878 Multifurca ochricompacta
Subg. Furcata
Subg. Multifurca
30
25
20
15
10
5
[mya]

Figure S3 ag

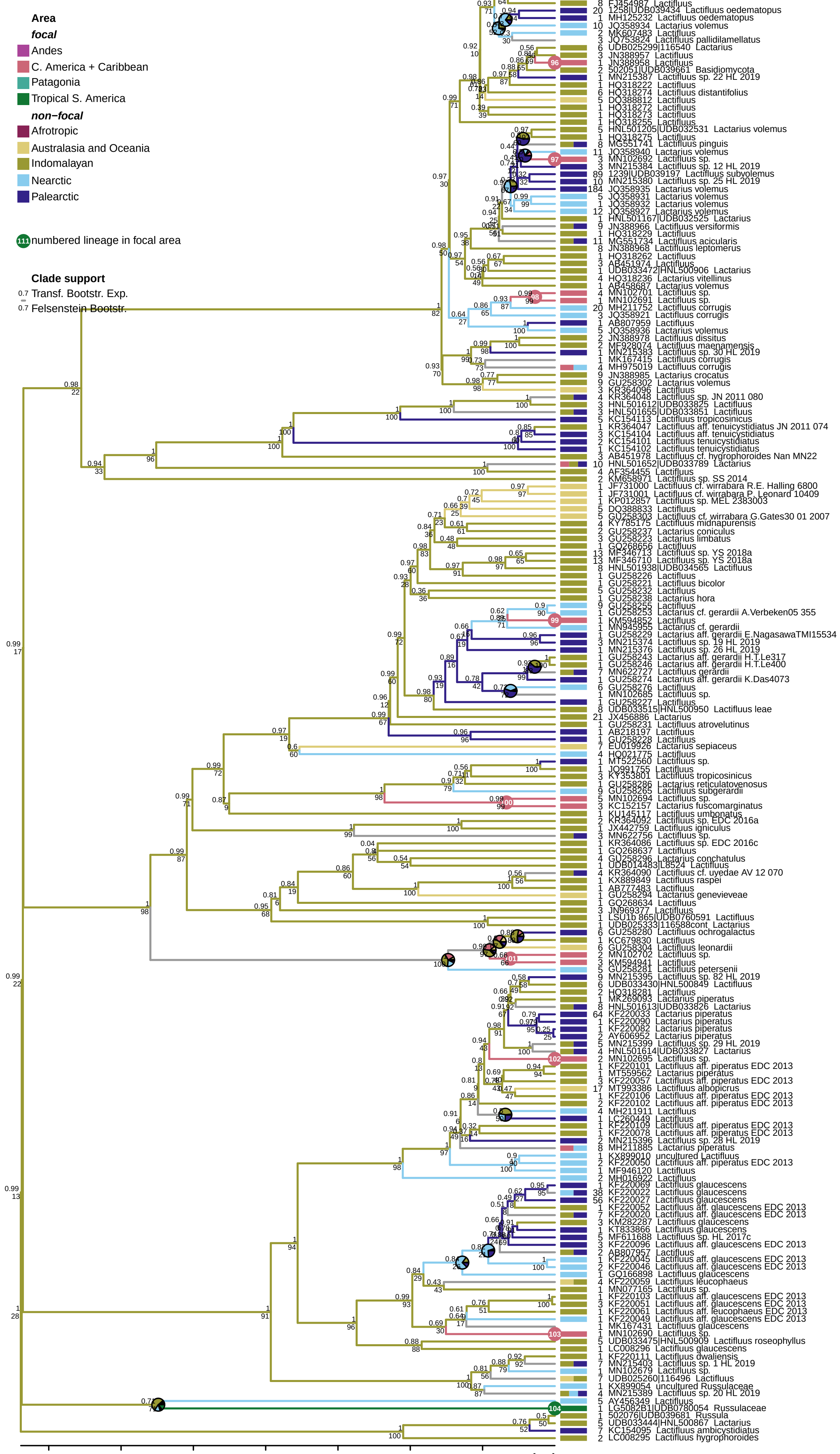

Area
focal
Andes
C. America + Caribbean
Patagonia
Tropical S. America
non−focal
Afrotropic
Australasia and Oceania
Indomalayan
Nearctic
Palearctic
111 numbered lineage in focal area
Clade support
0.7 Transf. Bootstr. Exp.
0.7 Felsenstein Bootstr.
Area
ITS sequences
1 HQ318232 Lactarius volemus
5 JN388986 Lactarius cf. volemus KP 2012
4 KU552085 Lactifluus
7 MN215377 Lactifluus sp. 9 HL 2019
1 MN005115 Lactifluus sp. IB 2019a
4 HQ318261 Lactarius volemus
8 FJ454987 Lactifluus
20 1258|UDB039434 Lactifluus oedematopus
1 MH125232 Lactifluus oedematopus
10 JQ358934 Lactarius volemus
2 MK607483 Lactifluus
3 JQ753824 Lactifluus pallidilamellatus
6 UDB025299|116540 Lactarius
3 JN388957 Lactifluus
96
1 JN388958 Lactifluus
2 502051|UDB039661 Basidiomycota
1 MN215387 Lactifluus sp. 22 HL 2019
1 HQ318222 Lactifluus
6 HQ318274 Lactifluus distantifolius
5 DQ388812 Lactifluus
1 HQ318272 Lactifluus
1 HQ318273 Lactifluus
1 HQ318255 Lactifluus
5 HNL501205|UDB032531 Lactarius volemus
1 HQ318275 Lactifluus
8 MG551741 Lactifluus pinguis
11 JQ358940 Lactarius volemus
97
3 MN102692 Lactifluus sp.
3 MN215384 Lactifluus sp. 12 HL 2019
89 1239|UDB039197 Lactifluus subvolemus
10 MN215380 Lactifluus sp. 25 HL 2019
184 JQ358935 Lactarius volemus
5 JQ358931 Lactarius volemus
1 JQ358932 Lactarius volemus
12 JQ358927 Lactarius volemus
1 HNL501167|UDB032525 Lactarius
9 JN388966 Lactifluus versiformis
1 HQ318229 Lactifluus
11 MG551734 Lactifluus acicularis
8 JN388968 Lactifluus leptomerus
1 HQ318262 Lactifluus
3 AB451974 Lactifluus
1 UDB033472|HNL500906 Lactarius
4 HQ318236 Lactarius vitellinus
1 AB458687 Lactarius volemus
98
4 MN102701 Lactifluus sp.
1 MN102691 Lactifluus sp.
20 MH211752 Lactifluus corrugis
3 JQ358921 Lactifluus corrugis
1 AB807959 Lactifluus
5 JQ358936 Lactarius volemus
2 JN388978 Lactifluus dissitus
2 MF928074 Lactifluus maenamensis
1 MN215383 Lactifluus sp. 30 HL 2019
1 MK167415 Lactifluus corrugis
4 MH975019 Lactifluus corrugis
9 JN388985 Lactarius crocatus
9 GU258302 Lactarius volemus
3 KR364096 Lactifluus
4 KR364048 Lactifluus sp. JN 2011 080
3 HNL501612|UDB033825 Lactifluus
3 HNL501655|UDB033851 Lactifluus
5 KC154113 Lactifluus tropicosinicus
1 KR364047 Lactifluus aff. tenuicystidiatus JN 2011 074
3 KC154104 Lactifluus aff. tenuicystidiatus
2 KC154101 Lactifluus tenuicystidiatus
1 KC154102 Lactifluus tenuicystidiatus
3 AB451978 Lactifluus cf. hygrophoroides Nan MN22
10 HNL501652|UDB033789 Lactarius
4 AF354455 Lactifluus
2 KM658971 Lactifluus sp. SS 2014
1 JF731000 Lactifluus cf. wirrabara R.E. Halling 6800
1 JF731001 Lactifluus cf. wirrabara P. Leonard 10409
1 KP012857 Lactifluus sp. MEL 2383003
5 DQ388833 Lactifluus
5 GU258303 Lactifluus cf. wirrabara G.Gates30 01 2007
4 KY785175 Lactifluus midnapurensis
2 GU258237 Lactarius coniculus
3 GU258223 Lactarius limbatus
1 GQ268656 Lactifluus
13 MF346713 Lactifluus sp. YS 2018a
13 MF346710 Lactifluus sp. YS 2018a
8 HNL501938|UDB034565 Lactifluus
1 GU258226 Lactifluus
1 GU258221 Lactifluus bicolor
5 GU258232 Lactifluus
1 GU258238 Lactarius hora
9 GU258255 Lactifluus
1 GU258253 Lactarius cf. gerardii A.Verbeken05 355
99
1 KM594852 Lactifluus
1 MN945955 Lactarius cf. gerardii
1 GU258229 Lactarius aff. gerardii E.NagasawaTMI15534
3 MN215374 Lactifluus sp. 19 HL 2019
1 MN215376 Lactifluus sp. 26 HL 2019
1 GU258243 Lactarius aff. gerardii H.T.Le317
1 GU258246 Lactarius aff. gerardii H.T.Le400
7 MN622727 Lactifluus gerardii
1 GU258274 Lactarius aff. gerardii K.Das4073
6 GU258276 Lactifluus
1 MN102685 Lactifluus sp.
1 GU258227 Lactifluus
8 UDB033515|HNL500950 Lactifluus leae
21 JX456886 Lactarius
1 GU258231 Lactifluus atrovelutinus
1 AB218197 Lactifluus
1 GU258228 Lactifluus
7 EU019926 Lactarius sepiaceus
4 HQ021775 Lactifluus
1 MT522560 Lactifluus sp.
1 JQ991755 Lactifluus
3 KY353801 Lactifluus tropicosinicus
1 GU258286 Lactarius reticulatovenosus
9 GU258265 Lactifluus subgerardii
100
5 MN102694 Lactifluus sp.
3 KC152157 Lactarius fuscomarginatus
1 KU145117 Lactifluus umbonatus
2 KR364092 Lactifluus sp. EDC 2016a
1 JX442759 Lactifluus igniculus
3 MN622756 Lactifluus sp.
1 KR364086 Lactifluus sp. EDC 2016c
1 GQ268637 Lactifluus
4 GU258296 Lactarius conchatulus
1 UDB014483|L8524 Lactifluus
4 KR364090 Lactifluus cf. uyedae AV 12 070
1 KX889849 Lactifluus raspei
1 AB777483 Lactifluus
1 GU258294 Lactarius genevieveae
1 GQ268634 Lactifluus
3 JN969377 Lactifluus
1 LSU1b 865|UDB0760591 Lactifluus
1 UDB025333|116588cont Lactarius
6 GU258280 Lactifluus ochrogalactus
1 KC679830 Lactifluus
6 GU258304 Lactifluus leonardii
101
2 MN102702 Lactifluus sp.
3 KM594941 Lactifluus
5 GU258281 Lactifluus petersenii
9 MN215395 Lactifluus sp. 82 HL 2019
6 UDB033430|HNL500849 Lactifluus
2 HQ318281 Lactifluus
1 MK269093 Lactarius piperatus
8 HNL501613|UDB033826 Lactarius
64 KF220033 Lactarius piperatus
1 KF220090 Lactarius piperatus
1 KF220082 Lactarius piperatus
2 AY606952 Lactarius piperatus
5 MN215399 Lactifluus sp. 29 HL 2019
4 HNL501614|UDB033827 Lactarius
102
2 MN102695 Lactifluus sp.
1 KF220101 Lactifluus aff. piperatus EDC 2013
1 MT559562 Lactarius piperatus
3 KF220057 Lactifluus aff. piperatus EDC 2013
17 MT993386 Lactifluus albopicrus
1 KF220106 Lactifluus aff. piperatus EDC 2013
2 KF220102 Lactifluus aff. piperatus EDC 2013
4 MH211911 Lactifluus
1 LC260449 Lactifluus
1 KF220109 Lactifluus aff. piperatus EDC 2013
1 KF220078 Lactifluus aff. piperatus EDC 2013
2 MN215396 Lactifluus sp. 28 HL 2019
8 MH211885 Lactarius piperatus
1 KX899010 uncultured Lactifluus
2 KF220050 Lactifluus aff. piperatus EDC 2013
1 MF946120 Lactifluus
2 MH016922 Lactifluus
1 KF220069 Lactifluus glaucescens
38 KF220022 Lactifluus glaucescens
56 KF220027 Lactifluus glaucescens
1 KF220052 Lactifluus aff. glaucescens EDC 2013
7 KF220020 Lactifluus aff. glaucescens EDC 2013
3 KM282287 Lactifluus glaucescens
1 KT833866 Lactifluus glaucescens
5 MF611688 Lactifluus sp. HL 2017c
3 KF220096 Lactifluus aff. glaucescens EDC 2013
2 AB807957 Lactifluus
1 KF220045 Lactifluus aff. glaucescens EDC 2013
2 KF220046 Lactifluus aff. glaucescens EDC 2013
1 GQ166898 Lactifluus glaucescens
4 KF220059 Lactifluus leucophaeus
1 MN077165 Lactifluus sp.
1 KF220103 Lactifluus aff. glaucescens EDC 2013
3 KF220051 Lactifluus aff. glaucescens EDC 2013
1 KF220061 Lactifluus aff. leucophaeus EDC 2013
1 KF220049 Lactifluus aff. glaucescens EDC 2013
1 MK167431 Lactifluus glaucescens
103
1 MN102690 Lactifluus sp.
5 UDB033475|HNL500909 Lactifluus roseophyllus
1 LC008296 Lactifluus glaucescens
1 KF220111 Lactifluus dwaliensis
7 MN215403 Lactifluus sp. 1 HL 2019
1 MN102679 Lactifluus sp.
7 UDB025260|116496 Lactifluus
1 KX899054 uncultured Russulaceae
4 MN215389 Lactifluus sp. 20 HL 2019
5 AY456349 Lactifluus
104
1 LG5082B1|UDB0780054 Russulaceae
1 502076|UDB039681 Russula
5 UDB033444|HNL500867 Lactarius
7 KC154095 Lactifluus ambicystidiatus
2 LC008295 Lactifluus hygrophoroides
35
30
25
20
15
10
5
[mya]

Figure S3 ah

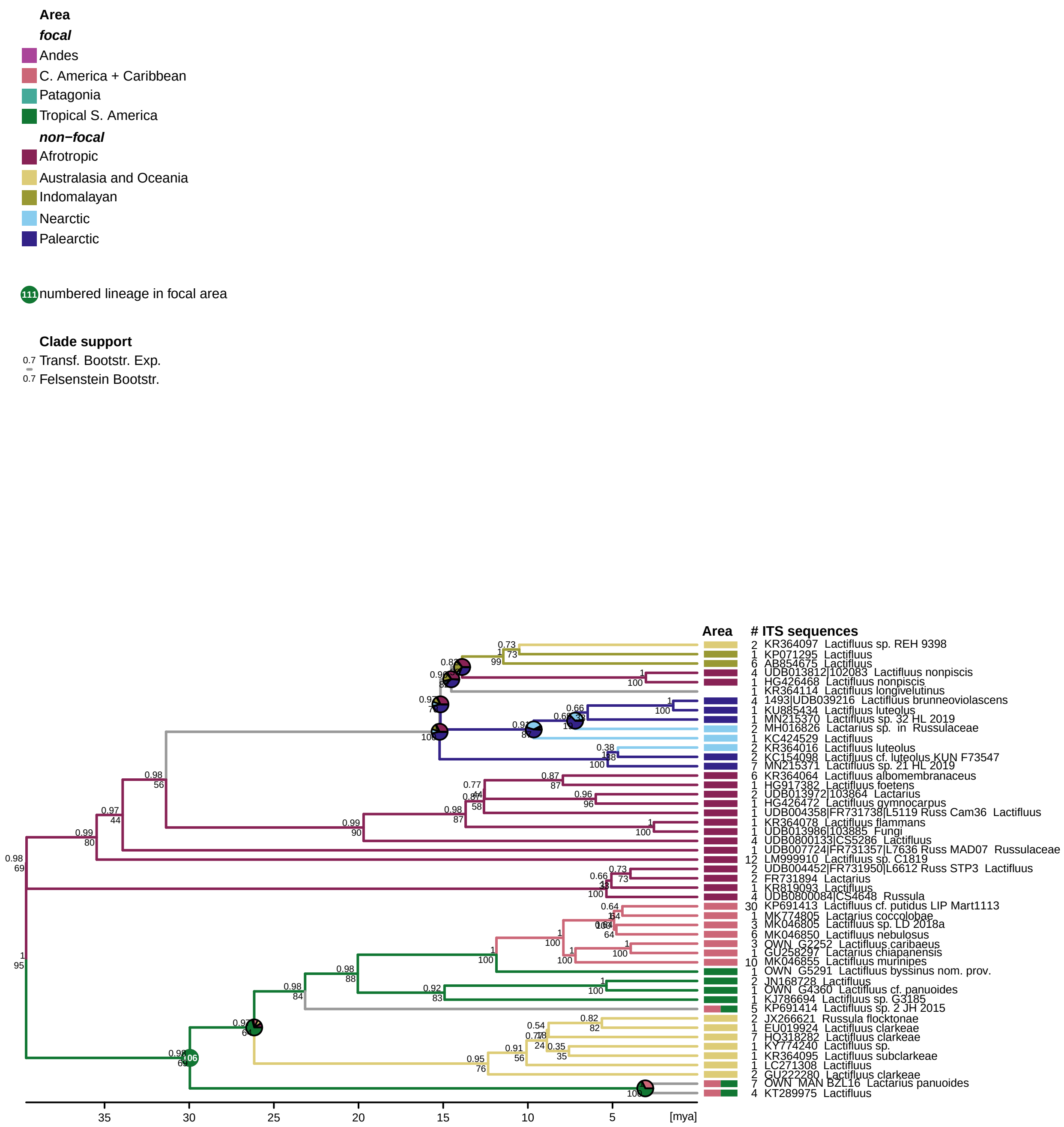
Area
focal
Andes
C. America + Caribbean
Patagonia
Tropical S. America
non−focal
Afrotropic
Australasia and Oceania
Indomalayan
Nearctic
Palearctic
111 numbered lineage in focal area
Clade support
0.7 Transf. Bootstr. Exp.
0.7 Felsenstein Bootstr.
Area
ITS sequences
2 KR364097 Lactifluus sp. REH 9398
1 KP071295 Lactifluus
6 AB854675 Lactifluus
4 UDB013812|102083 Lactifluus nonpiscis
1 HG426468 Lactifluus nonpiscis
1 KR364114 Lactifluus longivelutinus
4 1493|UDB039216 Lactifluus brunneoviolascens
1 KU885434 Lactifluus luteolus
1 MN215370 Lactifluus sp. 32 HL 2019
2 MH016826 Lactarius sp. in Russulaceae
1 KC424529 Lactifluus
2 KR364016 Lactifluus luteolus
2 KC154098 Lactifluus cf. luteolus KUN F73547
7 MN215371 Lactifluus sp. 21 HL 2019
6 KR364064 Lactifluus albomembranaceus
1 HG917382 Lactifluus foetens
2 UDB013972|103864 Lactarius
1 HG426472 Lactifluus gymnocarpus
1 UDB004358|FR731738|L5119 Russ Cam36 Lactifluus
1 KR364078 Lactifluus flammans
1 UDB013986|103885 Fungi
4 UDB0800133|CS5286 Lactifluus
1 UDB007724|FR731357|L7636 Russ MAD07 Russulaceae
12 LM999910 Lactifluus sp. C1819
2 UDB004452|FR731950|L6612 Russ STP3 Lactifluus
2 FR731894 Lactarius
1 KR819093 Lactifluus
4 UDB0800084|CS4648 Russula
30 KP691413 Lactifluus cf. putidus LIP Mart1113
1 MK774805 Lactarius coccolobae
3 MK046805 Lactifluus sp. LD 2018a
6 MK046850 Lactifluus nebulosus
3 OWN G2252 Lactifluus caribaeus
1 GU258297 Lactarius chiapanensis
10 MK046855 Lactifluus murinipes
1 OWN G5291 Lactifluus byssinus nom. prov.
2 JN168728 Lactifluus
1 OWN G4360 Lactifluus cf. panuoides
1 KJ786694 Lactifluus sp. G3185
5 KP691414 Lactifluus sp. 2 JH 2015
2 JX266621 Russula flocktonae
1 EU019924 Lactifluus clarkeae
7 HQ318282 Lactifluus clarkeae
1 KY774240 Lactifluus sp.
1 KR364095 Lactifluus subclarkeae
1 LC271308 Lactifluus
2 GU222280 Lactifluus clarkeae
7 OWN MAN BZL16 Lactarius panuoides
4 KT289975 Lactifluus
106
35 30 25 20 15 10 5 [mya]

Figure S3 ai

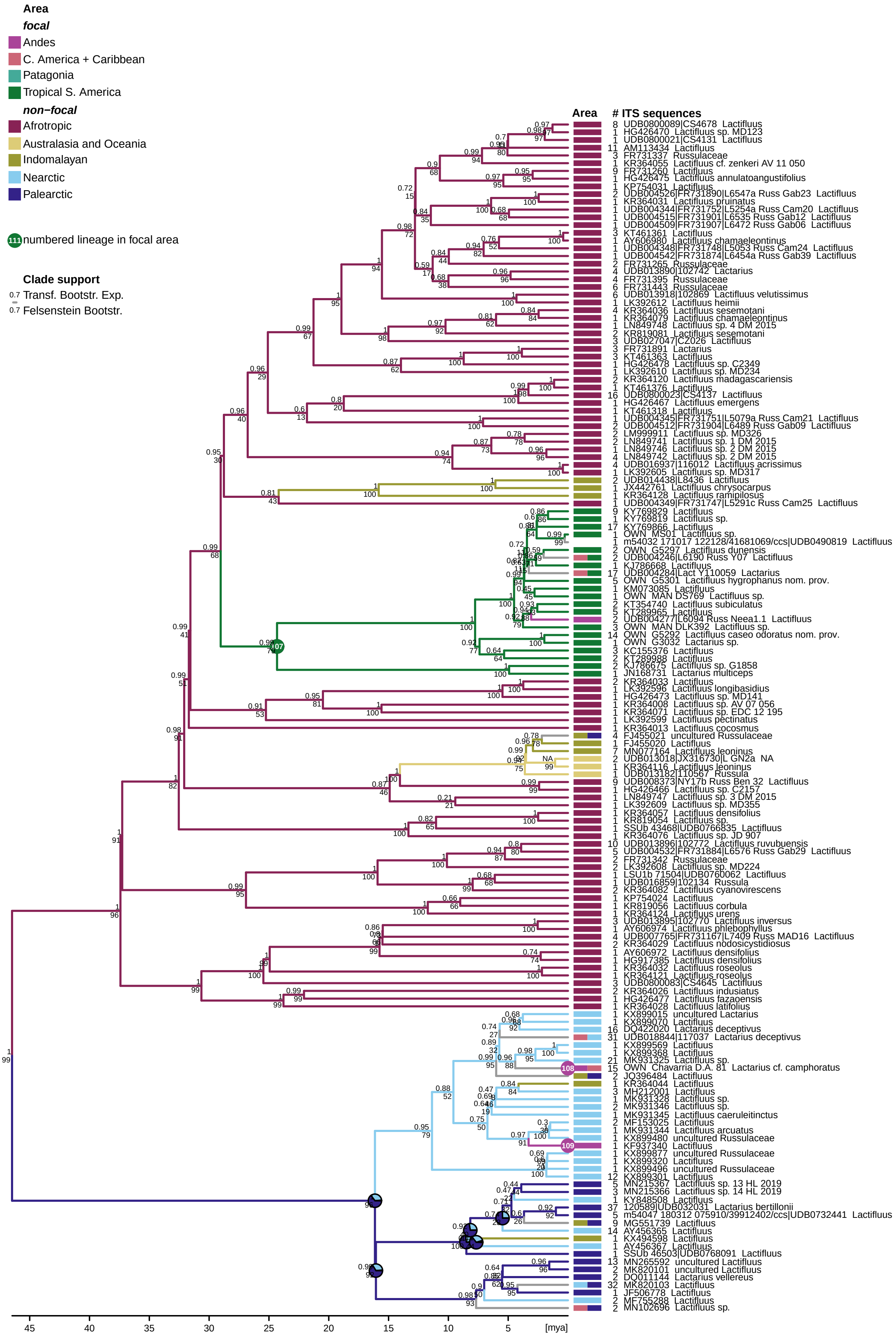
Area
focal
Andes
C. America + Caribbean
Patagonia
Tropical S. America
non−focal
Afrotropic
Australasia and Oceania
Indomalayan
Nearctic
Palearctic
111 numbered lineage in focal area
Clade support
0.7 Transf. Bootstr. Exp.
0.7 Felsenstein Bootstr.
107
108
109
45
40
35
30
25
20
15
10
5
[mya]
Area
ITS sequences
8 UDB0800089|CS4678 Lactifluus
1 HG426470 Lactifluus sp. MD123
1 UDB0800021|CS4131 Lactifluus
11 AM113434 Lactifluus
3 FR731337 Russulaceae
1 KR364055 Lactifluus cf. zenkeri AV 11 050
9 FR731260 Lactifluus
1 HG426475 Lactifluus annulatoangustifolius
1 KP754031 Lactifluus
2 UDB004526|FR731890|L6547a Russ Gab23 Lactifluus
1 KR364031 Lactifluus pruinatus
1 UDB004344|FR731752|L5254a Russ Cam20 Lactifluus
1 UDB004515|FR731901|L6535 Russ Gab12 Lactifluus
1 UDB004509|FR731907|L6472 Russ Gab06 Lactifluus
3 KT461361 Lactifluus
1 AY606980 Lactifluus chamaeleontinus
1 UDB004348|FR731748|L5053 Russ Cam24 Lactifluus
1 UDB004542|FR731874|L6454a Russ Gab39 Lactifluus
2 FR731265 Russulaceae
4 UDB013890|102742 Lactarius
4 FR731395 Russulaceae
6 FR731443 Russulaceae
6 UDB013918|102869 Lactifluus velutissimus
1 LK392612 Lactifluus heimii
4 KR364036 Lactifluus sesemotani
1 KR364079 Lactifluus chamaeleontinus
1 LN849748 Lactifluus sp. 4 DM 2015
2 KR819081 Lactifluus sesemotani
3 UDB027047|CZ026 Lactifluus
3 FR731891 Lactarius
3 KT461363 Lactifluus
1 HG426478 Lactifluus sp. C2349
1 LK392610 Lactifluus sp. MD234
2 KR364120 Lactifluus madagascariensis
1 KT461376 Lactifluus
16 UDB0800023|CS4137 Lactifluus
1 HG426467 Lactifluus emergens
1 KT461318 Lactifluus
1 UDB004345|FR731751|L5079a Russ Cam21 Lactifluus
2 UDB004512|FR731904|L6489 Russ Gab09 Lactifluus
2 LM999911 Lactifluus sp. MD326
2 LN849741 Lactifluus sp. 1 DM 2015
1 LN849746 Lactifluus sp. 2 DM 2015
4 LN849742 Lactifluus sp. 2 DM 2015
4 UDB016937|116012 Lactifluus acrissimus
1 LK392605 Lactifluus sp. MD317
2 UDB014438|L8436 Lactifluus
1 JX442761 Lactifluus chrysocarpus
1 KR364128 Lactifluus ramipilosus
1 UDB004349|FR731747|L5291c Russ Cam25 Lactifluus
9 KY769829 Lactifluus
1 KY769819 Lactifluus sp.
17 KY769866 Lactifluus
1 OWN MS01 Lactifluus sp.
1 m54032 171017 122128/41681069/ccs|UDB0490819 Lactifluus
2 OWN G5297 Lactifluus dunensis
2 UDB004246|L6190 Russ Y07 Lactifluus
1 KJ786668 Lactifluus
17 UDB004284|Lact Y110059 Lactarius
5 OWN G5301 Lactifluus hygrophanus nom. prov.
1 KM073085 Lactifluus
1 OWN MAN DS769 Lactifluus sp.
2 KT354740 Lactifluus subiculatus
5 KT289965 Lactifluus
2 UDB004277|L6094 Russ Neea1.1 Lactifluus
3 OWN MAN DLK392 Lactifluus sp.
14 OWN G5292 Lactifluus caseo odoratus nom. prov.
1 OWN G3032 Lactarius sp.
3 KC155376 Lactifluus
2 KT289988 Lactifluus
2 KJ786675 Lactifluus sp. G1858
1 JN168731 Lactarius multiceps
2 KR364033 Lactifluus
1 LK392596 Lactifluus longibasidius
1 HG426473 Lactifluus sp. MD141
1 KR364008 Lactifluus sp. AV 07 056
1 KR364071 Lactifluus sp. EDC 12 195
1 LK392599 Lactifluus pectinatus
1 KR364013 Lactifluus cocosmus
4 FJ455021 uncultured Russulaceae
1 FJ455020 Lactifluus
7 MN077164 Lactifluus leoninus
2 UDB013018|JX316730|L GN2a NA
1 KR364116 Lactifluus leoninus
1 UDB013182|110567 Russula
9 UDB008373|NY17b Russ Ben 32 Lactifluus
1 HG426466 Lactifluus sp. C2157
1 LN849747 Lactifluus sp. 3 DM 2015
1 LK392609 Lactifluus sp. MD355
1 KR364057 Lactifluus densifolius
1 KR819054 Lactifluus sp.
1 SSUb 43468|UDB0766835 Lactifluus
1 KR364076 Lactifluus sp. JD 907
10 UDB013896|102772 Lactifluus ruvubuensis
5 UDB004532|FR731884|L6576 Russ Gab29 Lactifluus
2 FR731342 Russulaceae
2 LK392608 Lactifluus sp. MD224
1 LSU1b 71504|UDB0760062 Lactifluus
1 UDB016859|102134 Russula
2 KR364082 Lactifluus cyanovirescens
1 KP754024 Lactifluus
1 KR819056 Lactifluus corbula
1 KR364124 Lactifluus urens
3 UDB013895|102770 Lactifluus inversus
1 AY606974 Lactifluus phlebophyllus
4 UDB007765|FR731167|L7409 Russ MAD16 Lactifluus
2 KR364029 Lactifluus nodosicystidiosus
1 AY606972 Lactifluus densifolius
1 HG917385 Lactifluus densifolius
1 KR364032 Lactifluus roseolus
1 KR364121 Lactifluus roseolus
3 UDB0800083|CS4645 Lactifluus
2 KR364026 Lactifluus indusiatus
1 HG426477 Lactifluus fazaoensis
1 KR364028 Lactifluus latifolius
1 KX899015 uncultured Lactarius
1 KX899070 Lactifluus
16 DQ422020 Lactarius deceptivus
31 UDB018844|117037 Lactarius deceptivus
1 KX899569 Lactifluus
1 KX899368 Lactifluus
21 MK931325 Lactifluus sp.
15 OWN Chavarria D.A. 81 Lactarius cf. camphoratus
2 JQ396484 Lactifluus
1 KR364044 Lactifluus
3 MH212001 Lactifluus
1 MK931328 Lactifluus sp.
2 MK931346 Lactifluus sp.
1 MK931345 Lactifluus caeruleitinctus
2 MF153025 Lactifluus
1 MK931344 Lactifluus arcuatus
1 KX899480 uncultured Russulaceae
1 KF937340 Lactifluus
1 KX899877 uncultured Russulaceae
1 KX899320 Lactifluus
1 KX899496 uncultured Russulaceae
12 KX899301 Lactifluus
5 MN215367 Lactifluus sp. 13 HL 2019
3 MN215366 Lactifluus sp. 14 HL 2019
1 KY848508 Lactifluus
37 120589|UDB032031 Lactarius bertillonii
5 m54047 180312 075910/39912402/ccs|UDB0732441 Lactifluus
9 MG551739 Lactifluus
14 AY456365 Lactifluus
1 KX494598 Lactifluus
1 AY456367 Lactifluus
1 SSUb 46503|UDB0768091 Lactifluus
13 MN265592 uncultured Lactifluus
2 MK820101 uncultured Lactifluus
2 DQ011144 Lactarius vellereus
32 MK820103 Lactifluus
1 JF506778 Lactifluus
2 MF755288 Lactifluus
2 MN102696 Lactifluus sp.

Figure S3 aj

Area

focal

Andes
C. America + Caribbean
Patagonia
Tropical S. America

non−focal

Afrotropic
Australasia and Oceania
Indomalayan
Nearctic
Palearctic

111 numbered lineage in focal area

Clade support

0.7 Transf. Bootstr. Exp.
0.7 Felsenstein Bootstr.

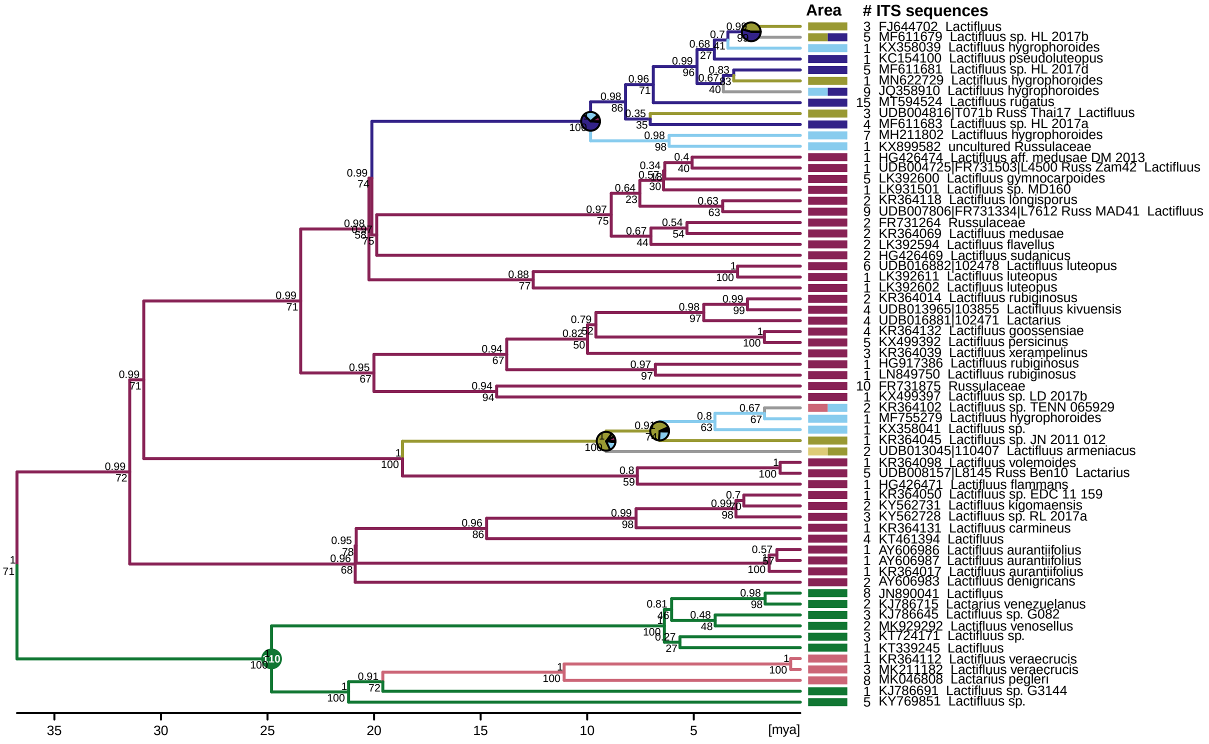

Supplement: Supplementary file 3 — Fig. S3 Detailed Russulaceae supertree. Please note: Wiley Blackwell are not responsible for the content or functionality of any Supporting Information supplied by the authors. Any queries (other than missing material) should be directed to the New Phytologist Central Office. [file NPH-236-698-s002.pdf]
